# Peptidomimetic Modified Heptamethine Cyanine Dyes for Enhanced Bioimaging Targetability: A Molecular Interaction Study on Bovine Serum Albumin and Human Parvalbumin

**DOI:** 10.1021/acs.jmedchem.5c03388

**Published:** 2026-03-20

**Authors:** Tarek E. Ahmed, Nasibeh Azizi-Khereshki, Maged Henary

**Affiliations:** † Department of Chemistry, 1373 Georgia State University, Atlanta 30303, Georgia; ‡ Center for Diagnostics and Therapeutics, Georgia State University, Atlanta 30303, Georgia

## Abstract

Cyanine dyes have found great applications in bioimaging due to their NIR-emitting capabilities. In this work, six heptamethine cyanine dyes **(TEA1–6)** were designed, synthesized, and photophysically studied. While they had strong absorption, their fluorescence was quenched in aqueous solutions. The dyes incorporated amide linkages and amino acid moieties, intended to mimic the peptide bonds to potentially improve biological interactions. This hypothesis was tested by examining the potential interactions between the dyes and two common biological proteins, bovine serum albumin (BSA) and human parvalbumin (HPA). Interestingly, the dyes’ nonfluorescent behavior in aqueous solutions was reversed upon the addition of the proteins BSA or HPA. This was hypothesized to be due to the binding interactions with these proteins and the disruption of the aggregates formed in aqueous solutions. These findings showed that peptide-like substituents could help promote protein recognition and open the horizon for more implementation in biomedical applications

## Graphical abstract

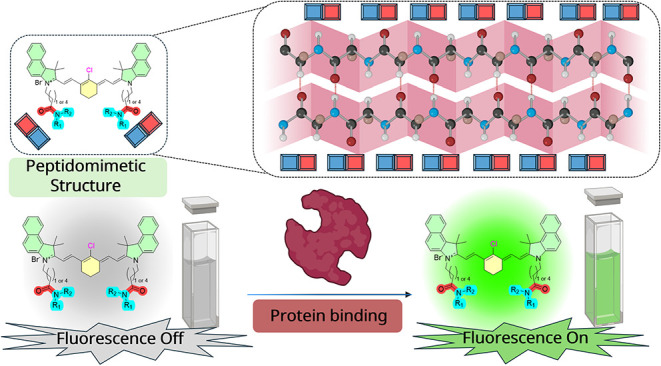

## Introduction

Cyanine dyes represent a significant class of synthetic organic compounds, renowned for their striking, vibrant colors and remarkable ability to interact with light.^1–7^ Fundamentally, their chemical architecture consists of two nitrogen-containing heterocyclic rings connected by a polymethine chain, the length of which plays a crucial role in determining the dye’s specific color and its light absorption and emission behavior.^3,4,8,9^ These molecules are particularly valued for their intense absorption of light, especially within the visible and near-infrared (NIR) regions of the spectrum, and many exhibit strong fluorescence, emitting light at longer wavelengths.^10–13^ Furthermore, the fluorescent characteristics of certain cyanines can be sensitive to their immediate environment, allowing them to function as indicators for changes in polarity, viscosity, or the presence of specific biomolecules.^6,14,15^ For example, they have been extensively employed as fluorescent labels for visualizing biomolecules such as DNA,^16,17^ RNA,^18^ proteins,^19^ and entire cells, proving invaluable for *in vivo* imaging and diagnostics due to their brightness and NIR fluorescence capabilities that minimize interference from biological tissue autofluorescence.^20,21^

Amide coupling is a cornerstone technique in the synthesis and modification of cyanine dyes.^22–24^ By strategically incorporating amine or carboxylic acid groups into cyanine dye precursors, researchers can introduce diverse functionalities, such as targeting moieties, bioconjugation sites, or solubilizing groups, or groups that enhance pharmacokinetic profiles.^25,26^

Bovine serum albumin (BSA) is a globular protein that serves as a foundational tool in biophysical and biomedical research.^27,28^ Owing to its high abundance, stability, and significant sequence and structural homology to human serum albumin (HSA), BSA is frequently employed as an archetypal model for plasma proteins.^29^ The interaction between BSA and near-infrared (NIR) fluorophores provides a powerful system for spectroscopic analysis.^30,31^ NIR probes can associate with specific hydrophobic domains on the albumin surface through noncovalent forces like hydrophobic and electrostatic interactions.^32,33^ A significant consequence of this binding event is a pronounced alteration in the fluorophore’s photophysical properties. Typically, the fluorescence quantum yield is substantially enhanced upon association with the protein.^33,34^ The well-characterized interaction between a novel NIR fluorophore and BSA serves as a crucial *in vitro* surrogate for predicting its behavior *in vivo*. A demonstrated high affinity for BSA strongly suggests that the fluorophore will similarly bind to HSA within the human bloodstream, effectively utilizing albumin as a natural carrier vehicle.^30^ Therefore, assessing the interaction with BSA represents a fundamental and indispensable step in the preclinical evaluation of new NIR agents, offering vital insights into their potential for targeted biological imaging.^35,36^

Human parvalbumin (HPA) is an essential EF-hand calcium-binding protein that governs calcium (Ca^2+^) buffering in fast-twitch muscle fibers and specific neuronal populations.^37,38^ Its physiological function is intrinsically linked to a pronounced conformational transition upon binding Ca^2+^, shifting between a calcium-free (apo) and calcium-bound (holo) state.^39,40^ This dynamic structural change presents a sophisticated target for molecular probes. A strategically designed near-infrared (NIR) fluorophore could be engineered to bind preferentially to a specific conformational state of HPA, creating a probe whose fluorescence is directly modulated by intracellular calcium transients.

Herein, we report the synthesis of amide-substituted heptamethine cyanine dyes. The starting cyanine core was the benz­[e]­indolium ring known for its long wavelength of absorbance,^41,42^ which was substituted with hexanoic or propanoic acid. The acids were coupled with different amines, namely, diethylamine, tyramine, and tryptamine, to form the amide-substituted heptamethine cyanine fluorophores **TEA1–6**. The amines used were chosen to add amino acid-like moieties to the structure of the fluorophore to enhance their interaction with biomolecules such as proteins. The physicochemical properties of these fluorophores were predicted using Chemaxon MarvinSketch,^43^ and the optical properties were studied in four different solvents, which were ethanol, DMSO, HEPES, and PBS buffers. Density-functional theory (DFT) calculations were also done to predict the fluorophores’ frontier molecular orbitals. In addition, polarity studies were conducted to observe the effect of changing the solvent polarity on the dyes’ absorbance and fluorescence. Photothermal stability studies were also performed to test their stability under continuous irradiation with light, relevant to their use for bioimaging applications.

The potential binding of the designed fluorophores with different proteins was predicted by docking and then tested experimentally. The proteins used were bovine serum albumin (BSA) and human parvalbumin (HPA), and the experiment was conducted by monitoring the dye fluorescence enhancement in buffer solution. A binding kinetic study was performed to show how the binding was affected by time and to determine its rate constant and half-life. The added amino acid moieties, including tyramine and tryptamine, along with the amide bond formed enhanced the resemblance of the fluorophores to peptides, which increased the possibilities for potential interactions between the synthesized fluorophores and proteins inside the body, and so amplified their inherent targeting abilities and usefulness in bioimaging and other biomedical applications

## Results and Discussion

### Synthesis

The synthetic pathway is shown in Scheme 1. The benz­[*e*]­indole derivative **A** was allowed to react with 6-bromohexanoic acid or 3-bromopropanoic acid **B1,2** in 1,2-dichlorobenzene to form the hexanoate- or propanoate-substituted benz­[*e*]­indolium salt **C1,2,** respectively. The following step was the amide coupling, which was carried out by reacting the carboxylic acid-substituted benz­[*e*]­indolium salts **C1,2** with different amines in dichloromethane at 70 °C, in the presence of hexafluorophosphate benzotriazole tetramethyl uronium (HBTU) as a coupling reagent to form the amide-substituted benz­[*e*]­indolium salts **D1–6**.

**Scheme 1:**
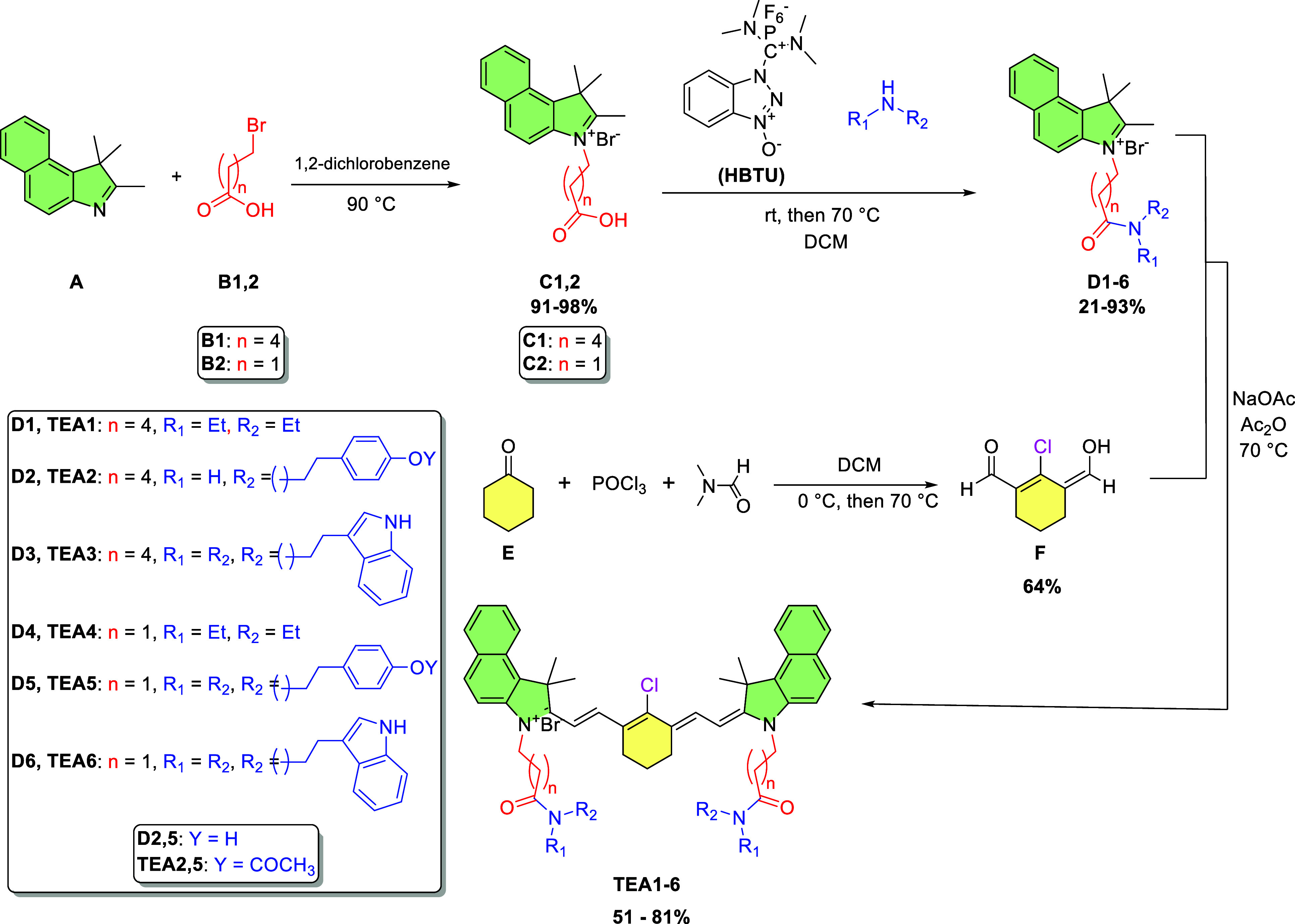
Synthesis of the Peptidomimetic Heptamethine Cyanine Fluorophores **TEA1–6**

The linker for the heptamethine cyanine dyes was obtained through the Vilsmeier–Haack chloroformylation of cyclohexanone **E** using phosphorus oxychloride (POCl_3_) and dimethylformamide (DMF) to obtain dialdehyde linker **F**. The amide benz­[*e*]­indolium salts **D1–6** were then condensed with the Vilsmeier dialdehyde linker **F** under basic conditions to yield the heptamethine fluorophores **TEA1–6**. Interestingly, for the tyramine-substituted benz­[*e*]­indolium salts (**D2,5)**, the phenolic hydroxy groups of tyramine were acetylated by acetic anhydride during the synthesis of the heptamethine cyanine dyes **TEA2,5**. Nevertheless, this did not cause a drastic alteration to the tyrosine-like structure.

### Physicochemical Properties

The physicochemical properties of the synthesized fluorophores **TEA1–6** (shown in Table 1) were predicted using the Chemaxon Marvinsketch software to understand their characteristics and how they can affect their interaction with the surrounding environment. The distribution coefficient (Log D) was predicted to range from 6.91 to 11.61, which shows the high hydrophobicity of the synthesized fluorophores.^12,44^ The surface area, total polar surface area (TPSA), and volume were high for all fluorophores due to their large structures and high molecular weights. These geometrical descriptors were very beneficial, especially for understanding the biological applications of these fluorophores and their interaction with different biomolecules.^45–47^ Fluorophores with larger substituents and larger alkyl chains had the largest values of these descriptors, so **TEA3** had the highest surface area of 1645 Å^2^, and the largest volume of 1009.49 Å^3^. The number of hydrogen bond donor (HBD) and hydrogen bond acceptor (HBA) sites was counted. Their count was important as they were crucial for making hydrogen bonds with biomolecules and with the solvents that could help in the interactions and solubilization of the fluorophores, respectively.^48,49,50,51^ The fluorophores with the tyramine moiety **TEA2** and **TEA5** had 9 HBA sites, which was the highest HBA count due to their extra oxygen atoms. The HBD count was highest in the fluorophores with tryptamine moieties **TEA3** and **TEA6** as they had an extra indole ring that contained an amino group. Finally, polarizability is a property that shows how the electron cloud of the compound could be affected by external electric fields and it is crucial for determining the interaction of the fluorophore with the environment, which affects its optical properties.^51,52^ Polarizability values were high, ranging from 97.2 to 131, due to the presence of various oxygen and nitrogen atoms that could form strong dipoles.

**Table 1:** Physicochemical Properties of the Synthesized Dyes as Predicted by Chemaxon Marvinsketch^a^

Dye	MW	Log D	Rotatable bonds	Surface Area (Å^2^)	TPSA (Å^2^)	Volume (Å^3^)	HBD/HBA	Polarizability
**TEA1**	974.61	9.26	19	1,481	46.9	884	0/5	108
**TEA2**	1186.77	10.63	25	1,725	117	1,046	2/9	129
**TEA3**	1148.77	11.61	21	1,645	96.0	1,009	4/5	131
**TEA4**	890.45	6.91	13	1,303	46.9	784	0/5	97.2
**TEA5**	1102.61	8.27	19	1,539	117	943	2/9	118
**TEA6**	1064.61	9.25	15	1,462	96.0	906	4/5	120

a Log D: Distribution coefficient was calculated at pH = 7.4; MW, molecular weight; TPSA, total polar surface area; HBD, H-bond donor; HBA, H-bond acceptor.

### Optical Properties

The optical properties of the synthesized fluorophores **TEA1–6** were studied in different solvents as shown in Table 2. The four solvents included ethanol and dimethyl sulfoxide (DMSO) as examples of organic solvents to understand the properties of the dye and compare them to those of other fluorophores. Additionally, two water-based buffers were used, (4-(2-Hydroxyethyl)­piperazine-1-ethane-sulfonic acid) (HEPES buffer) and phosphate-buffered saline (PBS buffer), to mimic the environment inside the body and the blood to be relevant to the biological applications.^53,54^ The properties in the four solvents were compared to those of indocyanine green (ICG) as it is the only FDA-approved cyanine fluorophore to date.^55–57^

**Table 2:** Optical Properties of the Synthesized Fluorophores **TEA1-6** and ICG in Different Solvents

Dye	Solvent	Absorbance Wavelength Maxima (nm)	Excitation Wavelength (nm)	Emission Wavelength (nm)	Stokes Shift (nm)	Extinction coefficient (M^–1^ cm^–1^)	Quantum Yield of Fluorescence (%)	Molecular Brightness (M^–1^ cm^–1^)
**TEA1**	Ethanol	824	750	839	15	201,700	1.03	2,081
	DMSO	828	755	845	17	107,100	5.18	5,550
	HEPES Buffer	760, 824	-	-	-	66,500	-	-
	PBS Buffer	770, 840	-	-	-	59,000	-	-
**TEA2**	Ethanol	826	750	841	15	142,100	2.07	5,376
	DMSO	840	755	854	14	148,200	6.22	9,222
	HEPES Buffer	750, 844	-	-	-	161,700	-	-
	PBS Buffer	750, 848	-	-	-	60,300	-	-
**TEA3**	Ethanol	828	750	848	20	169,200	2.69	4,544
	DMSO	846	755	860	14	154,200	7.48	11,541
	HEPES Buffer	760, 844	-	-	-	160,100	-	-
	PBS Buffer	760, 849	-	-	-	105,900	-	-
**TEA4**	Ethanol	826	750	840	14	171,800	2.94	5,046
	DMSO	832	755	847	15	111,500	7.37	8,218
	HEPES Buffer	750, 832	-	-	-	56,500	-	-
	PBS Buffer	750, 818	-	-	-	52,900	-	-
**TEA5**	Ethanol	830	750	847	17	134,000	3.67	4,915
	DMSO	842	755	858	16	102,500	7.54	7,725
	HEPES Buffer	760, 844	-	-	-	58,200	-	-
	PBS Buffer	760, 846	-	-	-	53,000	-	-
**TEA6**	Ethanol	830	750	849	19	187,900	1.70	3,191
	DMSO	842	755	858	16	125,500	5.25	6,587
	HEPES Buffer	750, 844	-		-	69,100	-	-
	PBS Buffer	750, 844	-	-	-	64,100	-	-
ICG	Ethanol	785	710	825	40	215,000	14.0 ^62^	30,100
	DMSO	798	720	817	19	216,000	16.7 ^55^	36,072
	HEPES Buffer	778	720	802	24	148,000	2.9 ^62^	4,292
	PBS Buffer	778	720	802	24	146,000	2.9 ^62^	4,234

The absorbance wavelengths ranged from 818 to 849 nm in the different solvents, which were in the near-infrared region and could be beneficial for bioimaging because of the deeper penetration and decreased background interference from the red-shifted absorbance.^58,59^ In the aqueous buffers, a noticeable blue-shifted peak appeared around 750–770 nm in all of the fluorophores. These peaks were due to the H-aggregation of the fluorophore molecules happening in aqueous buffer because of the π–π stacking of the benzene rings.^60,61^ The excitation wavelengths used were 750 nm or 755 nm, as these were the wavelengths that gave the highest fluorescence signal with the least overlap of the excitation and emission signals. Interestingly, all the fluorophores **TEA1–6** had only fluorescence emission in ethanol and DMSO, and the emission wavelengths were varying from 839 to 860 nm. There were no emission signals in HEPES or PBS buffers despite the trials to change the excitation wavelengths and the concentration of the fluorophores.

This could be attributed to the aggregation-based quenching of fluorescence, where these fluorophores had H-aggregation in these water-based buffer solutions due to their highly hydrophobic nature and the presence of three aromatic rings in their structure, leading to π–π stacking interactions and so causing the aggregation.^60,61^ Another reason could be the solvent effect where polar solvents tend to stabilize the excited states leading to increased nonradiative decay.^63^ These proposed causes for the absence of fluorescence in these buffers were confirmed by examining the fluorescence enhancement in these buffers upon the addition of bovine serum albumin or human parvalbumin, as discussed later in the manuscript. The Stokes shift is the difference between the absorbance and emission wavelength maxima. Its values were relatively small, ranging from 14 to 20 nm. This was attributed to their rigid backbone and fused ring system, which minimized the structural change of the conjugated system in the excited state and fixed the geometry upon excitation leading to the smaller Stokes shift.^64^

The extinction coefficient of the fluorophores ranged from 52,900 to 201,700 M^–1^ cm^–1^. They were the highest in ethanol, followed by DMSO, and the lowest in HEPES and PBS buffers due to H-aggregation. **TEA1** had the highest value in ethanol (201,700 M^–1^ cm^–1^). Fluorophores with the tyramine or tryptamine moieties had higher extinction coefficient values in buffer solutions compared to their diethylamine counterparts (**TEA2 and 3** vs **TEA1**, and **TEA5 and 6** vs **TEA4**). This can be caused by the interactions that the extra oxygen and nitrogen atoms could have with the water molecules, thus decreasing the extent of H-aggregation. The quantum yield of fluorescence was low with values ranging from 1.03% to 7.54%, which could be attributed to the aggregation-based quenching of fluorescence,^65,66^ the efficient nonradiative decay (internal conversion) caused by the flexible side chain structures, and the large number of rotatable bonds.^67,68^ The molecular brightness is the product of the molar extinction coefficient and the quantum yield of the fluorescence. It was calculated, and its values ranged from 2,081 to 5,376 M^–1^ cm^–1^ in ethanol, and from 5,550 to 11,541 M^–1^ cm^–1^ in DMSO. The values were higher in DMSO due to better solubility, reduced aggregation, and enhanced energy loss by emission.

### Hydrophobicity Studies

Owing to the high hydrophobicity of the synthesized fluorophores evidenced by their high predicted log D values, and the aggregation-induced quenching of fluorescence in aqueous buffers, the synthesized fluorophores were tested for hydrophobicity sensing to confirm the H-aggregation of the dyes in aqueous solutions.^69^ This study was based on a similar work previously published to confirm the formation of aggregates in water-based buffers.^70^ This was carried out by measuring both the absorbance and fluorescence of 6 μM solutions of **TEA1–6** in increasing methanol:water ratios. Both water and methanol are polar solvents, but water has a much higher polarity than methanol, which is still considered an organic solvent. Therefore, by increasing the methanol:water ratio, the hydrophobicity is considered to be increased. Since the fluorophores showed almost no fluorescence and broad absorbance spectra in HEPES and PBS buffers, as expected, their absorbance and fluorescence intensity increased as the methanol:water ratio increased due to the enhanced solubility and decreased aggregation (Figures 1, 2, S35, and S36). This confirmed the predicted log D values of these fluorophores, their considerably high hydrophobicity, and their potential use as hydrophobicity sensors by virtue of their totally distinct absorbance and fluorescence behavior in solutions with different polarities.

**Figure 1:**
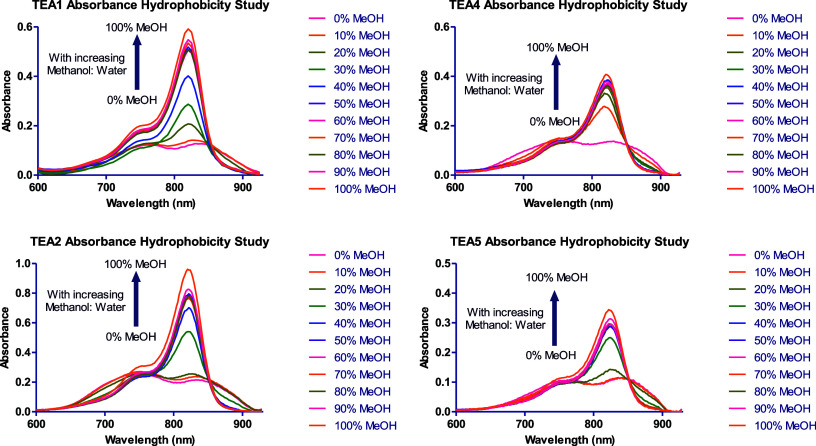
Absorbance spectra of **TEA1, TEA2**, **TEA4**, and **TEA5** in solutions with increasing methanol:water ratios.

**Figure 2:**
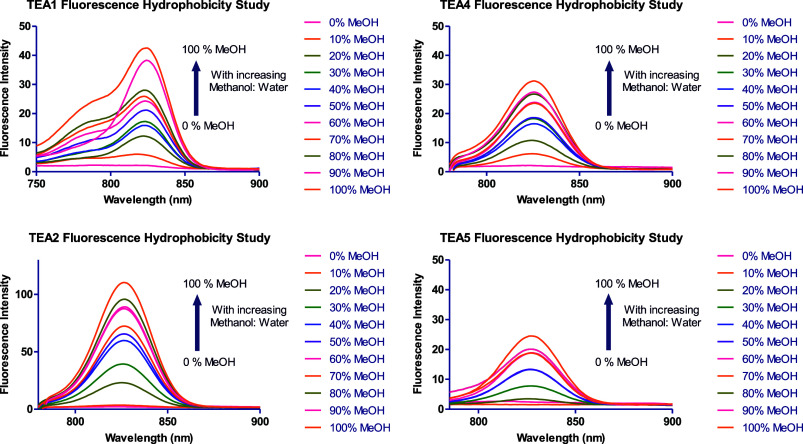
Fluorescence spectra of **TEA1, TEA2**, **TEA4**, and **TEA5** in solutions with increasing methanol:water ratios.

### Photothermal Stability Studies

The synthesized fluorophores were tested for their photothermal stability by measuring their absorbance over time upon continuous irradiation by the light of a UV lamp and comparing them to the reference ICG (Figure 3). For comparison, two groups of the fluorophore solutions were prepared: one was put in the dark and covered with a foil plate, and the other was put under the light of a UV lamp with a wavelength of 254 nm and covered to prevent any other light from interfering with the experiment. The dye solutions (4–11 μM in ethanol) were put 10 cm away from a 6000 mW 254 nm UV lamp at 25 °C. The absorbance was measured for each dye solution in each group at different time intervals for 72 h, and then the normalized absorbance was plotted against time. In dark conditions, there was no noticeable change in the absorbance over the period of 72 h, and all the dyes were stable in the dark. Under light conditions, there were changes in the absorbance demonstrated by a constant decrease in the absorbance for most of the dyes. **TEA1** was the most stable, retaining 87% of its absorbance after 72 h, followed by **TEA2** and **TEA3** keeping 83 and 81%, respectively. **TEA4** was the least stable, retaining 55% of its absorbance over the period of 72 h. ICG also exhibited a decrease in absorbance over time, and its photothermal stability was comparable to that of **TEA1**. Overall, although the fluorophores showed a slight decrease in their absorbance over time, they showed good photothermal stability keeping more than 70% of their absorbance for most of the synthesized fluorophores, analogous to ICG’s stability.

**Figure 3:**
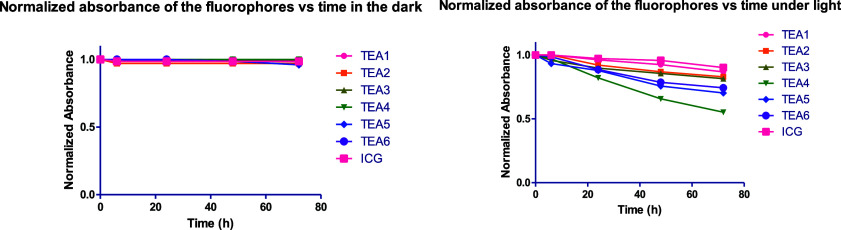
Photothermal stability of fluorophores **TEA1–6**. Their normalized absorbance over time in dark conditions and under irradiation with a 6000 mW 254 nm UV lamp placed 10 cm from the fluorophores’ solution (4–11 μM in ethanol) at 25 °C.

### Computational DFT Studies

The density functional theory (DFT) calculations were conducted for the synthesized fluorophores using Spartan software to observe the effect of photoexcitation on the frontier molecular orbitals of TEA1–6 ^71^ (Figures 4 and S37). Their molecular structures included a benz­[e]­indolium core with a hexanoate or propanoate amide substituent connected together with a polymethine chain, having a chlorine substituent at the meso position. These structural features should affect the energy of the frontier molecular orbitals, including the highest occupied molecular orbital (HOMO) and the lowest unoccupied molecular orbital (LUMO). For **TEA1**, the HOMO was found to have an energy of −6.92 eV and the LUMO had an energy of −4.94 eV making the energy gap between the HOMO and LUMO to be 1.98 eV. The energy gap gave insight into the expected wavelength of absorbance; since the energy gap is inversely proportional to the wavelength of absorbance, the small gap is indicative of the expected large and red-shifted wavelength this fluorophore should have in different solvents, and that matched what was found experimentally.

**Figure 4:**
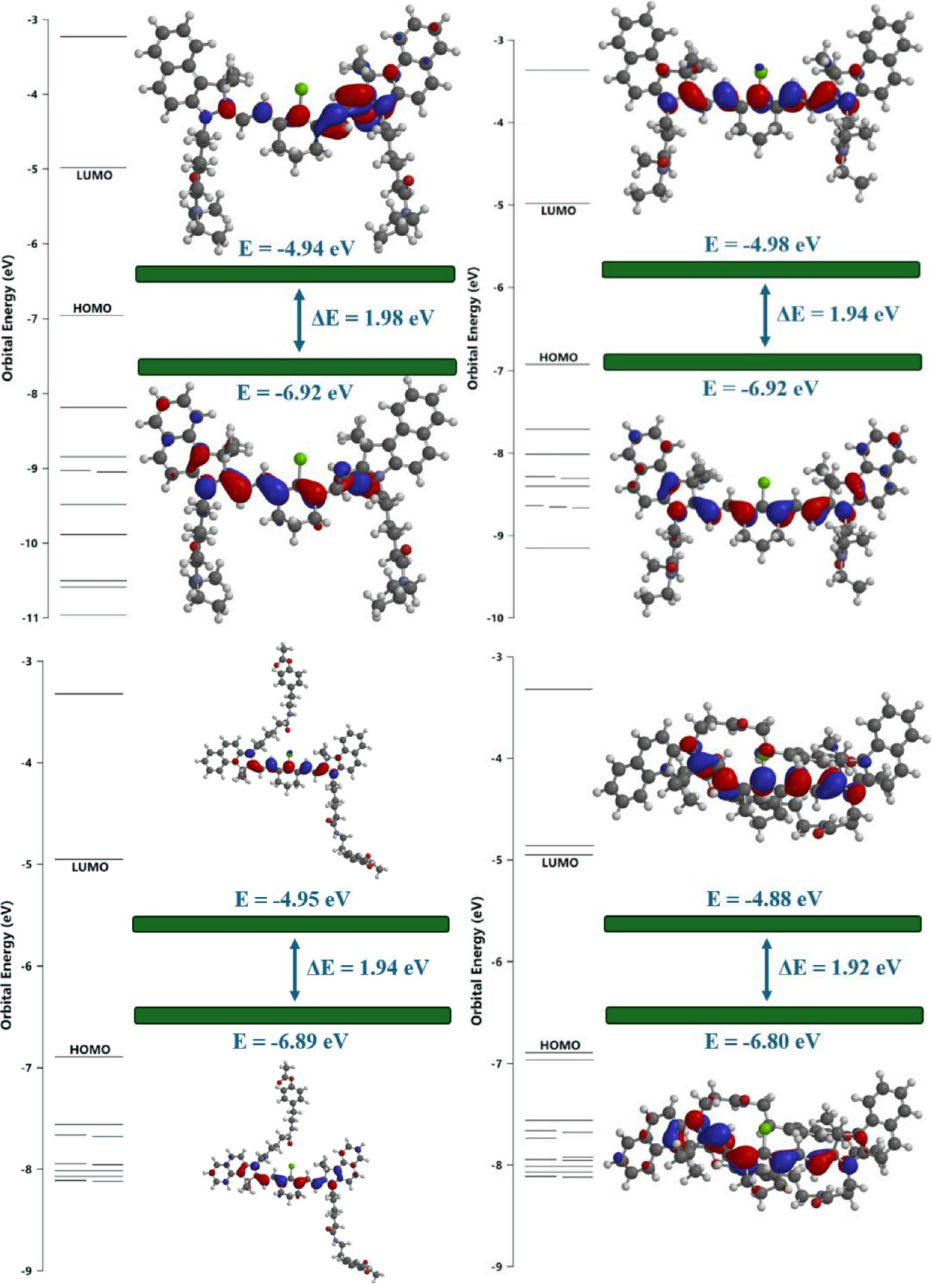
HOMO and LUMO of **TEA1** (top left), **TEA2** (bottom left), **TEA4** (top right), **and TEA5** (bottom right) as predicted by DFT calculations using Spartan software. The difference in energy between HOMO and LUMO was calculated to be 1.98, 1.94, 1.94, and 1.92 eV for **TEA1**, **TEA2, TEA4,** and **TEA5,** respectively.

The expected wavelength maximum of **TEA1** was calculated using the predicted HOMO/LUMO energy gap by inputting it into the Planck–Einstein formula E = hc/λ, where E is the energy, h is Planck’s constant, c is the velocity of light, and λ is the wavelength. The expected wavelength maximum was found to be 626.3 nm, which is around 200 nm below the experimental value. This was the same observation for the other fluorophores **TEA2–6**. This blue shift of the predicted vs experimental wavelength maxima is common for DFT calculations due to their tendency to overestimate excitation energies, their low ability to accurately model charge-transfer processes, and the generation of spurious low-energy “ghost” states with minimal oscillator strength.^72–74^

Interestingly, the hexanoate amide substituent was not part of the frontier molecular orbitals, presumably because it was far from the path of the electron transfer, and that was why it had little effect on the energies of the HOMO and LUMO. For this reason, the other fluorophores had similar trends with close values of the energy gap. **TEA4,** which had the propanoate diethylamide substituent, had a HOMO orbital energy of −6.92 eV and a LUMO orbital energy of −4.98 eV, and the energy gap was calculated to be 1.94 eV. The energy gaps for the other fluorophores were 1.94, 1.79, 1.92, and 1.84 eV for **TEA2**, **TEA3**, **TEA5**, and **TEA6,** respectively. These similar values of energy gaps reflected the fact that the substituents on the indolium ring nitrogen were not part of the HOMO and LUMO orbitals, causing the substituents to have little effect on their energies and so on the energy gaps.

The energies of the 0–0 transitions from the spectral-fluorescence data of the peptidomimetic dyes were calculated by plotting the normalized absorbance and fluorescence spectra of each dye and observing the wavelength at the intersection point between the two spectra (Figure S38). To calculate the energy, the same Planck–Einstein formula (E = hc/λ) was used, and the values ranged from 1.479 to 1.490 eV (Table 3). These observed energies were compared to the calculated energies from the DFT studies, and they were consistently lower than the calculated energies (0.36–0.49 eV difference). This was expected from the DFT studies using the B3LYP functional because, as mentioned earlier, this standard hybrid functional tends to overestimate the energy gaps and cannot accurately capture the extensive delocalization within the cyanine chain, so they systematically predict higher excitation energies.^75,76^

**Table 3:** Observed Wavelength and Energy of the 0–0 Transitions vs Calculated Energy

Dye	Observed wavelength of 0–0 transitions (nm)	Observed energy of 0–0 transitions (eV)	Calculated energy of electronic transitions obtained from quantum chemical calculations (eV)
**TEA1**	832	1.490	1.98
**TEA2**	834	1.487	1.94
**TEA3**	836	1.483	1.79
**TEA4**	834	1.487	1.94
**TEA5**	838	1.479	1.92
**TEA6**	838	1.479	1.84

### Docking to Bovine Serum Albumin (BSA) and Human Parvalbumin (HPA)

By virtue of the peptidomimetic substituents on the synthesized fluorophores, they were expected to have more potential interactions with the peptides and proteins inside the body. Therefore, we wanted to test their interactions with different kinds of proteins in different organisms. Bovine serum albumin was selected as an example of common serum proteins found in animals because it has a high similarity to human serum albumin. In addition, human parvalbumin was selected as an example of a protein that is distributed extravascularly and found in brain and muscle tissues. Before doing the experimental binding studies, the binding of the synthesized fluorophores with these different proteins was predicted with docking studies using PyRx software.^77^ Blind or global docking was used in this study, since the binding sites for the fluorophores inside these two proteins were unknown. The crystal structures of BSA and HPA were downloaded from the Research Collaboratory for Structural Bioinformatics (RCSB) Protein Data Bank website. For bovine serum albumin, the crystal structure with PDB ID 4JK4 was used in the docking study,^78^ and for human parvalbumin, the PDB ID 9BB8 was used.^79^ The structures of the fluorophores were minimized, and the structures of the proteins were prepared prior to the docking studies using PyRx software. The 2D and 3D interactions between the fluorophores and the proteins were visualized using discovery studio^80^ and ChimeraX^81,82^ software, respectively.

Each docking study yielded nine different poses for every fluorophore inside the protein, each of which had its binding affinity score (Table 4). The docking study in BSA showed that the binding affinity scores of the highest ranked poses for the fluorophores ranged from −8.80 to −11.0 kcal/mol. **TEA6** had the highest score of −11.0 kcal/mol followed by **TEA5** with −10.7 kcal/mol. It was noticed that the fluorophores with the shorter alkyl chain **TEA4–6** had higher scores than those with the longer alkyl chains **TEA1–3**, indicating that the binding site preferred a smaller structure and that the binding interactions were better with the relatively smaller fluorophores.

**Table 4:** Binding Affinity Scores of the Synthesized Dyes **TEA1–6** as Predicted by PyRx Software

Dye	Binding affinity score of the highest ranked pose in BSA (kcal/mol)	Binding affinity score of the highest ranked pose in HPA (kcal/mol)
**TEA1**	–8.80	–6.40
**TEA2**	–9.90	–6.70
**TEA3**	–10.1	–7.00
**TEA4**	–9.30	–6.50
**TEA5**	–10.7	–7.60
**TEA6**	–11.0	–7.80

Studying the binding site in detail showed that the fluorophores tend to bind in domains II and III of the bovine serum albumin.^83^ For **TEA1**, the binding interactions included a hydrogen bond with arginine 435 (ARG435), pi–cation interactions with lysine 294 (LYS294) and arginine 198 (ARG198), and a pi–anion interaction with glutamate 443 (GLU443), along with many van der Waals interactions (Figure 5). The distance between the hydrogen atom and the acceptor atom was 2.050 Å, which was in the permissible distance for a strong hydrogen bond (2.0–3.5 Å).^84^ Remarkably, **TEA2** with the tyrosine-like side chain showed better interactions with the binding site (Figure 6) compared to the diethylamine-substituted **TEA1**. It showed two hydrogen bonds with aspartate 450 (ASP450) and arginine 194 (ARG194), in addition to extra pi–alkyl interactions with leucine 237 (LEU237), alanine 290 (ALA290), and proline 446 (PRO446) due to the extra phenyl ring of tyramine.

**Figure 5:**
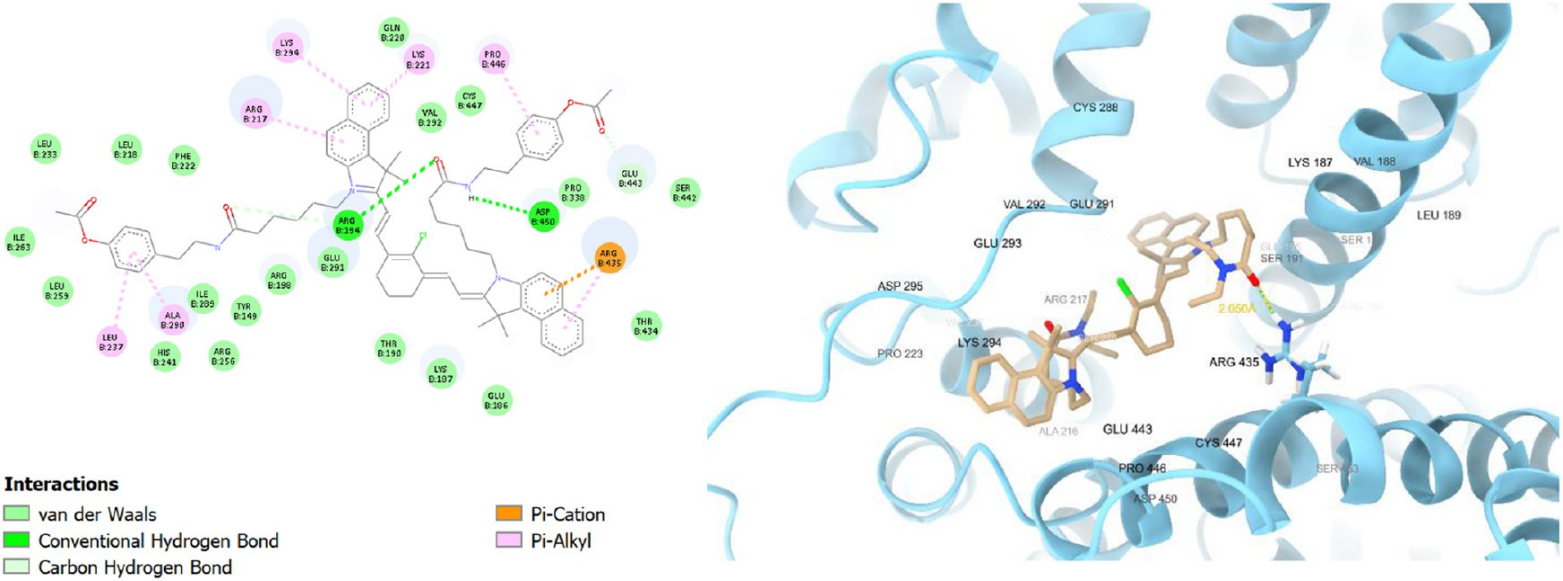
2D and 3D interaction diagrams of **TEA1** with bovine serum albumin (BSA, PDB ID: 4jk4); **TEA1** shown as beige sticks, BSA shown as light blue ribbons, and the hydrogen bond with ARG435 demonstrated as yellow dots with its distance calculated and written in yellow.

**Figure 6:**
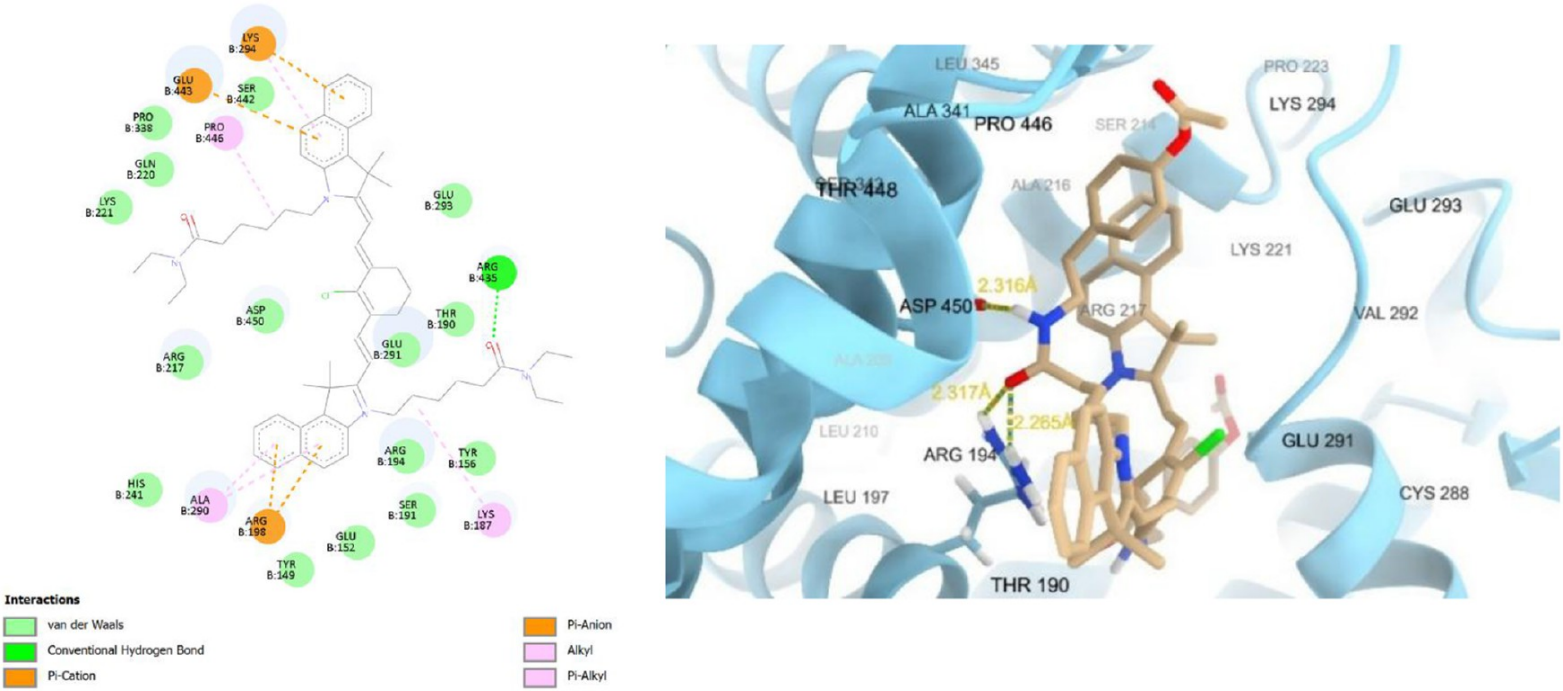
2D and 3D interaction diagrams of **TEA2** with bovine serum albumin (BSA, PDB ID: 4jk4); **TEA2** shown as beige sticks, BSA shown as light blue ribbons, and the hydrogen bonds with ASP450 and ARG194 demonstrated as green dots with their distances calculated and written in yellow.

**TEA3** had the highest binding affinity score in the docking study in the group of the hexanoate amide-substituted dyes, and as expected it showed strong favorable interactions in the form of two hydrogen bonds with aspartate 450 (ASP450) and proline 338 (PRO338), in addition to other pi–cation, pi–anion, and pi–sulfur interactions (Figure S40). **TEA4** with the shorter alkyl chain had similar binding interactions, with a 2.305 Å hydrogen bond with lysine 294 (LYS294), pi–cation interaction with arginine 435 (ARG435), and pi–alkyl interactions with valine 342 (VAL342), glutamate 291 (GLU291), lysine 221 (LYS221), and lysine 187 (LYS187), in addition to other van der Waals interactions (Figure 7). Notably as predicted, **TEA5** had more interactions with the binding site, which is demonstrated by its higher binding affinity score and owing to its tyrosine-like structure (Figure 8). It made two hydrogen bonds with arginine 194 (ARG194) and arginine (ARG256), a pi–alkyl interaction with cysteine 447 (CYS447) and alanine 290 (ALA290), and a pi–cation interaction with arginine 198 (ARG198) using its tyramine moiety. Finally, **TEA6** had the highest binding affinity score, so it showed very strong interactions with the binding site, demonstrated by three hydrogen bonds with arginine 194 (ARG194), arginine 217 (ARG217), and arginine 435 (ARG435), in addition to other pi–anion interactions using its tryptamine moiety (Figure S44).

**Figure 7:**
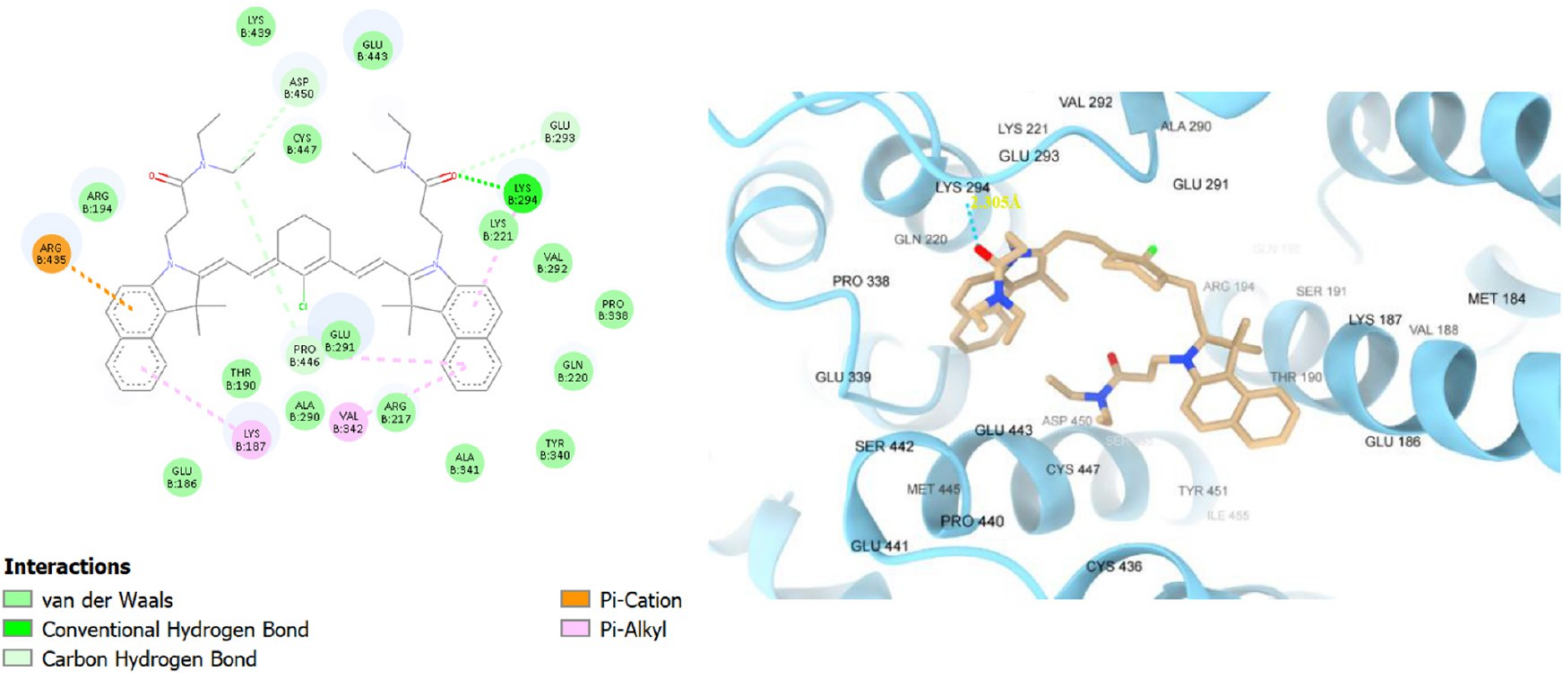
2D and 3D interaction diagrams of **TEA4** with bovine serum albumin (BSA, PDB ID: 4jk4)**; TEA4** shown as beige sticks, BSA shown as light blue ribbons, and the hydrogen bond with LYS294 demonstrated as light blue dots with its distance calculated and written in yellow.

**Figure 8:**
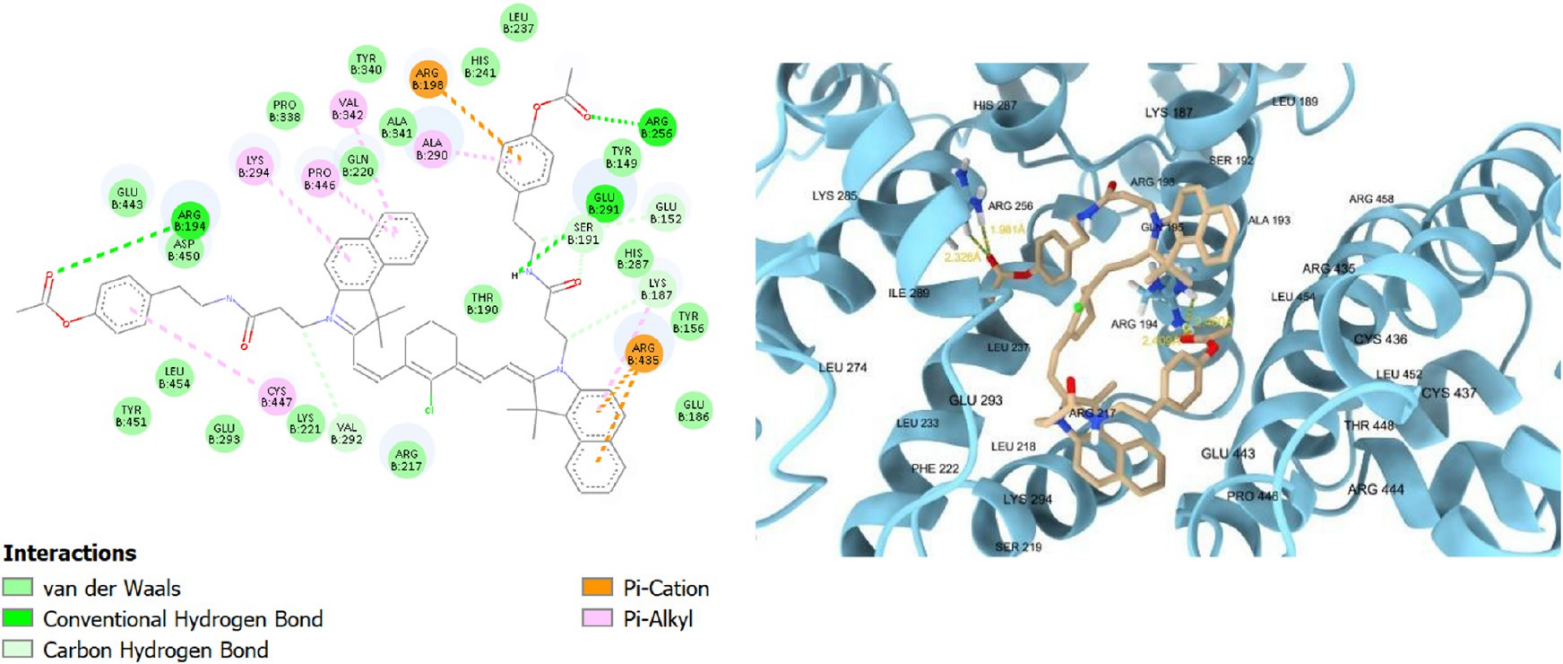
2D and 3D interaction diagrams of **TEA5** with bovine serum albumin (BSA, PDB ID: 4jk4)**; TEA5** shown as beige sticks, BSA shown as light blue ribbons, and the hydrogen bonds with ARG194 and ARG256 demonstrated as green dots with their distances calculated and written in yellow.

Regarding human parvalbumin, the fluorophore binding affinity scores followed a similar trend as in BSA, where they were higher for the shorter chain fluorophores **TEA4–6**, and they ranged from −6.40 to −7.80 kcal/mol. The scores were generally lower than in BSA, which can be attributed to the weaker interactions with HPA, and the smaller size of HPA compared to BSA, which meant tighter binding pockets were available for these relatively large fluorophores. The fluorophores were bound mainly in the CD-domain (the EF-hand 1), which is one of the active calcium-binding sites, with fewer interactions with the other domains (AB and EF domains).

Taking **TEA1** as an example, it had a binding affinity score of −6.40 kcal/mol, and it was found to make pi–pi stacking with phenylalanine 66 (PHE66), pi–cation stacking with lysine 69 (LYS69), and pi–alkyl stacking with lysine 53 (LYS53), along with many van der Waals interactions (Figure 9). Intriguingly, **TEA2** had better interactions with the HPA binding site due to its tyrosine-like structure that allowed for a hydrogen bond to be made with lysine 69 (LYS69), in addition to another hydrogen bond with glycine 1 (GLY1), pi–pi stacking with phenylalanine 66 (PHE66), and pi–alkyl interactions with lysine 46 (LYS46) (Figure 10). **TEA3**, similar to **TEA2**, exhibited strong interactions with the binding site due to the tryptophan-like structure and showed a hydrogen bond with aspartate 62 (ASP62), pi–sigma interaction with lysine 53 (LYS53), and pi–pi stacking with phenylalanine 66 (PHE66) (Figure S48).

**Figure 9:**
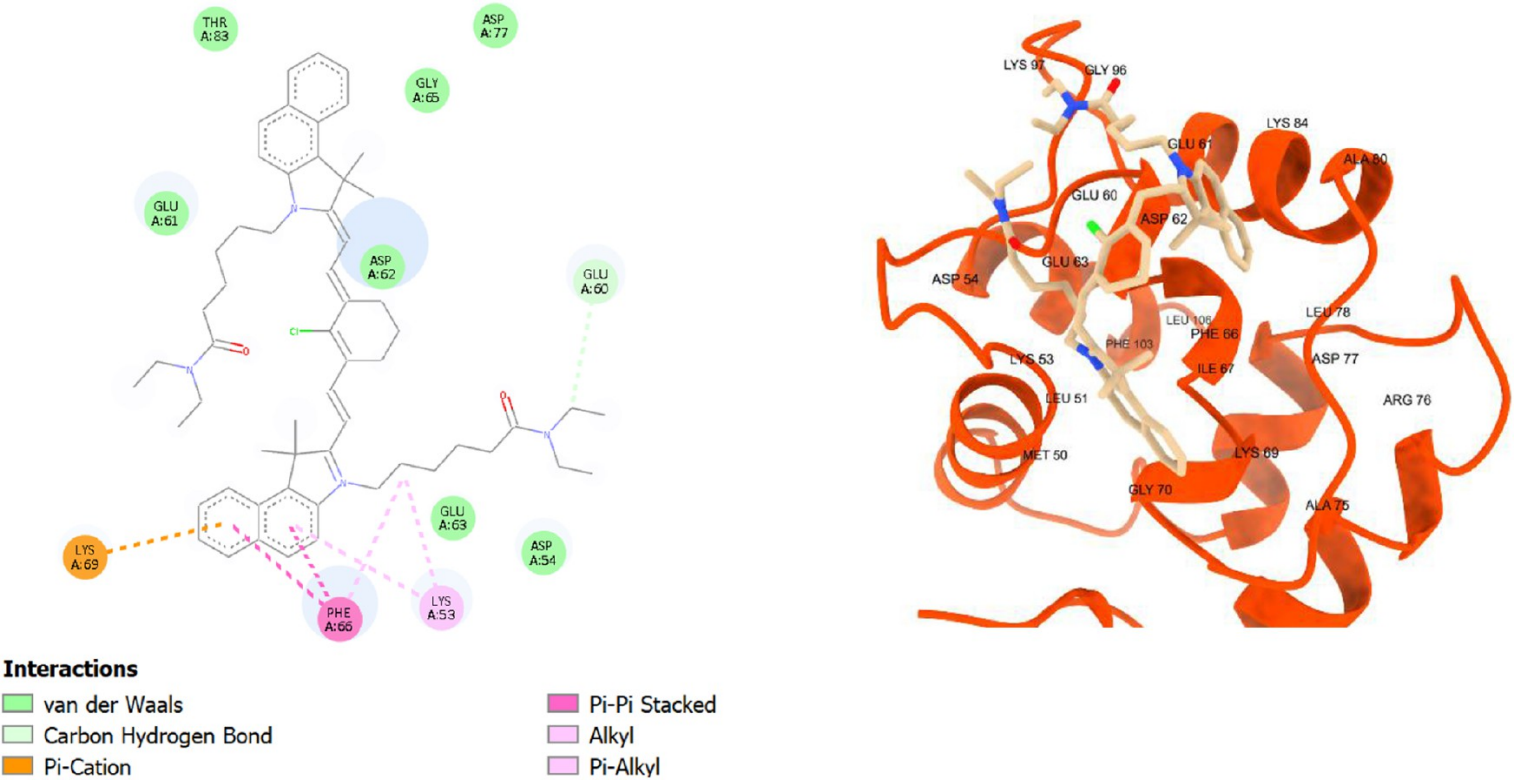
2D and 3D interaction diagrams of **TEA1** with human parvalbumin (HPA, PDB ID: 9bb8); **TEA1** shown as beige sticks and HPA shown as red ribbons.

**Figure 10:**
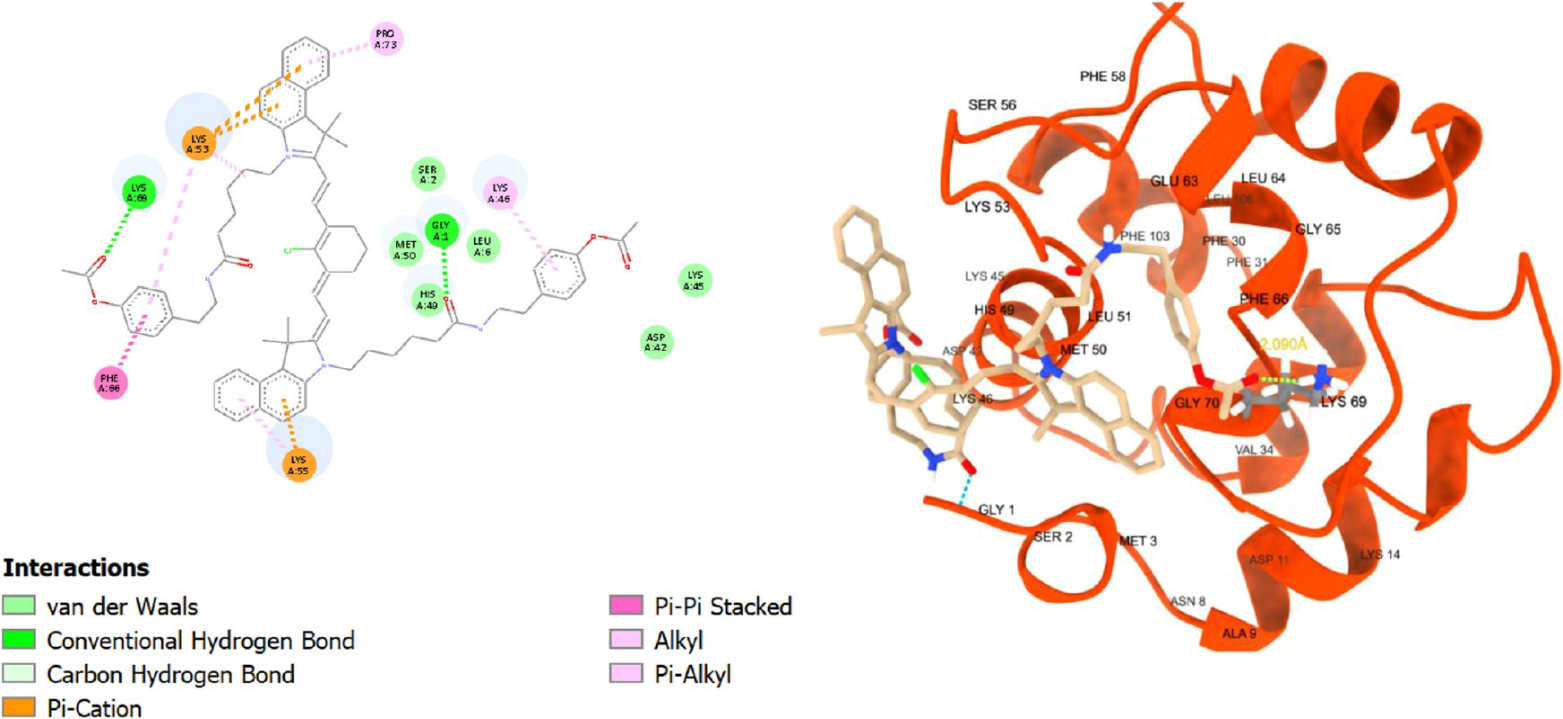
2D and 3D interaction diagrams of **TEA2** with human parvalbumin (HPA, PDB ID: 9bb8); **TEA2** shown as beige sticks, HPA shown as red ribbons, and the hydrogen bonds with GLY1 and LYS69 demonstrated as yellow dots with their distances calculated and written in yellow.

For the smaller **TEA4**, its binding affinity score was slightly higher at −6.50 kcal/mol, and it made similar pi–cation interactions with lysine 69 (LYS69) and pi–alkyl interactions with arginine 76 (ARG76), valine 16 (VAL16), and lysine 13 (LYS13), in addition to other van der Waals interactions (Figure 11). **TEA5**, similar to its longer chain derivative, possessed stronger interactions than TEA4 due to the tyrosine-like moiety, where it showed a hydrogen bond and amide–pi stacking with lysine 53 (LYS53), and another hydrogen bond with pi–alkyl interaction with lysine 69 (LYS69) (Figure 12). Lastly, **TEA6** showed pi–alkyl interaction with lysine 69 (LYS69), pi–cation interaction with lysine 55 (LYS55), and pi–anion interaction with aspartate 62 (ASP62) among other van der Waals interactions (Figure S52).

**Figure 11:**
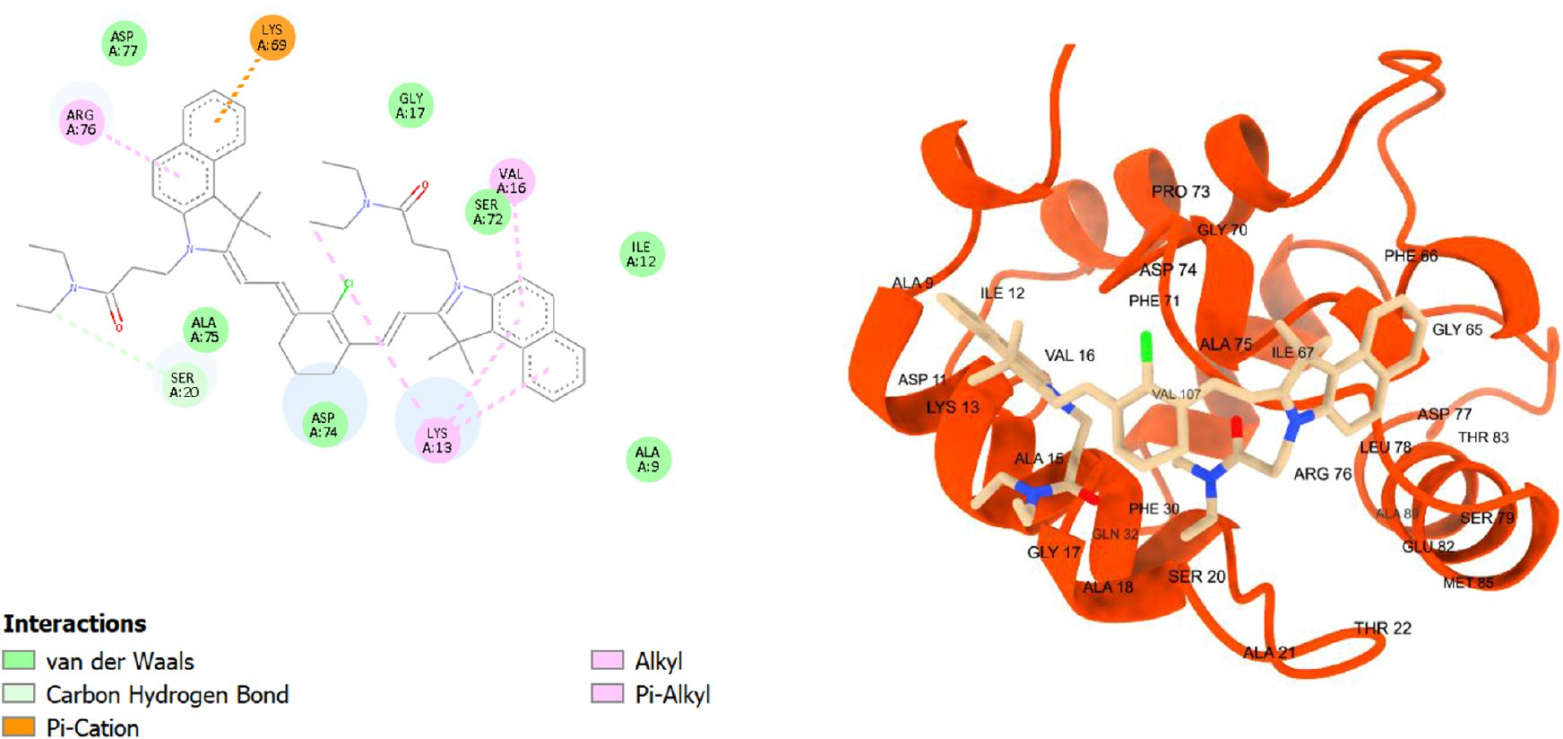
2D and 3D interaction diagrams of **TEA4** with human parvalbumin (HPA, PDB ID: 9bb8); **TEA4** shown as beige sticks, and HPA shown as red ribbons.

**Figure 12:**
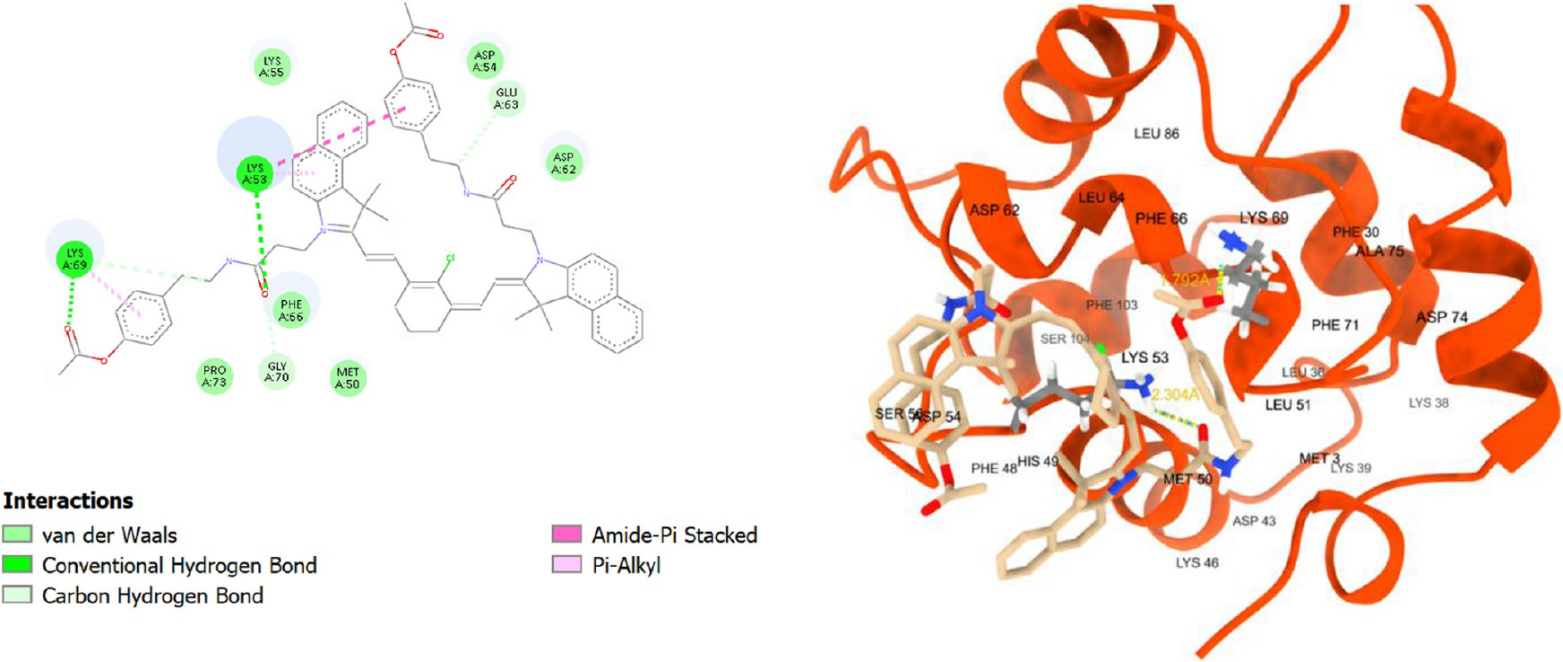
2D and 3D interaction diagrams of **TEA5** with human parvalbumin (HPA, PDB ID: 9bb8); **TEA5** shown as beige sticks, HPA shown as red ribbons, and the hydrogen bonds with LYS53 and LYS69 demonstrated as yellow dots with their distances calculated and written in yellow.

The binding modes of the highest ranked poses of the dyes **TEA1–6** inside the cavities of BSA are shown in Figures S39–S46, and those in HPA are shown in Figures S47–S54. In summary, the dyes possessing the peptidomimetic moieties including tyramine (**TEA2 and TEA5**) or tryptamine **(TEA3 and TEA6)** exhibited higher binding affinity scores and showed stronger interactions with the two tested proteins (BSA and HPA), which verified our hypothesis about adding peptidomimetic substituents to increase the interaction of the fluorophores with proteins, and using this in increasing their target ability.

### Fluorescence Enhancement with Protein Binding

The molecular docking studies were conducted to validate that the presence of a peptidomimetic substituent on the backbone of the heptamethine cyanine fluorophores can increase the targeting potential of the fluorophores. To verify these data experimentally, the interaction between the fluorophores and the proteins can be studied by observing the fluorescence behavior of the fluorophores in the presence of these proteins. **TEA1–6** was found to have negligible fluorescence in aqueous HEPES and PBS buffers mainly due to the aggregation-induced quenching of fluorescence, and the increased vibrational relaxation due to the flexible structure and the large number of rotatable bonds.^65–68^ Therefore, it was hypothesized that the interaction between the proteins and the fluorophores could result in fluorescence enhancement of the latter because the rigid, protected environment of the protein’s binding pocket could restrict the intramolecular rotations and vibrations within the fluorophore, thereby minimizing nonradiative decay pathways.^85^ Furthermore, the protein structure could shield the probe from the solvent-induced quenching, further contributing to the enhanced emission.^33^

The fluorescence of the fluorophores (10 μM) was studied in the presence of increasing amounts of bovine serum albumin and human parvalbumin, and they were found to have enhanced fluorescence upon increasing the protein concentrations (from 0 to 200 μM). However, the behavior was also affected by time, as the fluorescence was observed to increase with the interaction time for the same protein concentration. For this reason, we decided to perform two studies: one is the fluorescence titration where the dyes’ fluorescence was measured at different protein concentrations, and the other is the kinetic analysis of the interaction between the proteins and the fluorophores to calculate the reaction rate constant and the half-life of the interaction based on pseudo-first-order reaction kinetics. For the kinetic analysis, the fluorescence of **TEA1–6** was observed over a period of 60 min in the presence of 100 μM of BSA or HPA. For **TEA1, 2, 4, and 5**, the fluorescence was enhanced and increased with time until reaching a plateau at different time intervals. By plotting the fluorescence intensity against time, the rate constant (K) and the reaction half-life (*T*_1/2_) could be calculated. Their average values for each fluorophore with both proteins are shown in Table 5.

**Table 5:** Kinetic Analysis Parameters of the Peptidomimetic Dyes Binding with BSA and HPA^a^

Dye	Parameter	Bovine Serum Albumin (BSA)	Human Parvalbumin (HPA)
**TEA1**	Rate constant K (s^–1^)	0.00252	0.00094
	Half-life *T*_1/2_ (s)	257.2	741.3
**TEA2**	Rate constant K (s^–1^)	0.00093	0.00042
	Half-life *T*_1/2_ (s)	748.8	1645
**TEA3**	Rate constant K (s^–1^)	-	-
	Half-life *T*_1/2_ (s)	-	-
**TEA4**	Rate constant K (s^–1^)	TFTM	TFTM
	Half-life *T*_1/2_ (s)	TFTM	TFTM
**TEA5**	Rate constant K (s^–1^)	0.00098	0.00049
	Half-life *T*_1/2_ (s)	710.8	1430
**TEA6**	Rate constant K (s^–1^)	-	-
	Half-life *T*_1/2_ (s)	-	-

a TFTM, Too Fast to Be Measured.

Interestingly, the fluorophores with the tryptamine moiety **TEA3** and **TEA6** did not show any fluorescence enhancement over time with both proteins, despite showing high affinity scores in the docking study. This can be attributed to the increased aggregation of these fluorophores in the aqueous buffer owing to the extra indole rings, which increased the pi–pi stacking, strengthened the aggregation, and made it difficult for the proteins to break it, and for the fluorophore molecules to enter inside the binding pockets of the proteins due to the increased steric hindrance.^86,87^ The aggregation became stronger by the extra aromatic system to the extent that it could compete with the protein binding, leading to the fluorophores leaning toward aggregation over binding, and this was the cause for the absence of fluorescence enhancement for these fluorophores.^88^

Looking at the values of the rate constant and the half-life of interactions of the different fluorophores with BSA and HPA in Table 5, it was also found that the fluorophores with the tyramine moieties (**TEA2 and TEA5**) had lower reaction rates compared to the fluorophores with the diethylamine moieties (**TEA1 and TEA4**) despite having larger binding affinity scores in the docking study. This could also be explained by the same reason for increased pi–pi stacking from the extra phenyl rings.^89^ However, the effect was not as pronounced as with the tryptamine-containing fluorophores due to the stronger pi–pi stacking of the indole in tryptamine compared to that of the phenyl group in tyramine.

The diethylamine-containing fluorophores (**TEA1 and TEA4**) were found to have the highest reaction rate constants with both BSA and HPA. Intriguingly, **TEA4** was very fast in its interaction that the rate constant and half-life could not be calculated from these experiments. This is due to the dual factors of the smaller alkyl chain of the carboxylic amide and the smaller diethyl groups compared to the tyramine or tryptamine moieties, which decreased the aggregation and facilitated the interaction with the proteins. The second fastest was **TEA1** with rate constants of 0.00251 and 0.00094 s^–1^ with BSA and HPA, respectively. The half-lives of interactions were 257.2 and 741.3 s for BSA and HPA, respectively. **TEA5** was faster than **TEA2** due to the shorter chain and the decrease in self-aggregation with reaction half-life values of 710.8 vs 748.8 s with BSA and 1430 vs 1645 s with HPA. Similar trends for the fluorophores’ interactions with BSA and HPA were observed as in the docking study, where the reaction rates were faster with BSA compared to HPA due to the better binding interactions and the bigger size of BSA compared to HPA. The rate constants and half-lives of the fluorophores’ interactions with the proteins are shown in Figure 13.

**Figure 13:**
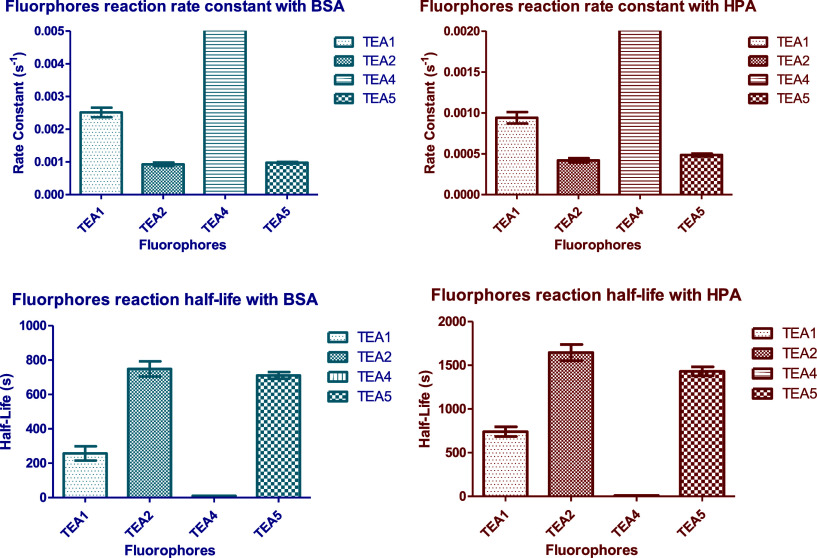
Bar charts showing the peptidomimetic dyes’ reaction rate constants (up) and half-lives (down) with BSA and HPA.

The fluorescence spectra of the different fluorophores in the presence of BSA and HPA, and the plots of fluorescence intensity against time are shown in Figures 14, 15, and S55–S62. Each fluorophore was tested three times with each protein, and the averages were calculated (Tables S1 and S2).

**Figure 14:**
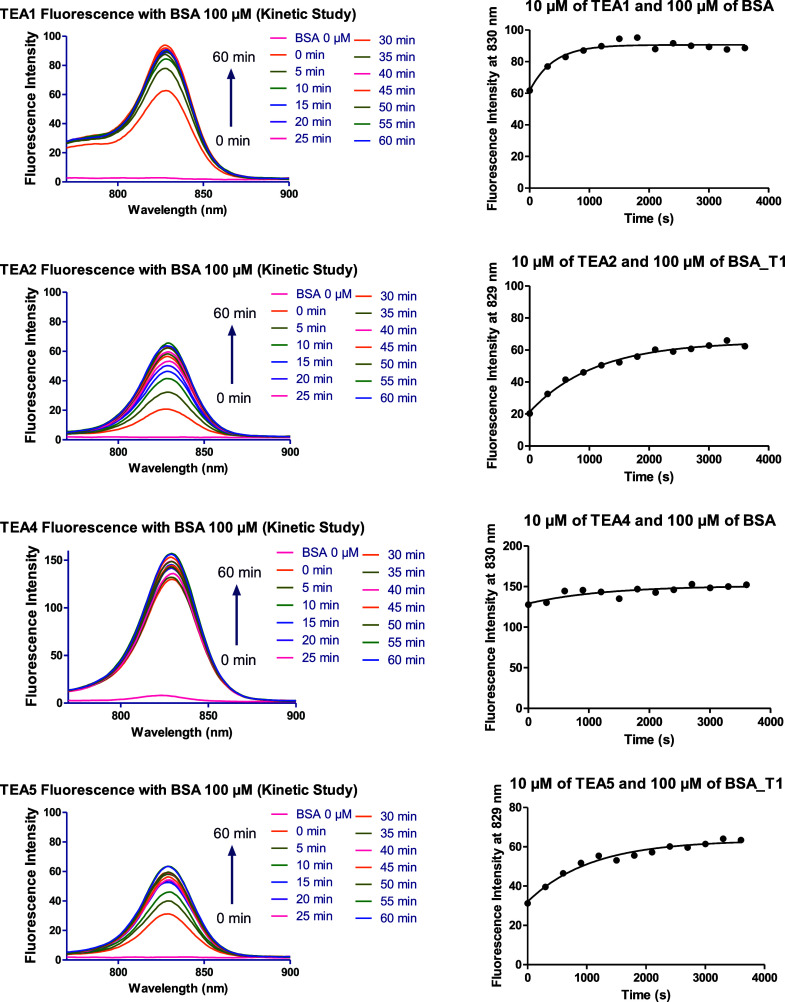
Fluorescence spectra of **TEA1, TEA2, TEA4, and TEA5** in PBS in the presence of 100 μM bovine serum albumin (BSA) during a time interval of 60 min and the corresponding plots of the change in fluorescence over time.

**Figure 15:**
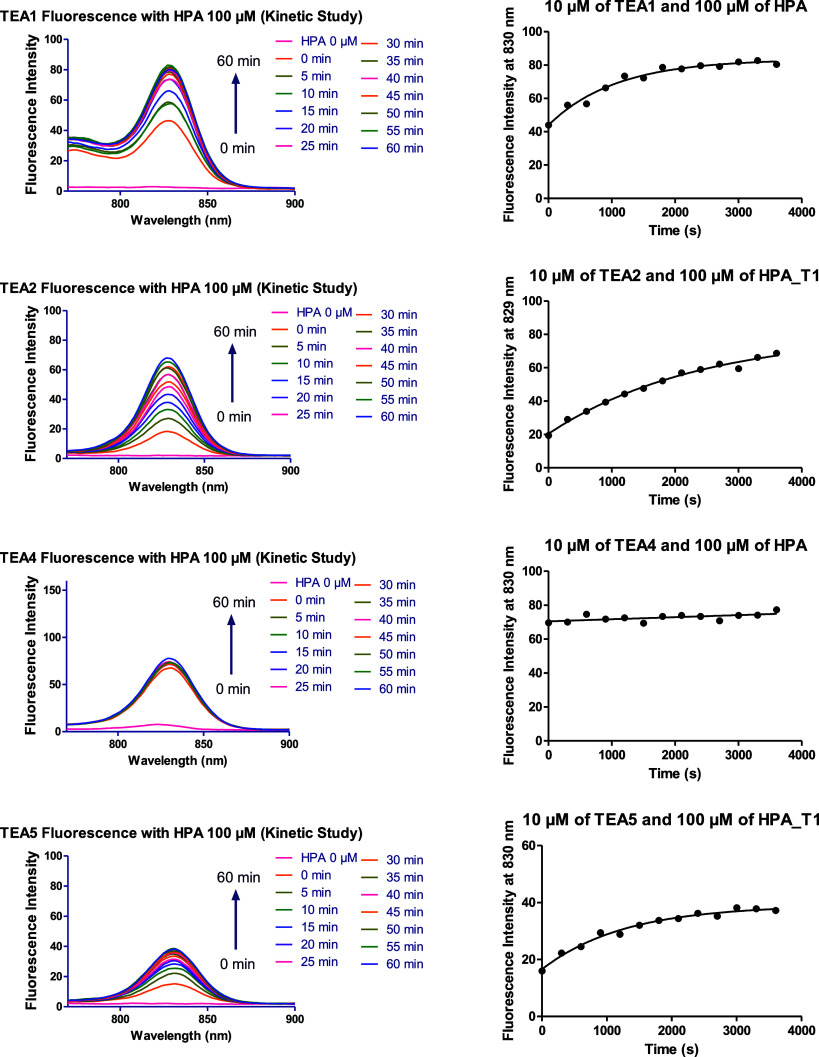
Fluorescence spectra of **TEA1, TEA2, TEA4, and TEA5** in PBS in the presence of 100 μM human parvalbumin (HPA) during a time interval of 60 min and the corresponding plots of the change in fluorescence over time.

Since the heptamethine cyanine fluorophores could exhibit a pH-dependent behavior, where their optical properties could change by changing the pH environment, it was crucial to conduct a fluorescence enhancement study at different physiological pH values to examine the effect of variable pH on the binding kinetics. **TEA1** was used as an example of the peptidomimetic dyes as it shows a moderate rate constant and half-life for binding to both BSA and HPA. The kinetic analysis was originally conducted for the peptidomimetic dyes at the physiological pH of 7.4, so the experiment was repeated for **TEA1** at four other pH values, spanning the physiological pH range from pH 2.0 to pH 9.5 (Table 6 and Figures S67 and S68) Interestingly, the rate constants and half-lives of interaction with both BSA and HPA showed comparable values at the different pH environments, which indicated that these peptidomimetic dyes were not pH-sensitive, and their optical properties did not change by changing the pH. This was expected as their conjugated system, which was responsible for their absorbance and fluorescence, did not contain any pH-sensitive moiety.

**Table 6:** Kinetic Analysis Parameters of **TEA1** Binding with BSA and HPA at Different pH Values

Dye	pH	Parameter	Bovine Serum Albumin (BSA)	Human Parvalbumin (HPA)
**TEA1**	2.0	Rate constant K (s^–1^)	0.00225	0.00099
		Half-life *T*_1/2_ (s)	307.8	696.6
	4.0	Rate constant K (s^–1^)	0.00286	0.00097
		Half-life *T*_1/2_ (s)	242.6	724.6
	6.0	Rate constant K (s^–1^)	0.00238	0.00098
		Half-life *T*_1/2_ (s)	290.7	703.4
	7.4	Rate constant K (s^–1^)	0.00252	0.00094
		Half-life *T*_1/2_ (s)	257.2	741.3
	9.5	Rate constant K (s^–1^)	0.00247	0.00091
		Half-life *T*_1/2_ (s)	280.1	763.2

Fluorescence titration experiments were conducted to examine the effect of increasing the concentration of the proteins on the fluorescence of the peptidomimetic dyes **TEA1, 2, 4, and 5** and to test the strength of binding between them. The fluorescence of 10 μM of the dyes was measured initially, and then it was measured again at increasing concentrations of each BSA and HPA (0–100 μM). Taking the effect of time on binding, each of these measurements was taken after 60 min to allow the binding to reach equilibrium (Figures 16 and 17 for BSA and HPA, respectively). As expected, the fluorescence intensity of the dyes showed enhancement with increasing concentration of each protein, reaching a plateau at around 80 μM of the protein. From the fluorescence intensity of the dyes at each protein concentration, the dissociation constant (*K*_d_) and binding constant could be calculated using the Benesi–Hildebrand plot (Figures S69 and S70), which plots the reciprocal of the change in fluorescence vs the reciprocal of the concentration of the protein. The binding constant or the association constant (Ka) was computed as well, from which the Gibbs free energy of binding (ΔG) was determined (Table 7), which gave an indication about the affinity of the peptidomimetic dyes to BSA or HPA. Interestingly, the binding constant and Gibbs free energy ranges followed the same trend as the kinetic parameters, where **TEA4** had the highest binding constant for both BSA and HPA (0.0142 and 0.0111 μM^–1^, respectively), which translated to the lowest ΔG (−5.66 and −5.52 kcal/mol for BSA and HPA, respectively), indicating that it had the highest affinity for both proteins.

**Figure 16:**
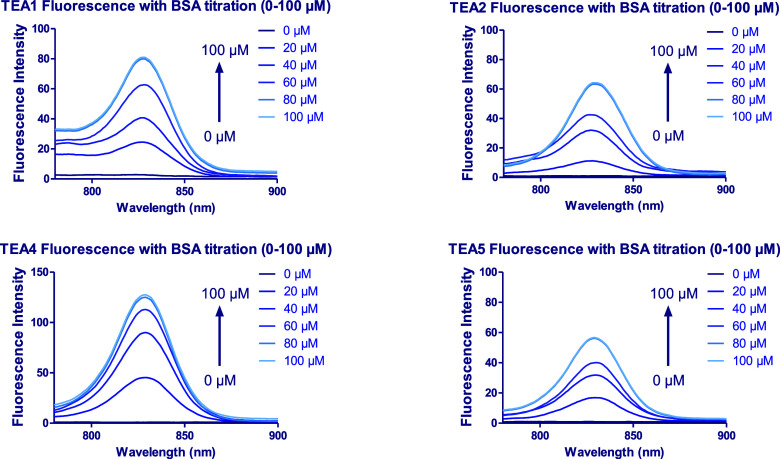
Fluorescence spectra of **TEA1, TEA2, TEA4, and TEA5** in PBS in the presence of increasing concentrations of bovine serum albumin (BSA) (0–100 μM).

**Figure 17:**
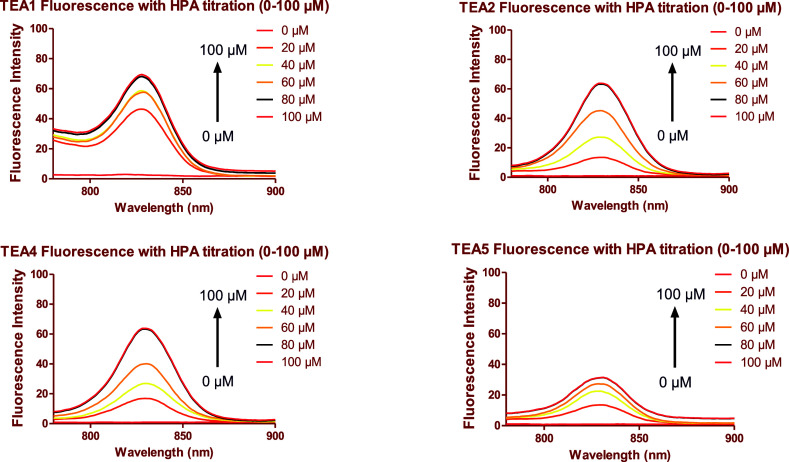
Fluorescence spectra of **TEA1, TEA2, TEA4, and TEA5** in PBS in the presence of increasing concentrations of human parvalbumin (HPA) (0–100 μM).

**Table 7:** Binding Affinity Parameters of the Peptidomimetic Dyes Binding with BSA and HPA

		Parameter		
Dye	Protein	*K*_d_ (μM)	Ka (μM^–1^)	ΔG (kcal/mol)
**TEA1**	BSA	82.23	0.0122	–5.57
	HPA	103.6	0.0097	–5.44
**TEA2**	BSA	214.5	0.0047	–5.00
	HPA	263.9	0.0038	–4.88
**TEA4**	BSA	70.49	0.0142	–5.66
	HPA	90.2	0.0111	–5.52
**TEA5**	BSA	100.2	0.0100	–5.46
	HPA	105.3	0.0095	–5.43

**TEA1** followed in the affinity, then **TEA5**, and finally **TEA2**. These results were not as predicted by the affinity or binding energies from the docking study, but they followed the same trend as the kinetic study. This discrepancy was, as mentioned, associated with the tendency of the fluorophores with the tyramine moiety (**TEA2** and **TEA5**) to aggregate more strongly by the pi–pi stacking in aqueous solutions, deviating from the affinity and ΔG energy expected results. Notably, the Gibbs free energy of binding of all the dyes for BSA was lower than that for HPA, aligning with the binding energies obtained from the docking studies.

Since the peptidomimetic dyes showed sensitivity toward BSA and HPA in the form of fluorescence enhancement upon binding, the fluorescence titration data were used to determine the dyes’ limit of detection (LOD) and limit of quantitation (LOQ) for BSA and HPA by plotting the fluorescence intensity of the dyes vs the concentration of the proteins (Figures S71 and S72). Remarkably, **TEA4** was the most sensitive to both proteins having the lowest LOD of 6.88 μM for BSA and 7.36 μM for HPA, followed by **TEA1**, with a calculated LOD of 8.22 and 9.10 μM for BSA and HPA, respectively. All the dyes had higher sensitivities toward BSA over HPA, manifested by a lower LOD and LOQ (Table 8), which matched the same trend shown in the binding affinity parameters.

**Table 8:** LOD and LOQ of the Peptidomimetic Dyes for BSA and HPA

		Parameter	
Dye	Protein	LOD (μM)	LOQ (μM)
**TEA1**	BSA	8.22	24.9
	HPA	9.10	27.6
**TEA2**	BSA	10.8	32.6
	HPA	11.6	35.1
**TEA4**	BSA	6.88	20.8
	HPA	7.36	22.3
**TEA5**	BSA	9.33	28.3
	HPA	10.1	30.6

After checking the sensitivity of the dyes to BSA and HPA, it was also important to test their selectivity. This was conducted by examining their fluorescence intensity in the presence of increasing concentrations of different physiological biomolecules. For these experiments, **TEA4** was used since it had the highest sensitivity and binding affinity toward the tested proteins. The experiment was conducted using various proteins or peptides, including collagen, tau protein, insulin fibrils, and lysozyme fibrils. Compellingly, **TEA4** did not show any fluorescence enhancement in the presence of these biomolecules even at higher concentrations, which indicated its selectivity toward BSA and HPA (Figures 18 “left” and S73).

**Figure 18:**
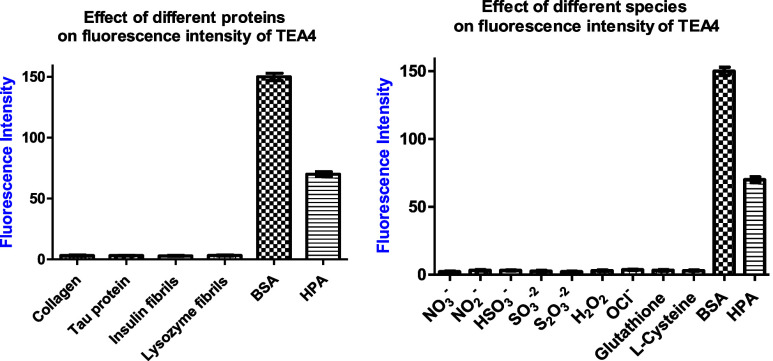
Fluorescence intensity of **TEA4** in PBS in the presence of different proteins, substrates, or ROS.

In addition to testing the effect of other proteins, the effect of common reactive species and ions present in the biological environment was also crucial to examine. Therefore, the fluorescence of **TEA4** was measured at increasing concentrations of some of the common biological small molecules, ions, or reactive oxygen species (ROS), namely: nitrate, nitrite, bisulfite, sulfite, thiosulfate, hydrogen peroxide, hypochlorite, glutathione, and l-cysteine (Figures 18 “right” and S74 and S75). Similar to the proteins/peptides, there was no fluorescence enhancement for **TEA4** in the presence of any of the tested species, ions, or ROS, which confirmed its selectivity toward BSA and HPA.

We were also interested in testing the competitive binding of the peptidomimetic dyes to BSA or HPA in the presence of competing proteins or species. That is why the BSA and HPA binding tests were repeated after the addition of different proteins or species. Remarkably, a similar fluorescence enhancement for **TEA4** was shown for each BSA or HPA addition, even in the presence of the competing proteins or species, indicating the high affinity of the dyes toward these proteins (Figure 19).

**Figure 19:**
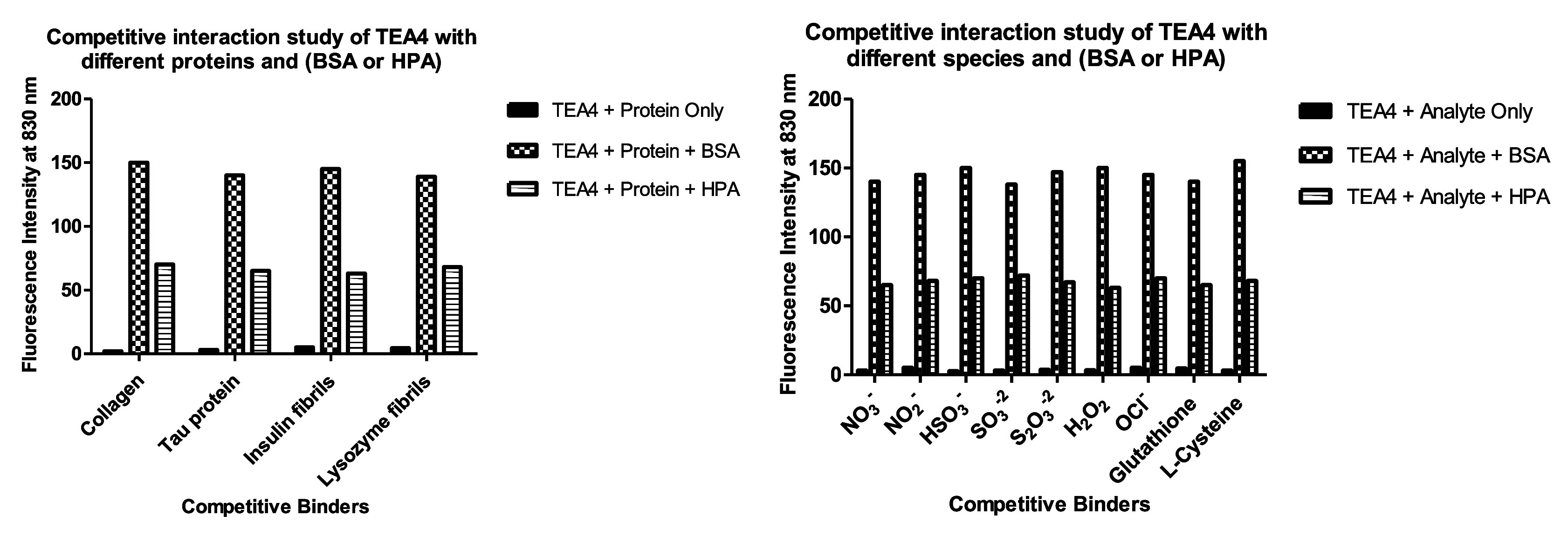
Fluorescence intensity of **TEA4** in PBS in a competitive binding study.

Cyanine dyes have the property of viscosity-induced fluorescence enhancement due to the fixation of the conjugated structure and inhibition of molecular rotation.^90–92^ For this reason, it was important to check if the fluorescence enhancement upon the addition of BSA or HPA was due to protein-specific binding or the increased microviscosity caused by the addition of the protein solutions. To accomplish this, the fluorescence of the peptidomimetic dyes **TEA1–6** was measured in water/glycerol solutions exhibiting increasing concentrations of glycerol to mimic the increase in viscosity (Figures 20 and S76). Fluorescence enhancement was observed for all of the peptidomimetic dyes with an increase in the ratio of glycerol in the solutions. Interestingly, **TEA3** and **TEA6**, possessing the tryptamide moieties, showed fluorescence enhancement upon increasing the glycerol concentration.

**Figure 20:**
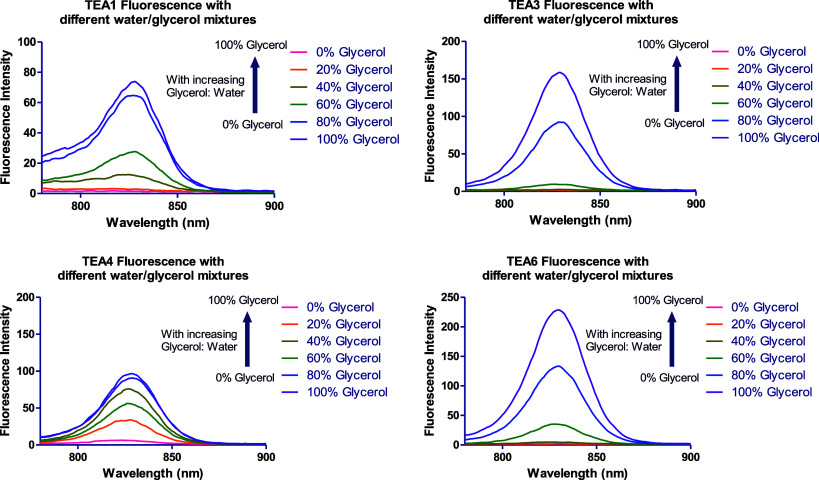
Fluorescence spectra of **TEA1, TEA3**, **TEA4**, and **TEA6** in solutions with increasing glycerol:water ratios.

**TEA3** and **TEA6** exhibited no fluorescence enhancement upon the addition of BSA or HPA, but their emission intensities did increase at higher glycerol ratios. This indicates that the fluorescence enhancement observed in increasing ratios of methanol or glycerol is not a consequence of increased solvent viscosity. Rather, it is driven by improved dye solubility and the subsequent disruption of molecular aggregates. These findings are consistent with the hydrophobicity profiles detailed earlier (Figures 1 and 2). A parallel trend was observed in water/methanol mixtures, where increasing the methanol fraction led to a nonspecific fluorescence enhancement across the entire panel of peptidomimetic dyes, including **TEA3** and **TEA6**.

To confirm these findings, absorption spectra for **TEA1–6** were acquired across a range of water/glycerol mixtures (Figures 21 and S77), mimicking the conditions of the earlier studies on hydrophobicity. The spectral changes induced by glycerol closely followed the trends observed with methanol. In purely aqueous solutions, the spectra possessed a broad absorption band with a blue-shifted H-aggregate peak. As the glycerol or methanol fractions increased, the intensity of this aggregate peak steadily declined, with a concomitant rise in the monomeric absorption peak.

**Figure 21:**
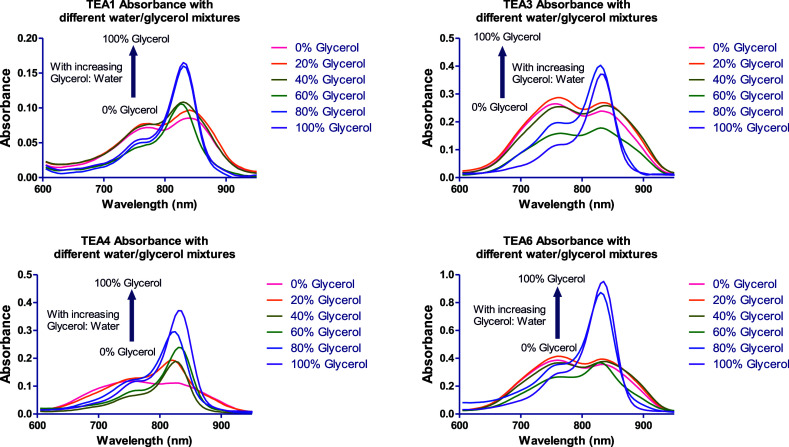
Absorbance spectra of **TEA1, TEA3**, **TEA4**, and **TEA6** in solutions with increasing glycerol:water ratios.

These results pointed out that the fluorescence enhancement upon BSA or HPA binding with **TEA1**, **TEA2**, **TEA4**, and **TEA5** was due to specific dye–protein interactions, and not because of the increased microviscosity of the solutions evidenced by the discrepancy between the effect of BSA or HPA addition, and the effect of increasing glycerol percentage in the water/glycerol mixtures. In contrast, **TEA3** and **TEA6** did not exhibit fluorescence enhancement with BSA or HPA addition; however, they showed increased fluorescence intensity upon increasing the glycerol percentage. In addition, the docking studies and the strong binding interactions between the dyes and the binding pockets of the two proteins further proved this hypothesis.

## Conclusion

This study reports the synthesis and evaluation of a series of amide-substituted heptamethine cyanine dyes (**TEA1–6**) designed with protein targeting in mind, where the peptide-like groups were introduced to encourage interactions with biological counterparts. The physicochemical properties of the dyes were studied in different solvents, where they showed strong absorbance in the NIR region. However, their behavior in aqueous-based buffers showed aggregation-based quenching of fluorescence because they were highly hydrophobic and tended to cluster into H-type aggregates, which effectively quenched their emission. Their peptidomimetic structures were evaluated for their targeting abilities, first by docking studies and then through experimental testing with two common biological proteins, namely BSA and HPA.

It was hypothesized that when these proteins were added, the aggregates could be broken apart, restoring fluorescence and confirming that binding served as a disaggregation trigger. However, not all substituents performed in the same way. Computational docking suggested strong binding across the entire series, yet the experimental evidence made it clear that aggregation often dictated the outcome. The tryptamine derivatives (**TEA3 and TEA6**) were especially prone to strong π–π stacking through their indole rings, creating aggregates that were too stable to allow for protein access or fluorescence recovery. In contrast, dyes with less bulky substituents, particularly **TEA4** with its diethylamine group, bonded to the protein more strongly and showed a faster fluorescence turn-on. Further sensitivity and selectivity experiments were conducted, showing the high sensitivity and selectivity of our synthesized dyes toward BSA and HPA.

*In vivo* studies of the biodistribution of these fluorophores and their interaction with BSA, HPA, and other proteins are warranted and are in the pipeline of this project. Overall, these results point to a balance that must be maintained in fluorophore design. Adding peptidomimetic character can improve recognition, but excessive aggregation counteracts this benefit. Future work on NIR probes should focus on tuning substituents so that the fluorophore remains accessible to proteins while still retaining biocompatibility.

## Experimental Section

### Materials and Methods

Chemicals used in the synthesis are American Chemical Society or HPLC grade, purchased from Sigma-Aldrich (Saint Louis, MO), Thermo Fisher Scientific, and TCI America (Waltham, MA). The ^1^HNMR (400 MHz) and ^13^CNMR (100 MHz) spectra were recorded by using a Bruker Avance spectrometer with DMSO-*d*_6_ (Cambridge Isotope Laboratories, Andover, MA), CDCl_3_ (Sigma-Aldrich, Burlington, MA), and MeOD (Sigma-Aldrich, Burlington, MA) containing tetramethylsilane (TMS) as an internal calibration standard. Chemical shifts are reported in parts per million (ppm). The following abbreviations are used for signal multiplicity: s (singlet), d (doublet), t (triplet), q (quartet), p (pentet), m (multiplet), and br (broad). Coupling constants (*J*) are provided in hertz (Hz). The melting points (mp) were measured with open Pyrex capillary tubes and a Thomas–Hoover apparatus. The absorbance and fluorescence properties were measured using a Varian Cary 50 spectrophotometer (Santa Clara, CA) and a Shimadzu RF-5301 PC spectrofluorometer, respectively. The VWR disposable two-sided polystyrene cuvettes with a path length of 1 cm were utilized to dissolve the dye in solvents for measurement. The quantum yields of dyes were measured according to the reported method with reference to indocyanine green (ICG).^93^ ESI-MS analyses were performed on a Waters Xevo G2_XS Mass Spectrometer (Waters Corporation, Milford, MA) equipped with an electrospray ionization source in positive ion mode. Each sample (5 μL) was introduced into the ion source through an autosampler with a 200 μL/min flow rate. The instrument operation parameters were optimized as follows: capillary voltage of 1000 V, sample cone voltage of 20 V, desolvation temperature of 350 °C, and source temperature of 120 °C. Nitrogen was used as cone gas and desolvation gas at pressures of 25 and 800 L/h, respectively. The spectra were acquired through a full scan analysis. MassLynx 4.2 software was used for data acquisition and processing. All ESI-MS spectra were acquired by the Mass Spectrometry Facility at the Georgia State University Department of Chemistry. The purity of all synthesized compounds was confirmed with ^1^HNMR, ^13^CNMR, and ESI-HRMS. These analytical methods were used to determine the purity of compounds to be ≥ 95%

## Chemistry

### Synthesis of the benz­[*e*]­indolium Salts (**C1,2**)

The benz­[*e*]­indolium salts (**C1,2**) were prepared according to a previous literature method.^94^ 1,1,2-Trimethyl-1H-benz­[*e*]­indole 3 (9.6 mmol, 1 equiv) was dissolved in 1,2-dichlorobenzene (5 mL), then 6-bromohexanoic acid or 3-bromopropanoic acid (3 equiv) was added. The reaction mixture was heated at 90 °C for 24 h; then it was cooled to room temperature to allow the product to precipitate. The solid was filtered, washed with 1,2-dichlorobenzene and ether, and then left to dry to obtain the benz­[*e*]­indolium salts **C1,2** as violet solids.

#### 3-(5-Carboxypentyl)-1,1,2-trimethyl-1H-benzo­[*e*]­indol-3-ium Bromide (**C1**)

Yield (98%, 3.8 g); ^1^H NMR (400 MHz, DMSO-*d*_6_) δ 12.03 (s, 1H), 8.38 (d, *J* = 8.3 Hz, 1H), 8.30 (d, *J* = 8.9 Hz, 1H), 8.23 (d, *J* = 8.3 Hz, 1H), 8.18 (d, *J* = 8.9 Hz, 1H), 7.79 (t, *J* = 7.2 Hz, 1H), 7.73 (t, *J* = 7.2 Hz, 1H), 4.60 (t, *J* = 7.7 Hz, 2H), 2.97 (s, 3H), 2.24 (t, *J* = 7.7 Hz, 2H), 1.91 (p, *J* = 7.7 Hz, 2H), 1.77 (s, 6H), 1.58 (p, *J* = 7.7 Hz, 2H), 1.47 (p, *J* = 7.7 Hz, 2H).

#### 3-(2-Carboxyethyl)-1,1,2-trimethyl-1H-benzo­[*e*]­indol-3-ium Bromide (**C2**)

Yield (91%, 3.1 g); ^1^H NMR (400 MHz, DMSO-*d*_6_) δ 12.75 (s, 1H), 8.38 (d, *J* = 8.3 Hz, 1H), 8.29 (d, *J* = 9.0 Hz, 1H), 8.22 (d, *J* = 8.3 Hz, 1H), 8.19 (d, *J* = 9.0 Hz, 1H), 7.79 (t, *J* = 6.9 Hz, 1H), 7.73 (t, *J* = 6.9 Hz, 1H), 4.79 (t, *J* = 7.0 Hz, 2H), 3.06 (t, *J* = 7.0 Hz, 2H), 2.98 (s, 3H), 1.76 (s, 6H).

### Synthesis of the Amide-Substituted Benz­[*e*]­indolium Salts (**D1–6**)

The amide coupling was done by reacting carboxylic-acid-substituted benz­[*e*]­indolium salts **C1,2** with different primary or secondary amines in the presence of hexafluorophosphate benzotriazole tetramethyl uronium (HBTU) as a coupling reagent. The benz­[*e*]­indolium salt **C1,2** (1 equiv) was dissolved in DCM or DCM/acetonitrile (1:1), and then HBTU (1.3 equiv) was added. This mixture was stirred at room temperature for 1 h, and then the amine (2 equiv) dissolved in THF was added dropwise to the reaction. The reaction mixture was allowed to stir at room temperature overnight, then the solvent was evaporated, and the residue was redissolved in a mixture of DCM/0.1 N HCl. This mixture was extracted 2 more times with DCM and then concentrated to obtain a residue which was purified by silica gel column chromatography using DCM/methanol (97:3). The product fractions were collected and concentrated to obtain a violet residue, which was either used as is or recrystallized using methanol/diethyl ether to obtain the amide-substituted salts **D1–6** as a violet powder.

#### 3-(6-(Diethylamino)-6-oxohexyl)-1,1,2-trimethyl-1H-benzo­[*e*]­indol-3-Ium Bromide (**D1**)

Isolated by silica gel column chromatography using DCM/MeOH 97:3 and recrystallization using methanol/diethyl ether to give a violet solid. Yield (21%, 0.12 g); mp 180–182 °C; ^1^H NMR (400 MHz, DMSO-*d*_6_) δ 8.38 (d, *J* = 8.1 Hz, 1H), 8.30 (d, *J* = 8.9 Hz, 1H), 8.23 (d, *J* = 8.1 Hz, 1H), 8.15 (d, *J* = 8.9 Hz, 1H), 7.80 (t, *J* = 7.6 Hz, 1H), 7.74 (t, *J* = 7.6 Hz, 1H), 4.59 (t, *J* = 7.3 Hz, 2H), 3.24 (dq, *J* = 14.4, 7.1 Hz, 4H), 2.95 (s, 3H), 2.28 (t, *J* = 7.3 Hz, 2H), 1.92 (p, *J* = 7.3 Hz, 2H), 1.77 (s, 6H), 1.58 (p, *J* = 7.3 Hz, 2H), 1.45 (p, *J* = 7.3 Hz, 2H), 1.06 (t, *J* = 7.0 Hz, 3H), 0.95 (t, *J* = 7.0 Hz, 3H). ^13^C NMR (101 MHz, DMSO-*d*_6_) δ: 196.4, 170.7, 138.5, 137.0, 133.1, 130.7, 129.7, 128.4, 127.3, 127.3, 123.4, 113.3, 55.5, 47.7, 41.2, 31.7, 27.3, 25.6, 24.5, 21.6, 14.3, 13.7, 13.1.

#### 3-(6-((4-Hydroxyphenethyl)­amino)-6-oxohexyl)-1,1,2-trimethyl-1H-benzo­[*e*]­indol-3-ium Bromide (D2)

Isolated by silica gel column chromatography using DCM/MeOH 97:3 and recrystallization using methanol/diethyl ether to get a violet solid. Yield (93%, 0.60 g); mp 185–186 °C; ^1^H NMR (400 MHz, DMSO-*d*_6_) δ 9.18 (s, 1H), 8.38 (d, *J* = 8.2 Hz, 1H), 8.29 (d, *J* = 8.9 Hz, 1H), 8.22 (d, *J* = 8.2 Hz, 1H), 8.14 (d, *J* = 8.9 Hz, 1H), 7.83 (t, *J* = 4.4 Hz, 1H), 7.79 (s, 1H), 7.73 (t, *J* = 4.4 Hz, 1H), 6.94 (d, *J* = 8.0 Hz, 2H), 6.66 (d, *J* = 8.0 Hz, 2H), 4.57 (t, *J* = 7.6 Hz, 2H), 3.15 (q, *J* = 6.9 Hz, 2H), 2.94 (s, 3H), 2.54 (br t, 2H), 2.06 (t, *J* = 7.6 Hz, 2H), 1.89 (p, *J* = 7.6 Hz, 2H), 1.76 (s, 6H), 1.56 (p, *J* = 7.6 Hz, 2H), 1.40 (p, *J* = 7.6 Hz, 2H). ^13^C NMR (101 MHz, DMSO-*d*_6_) δ: 196.4, 171.7, 155.6, 138.5, 137.0, 133.1, 130.7, 129.7, 129.4, 128.4, 127.3, 123.4, 115.1, 113.3, 55.5, 54.9, 47.7, 35.0, 34.4, 27.2, 25.5, 24.8, 21.6, 13.7.

#### 3-(6-((2-(1H-Indol-3-yl)­ethyl)­amino)-6-oxohexyl)-1,1,2-trimethyl-1H-benzo­[*e*]­indol-3-ium Bromide (**D3**)

Isolated by silica gel column chromatography using DCM/MeOH 97:3 and recrystallization using methanol/diethyl ether to get a violet solid. Yield (89%, 0.60 g); mp 175–177 °C; ^1^H NMR (400 MHz, DMSO-*d*_6_) δ 10.77 (d, *J* = 20.8 Hz, 1H), 8.37 (d, *J* = 8.1 Hz, 1H), 8.28 (d, *J* = 8.9 Hz, 1H), 8.21 (d, *J* = 8.1 Hz, 1H), 8.14 (d, *J* = 8.9 Hz, 1H), 7.91 (s, 1H), 7.79 (t, *J* = 7.6 Hz, 1H), 7.73 (t, *J* = 7.6 Hz, 1H), 7.51 (d, *J* = 7.5 Hz, 1H), 7.34 (d, *J* = 7.5 Hz, 1H), 7.11 (s, 1H), 7.06 (t, *J* = 7.5 Hz, 1H), 6.97 (t, *J* = 7.5 Hz, 1H), 4.57 (t, *J* = 7.6 Hz, 2H), 3.40 (br t, 2H), 2.93 (s, 3H), 2.78 (t, *J* = 7.3 Hz, 2H), 2.10 (t, *J* = 7.6 Hz, 2H), 1.98–1.83 (m, 2H), 1.76 (s, 6H), 1.61–1.55 (m, 2H), 1.46–1.40 (m, 2H). ^13^C NMR (101 MHz, DMSO-*d*_6_) δ 196.4, 171.8, 138.5, 137.0, 136.2, 133.1, 130.7, 129.7, 128.4, 127.3, 127.2, 123.4, 122.6, 120.9, 118.2, 118.2, 113.3, 111.9, 111.4, 55.5, 54.9, 47.7, 35.1, 27.2, 25.5, 25.3, 24.7, 21.6, 13.7.

#### 3-(3-(Diethylamino)-3-oxopropyl)-1,1,2-trimethyl-1H-benzo­[*e*]­indol-3-ium Bromide (**D4**)

The residue after the column chromatography was used as is in the next step.

#### 3-(3-((4-Hydroxyphenethyl)­amino)-3-oxopropyl)-1,1,2-trimethyl-1H-benzo­[*e*]­indol-3-ium Bromide (**D5**)

The residue after the column chromatography was used as is in the next step.

#### 3-(3-((2-(1H-Indol-3-yl)­ethyl)­amino)-3-oxopropyl)-1,1,2-trimethyl-1H-benzo­[*e*]­indol-3-ium Bromide (**D6**)

Isolated by silica gel column chromatography using DCM/MeOH 97:3 and recrystallization using methanol/diethyl ether to get a violet solid. Yield (61%, 0.51 g); mp 170–172 °C; ^1^H NMR (400 MHz, DMSO-*d*_6_) δ 10.80 (s, 1H), 8.38 (d, *J* = 8.3 Hz, 1H), 8.30 (d, *J* = 8.9 Hz, 1H), 8.27 (s, 1H), 8.22 (d, *J* = 8.3 Hz, 1H), 8.12 (d, *J* = 8.9 Hz, 1H), 7.80 (t, *J* = 7.3 Hz, 1H), 7.74 (t, *J* = 7.3 Hz, 1H), 7.36 (d, *J* = 7.9 Hz, 1H), 7.30 (d, *J* = 7.9 Hz, 1H), 7.06 (s, 1H), 7.03 (t, *J* = 7.9 Hz, 1H), 6.90 (t, *J* = 7.9 Hz, 1H), 4.81 (t, *J* = 6.5 Hz, 2H), 3.26 (q, *J* = 6.9 Hz, 2H), 2.91 (s, 3H), 2.85 (t, *J* = 6.9 Hz, 2H), 2.65 (t, *J* = 6.5 Hz, 2H), 1.74 (s, 6H). ^13^C NMR (101 MHz, DMSO-*d*_6_) δ: 197.5, 168.5, 138.3, 136.8, 136.2, 133.0, 130.6, 129.7, 128.4, 127.3, 127.1, 123.4, 122.6, 120.9, 118.2, 118.0, 113.4, 111.5, 111.4, 55.6, 54.9, 44.6, 32.8, 24.9, 21.4, 14.0.

### Synthesis of the Vilsmeier Linker (**F**)

The linker for heptamethine cyanine dye **F** was obtained through the Vilsmeier–Haack chloroformylation. Dimethylformamide (18 mL, 229 mmol, 4.5 equiv) was cooled to 0 °C in an ice bath. Phosphorus oxychloride POCl_3_ (11 mL, 115 mmol, 2.3 equiv) was added dropwise to the DMF solution while in the ice bath and stirred at 0 °C for 30 min. Cyclohexanone **E** (5.3 mL, 51 mmol, 1 equiv) was added while in the ice bath, and then the reaction mixture was heated at 70 °C for 4 h. The reaction mixture was poured onto an ice/water mixture (500 mL) and stirred at room temperature overnight. The formed precipitate was filtered, washed with distilled water, and dried in vacuo to obtain the product as a yellow solid which was used in the following step without further purification (5.6 g, 64%).

### Synthesis of the Heptamethine Cyanine Dyes (**TEA1–6**)

The heptamethine cyanine dyes were synthesized as shown in Scheme 1, by reacting benz­[*e*]­indolium salts **D1–6** with the Vilsmeier linker **F** in the presence of sodium acetate as a base. The benz­[*e*]­indolium salt (2 equiv) were mixed with the Vilsmeier linker (1 equiv) and sodium acetate (2 equiv) in acetic anhydride (7 mL). The reaction mixture was heated to 70 °C for 3–5 h according to the indole salt used. The reaction was followed using TLC and vis-NIR spectroscopy. After the completion of the reaction, diethyl ether (100 mL) was added to precipitate the formed heptamethine dye. The solid was then filtered and washed with distilled water to remove the excess sodium acetate. The products were purified by recrystallization by dissolving them in the least amount of methanol (1–2 mL) and then precipitating with diethyl ether or THF (100 mL) to obtain the heptamethine dyes **TEA1–6** by filtration as green solids.

#### 2-((E)-2-((E)-2-Chloro-3-((E)-2-(3-(6-(diethylamino)-6-oxohexyl)-1,1-dimethyl-1,3-dihydro-2H-benzo­[*e*]­indol-2-ylidene)­ethylidene)­cyclohex-1-en-1-yl)­vinyl)-3-(6-(diethylamino)-6-oxohexyl)-1,1-dimethyl-1H-benzo­[*e*]­indol-3-ium Bromide (**TEA1**)

Yield (81%, 0.08 g); mp 191–193 °C; ^1^H NMR (400 MHz, MeOD) δ 8.43 (d, *J* = 14.1 Hz, 2H), 8.16 (d, *J* = 8.6 Hz, 2H), 7.91 (dd, *J* = 13.8, 8.6 Hz, 4H), 7.59 – 7.50 (m, 4H), 7.39 (t, *J* = 7.6 Hz, 2H), 6.23 (d, *J* = 14.1 Hz, 2H), 4.21 (t, *J* = 7.3 Hz, 4H), 3.24 (q, *J* = 7.1 Hz, 8H), 2.66 (t, *J* = 6.0 Hz, 4H), 2.28 (t, *J* = 7.3 Hz, 4H), 1.91 (s, 12H), 1.85 (br p, 2H), 1.81 (br p, 4H), 1.61 (p, *J* = 7.3 Hz, 4H), 1.42 (p, *J* = 7.3 Hz, 4H), 1.03 (t, *J* = 7.1 Hz, 6H), 0.94 (t, *J* = 7.1 Hz, 6H). ^13^C NMR (101 MHz, MeOD) δ 175.7, 174.5, 150.6, 144.6, 141.3, 135.4, 133.7, 132.0, 131.3, 129.6, 129.0, 128.1, 126.4, 123.6, 112.4, 102.2, 52.6, 45.5, 43.6, 41.7, 33.6, 28.8, 28.1, 27.7, 27.6, 26.4, 14.7, 13.4; HRMS (ESI) Calcd for [C_58_H_74_N_4_O_2_Cl]^+^ *m*/*z* 893.5500, found *m*/*z* 893.5521; λ_abs_ = 824 nm in EtOH.

#### 3-(6-((4-Acetoxyphenethyl)­amino)-6-oxohexyl)-2-((E)-2-((E)-3-((E)-2-(3-(6-((4-acetoxyphenethyl)­amino)-6-oxohexyl)-1,1-dimethyl-1,3-dihydro-2H-benzo­[*e*]­indol-2-ylidene)­ethylidene)-2-chlorocyclohex-1-en-1-yl)­vinyl)-1,1-dimethyl-1H-benzo­[*e*]­indol-3-ium Bromide (**TEA2**)

Yield (66%, 0.27 g); mp 196–198 °C; ^1^H NMR (400 MHz, MeOD/DMSO) δ 8.56 (d, *J* = 14.2 Hz, 2H), 8.36 (d, *J* = 8.6 Hz, 2H), 8.13 (dd, *J* = 12.9, 8.6 Hz, 4H), 7.74 (t, *J* = 7.6 Hz, 4H), 7.60 (t, *J* = 7.6 Hz, 2H), 7.25 (d, *J* = 8.4 Hz, 4H), 7.07 (d, *J* = 8.4 Hz, 4H), 6.42 (d, *J* = 14.2 Hz, 2H), 4.39 (t, *J* = 7.3 Hz, 4H), 3.37 (t, *J* = 7.4 Hz, 4H), 2.83 (t, *J* = 6.1 Hz, 4H), 2.76 (t, *J* = 7.4 Hz, 4H), 2.29 (s, 6H), 2.23 (t, *J* = 7.3 Hz, 4H), 2.09 (s, 12H), 2.04–1.99 (m, 2H), 1.95 (p, *J* = 7.3 Hz, 4H), 1.73 (p, *J* = 7.4 Hz, 4H), 1.52 (p, *J* = 7.3 Hz, 4H). ^13^C NMR (101 MHz, MeOD/DMSO) δ 175.5, 175.0, 171.0, 150.8, 150.1, 144.3, 141.3, 138.4, 135.4, 133.5, 132.1, 131.4, 131.0, 129.4, 129.2, 128.0, 126.6, 123.7, 123.0, 116.5, 112.8, 102.4, 52.5, 45.5, 41.8, 36.7, 36.1, 28.6, 28.2, 27.5, 27.4, 26.6, 22.3, 21.5; HRMS (ESI) Calcd for [C_70_H_78_N_4_O_6_Cl]^+^ *m*/*z* 1105.5610, found *m*/*z* 1105.5635; λ_abs_ = 826 nm in EtOH.

#### 3-(6-((2-(1H-Indol-3-yl)­ethyl)­amino)-6-oxohexyl)-2-((E)-2-((E)-3-((E)-2-(3-(6-((2-(1H-indol-3-yl)­ethyl)­amino)-6-oxohexyl)-1,1-dimethyl-1,3-dihydro-2H-benzo­[*e*]­indol-2-ylidene)­ethylidene)-2-chlorocyclohex-1-en-1-yl)­vinyl)-1,1-dimethyl-1H-benzo­[*e*]­indol-3-ium Bromide (**TEA3**)

Yield (79%, 0.33 g); mp 205–207 °C; ^1^H NMR (400 MHz, MeOD/DMSO) δ 8.54 (d, *J* = 14.1 Hz, 2H), 8.35 (d, *J* = 8.5 Hz, 2H), 8.11 (t, *J* = 8.5 Hz, 4H), 7.74 (t, *J* = 7.4 Hz, 4H), 7.60 (q, *J* = 8.1 Hz, 4H), 7.41 (d, *J* = 8.1 Hz, 2H), 7.14 (t, *J* = 7.4 Hz, 4H), 7.06 (t, *J* = 7.4 Hz, 2H), 6.38 (d, *J* = 14.1 Hz, 2H), 4.35 (t, *J* = 7.6 Hz, 4H), 3.46 (t, *J* = 7.5 Hz, 4H), 2.92 (t, *J* = 7.5 Hz, 4H), 2.78 (br t, 4H), 2.25 (t, *J* = 7.6 Hz, 4H), 2.08 (s, 12H), 1.99 (br p, 2H), 1.95 (p, *J* = 7.6 Hz, 4H), 1.75 (p, *J* = 7.6 Hz, 4H), 1.54 (p, *J* = 7.6 Hz, 4H). ^13^C NMR (101 MHz, MeOD/DMSO) δ 175.4, 175.0, 144.3, 141.3, 138.0, 135.4, 133.5, 132.1, 131.4, 129.4, 129.2, 129.0, 128.0, 126.6, 123.8, 123.7, 122.6, 119.9, 119.7, 113.6, 112.8, 112.7, 102.4, 66.8, 52.5, 45.5, 41.3, 36.8, 28.6, 28.2, 27.4, 26.6, 26.6, 16.0; HRMS (ESI) Calcd for [C_70_H_76_N_6_O_2_Cl]^+^ *m*/*z* 1067.5718, found *m*/*z* 1067.5736; λ_abs_ = 828 nm in EtOH.

#### 2-((E)-2-((E)-2-Chloro-3-((E)-2-(3-(3-(diethylamino)-3-oxopropyl)-1,1-dimethyl-1,3-dihydro-2H-benzo­[*e*]­indol-2-ylidene)­ethylidene)­cyclohex-1-en-1-yl)­vinyl)-3-(3-(diethylamino)-3-oxopropyl)-1,1-dimethyl-1H-benzo­[*e*]­indol-3-ium Bromide (**TEA4**)

Yield (73%, 0.39 g); mp 178–180 °C; ^1^H NMR (400 MHz, MeOD) δ 8.46 (d, *J* = 14.1 Hz, 2H), 8.19 (d, *J* = 8.6 Hz, 2H), 7.94 (dd, *J* = 16.1, 8.6 Hz, 4H), 7.60–7.55 (m, 4H), 7.42 (t, *J* = 8.6 Hz, 2H), 6.38 (d, *J* = 14.1 Hz, 2H), 4.55 (t, *J* = 6.6 Hz, 4H), 3.28 (q, *J* = 7.2 Hz, 8H), 2.91 (t, *J* = 6.6 Hz, 4H), 2.72 (t, *J* = 6.2 Hz, 4H), 1.94 (s, 12H), 1.88 (p, *J* = 6.2 Hz, 2H), 1.02 (t, *J* = 7.2 Hz, 6H), 0.97 (t, *J* = 7.2 Hz, 6H). ^13^C NMR (101 MHz, MeOD) δ 175.7, 171.3, 150.8, 144.8, 141.0, 135.4, 133.7, 132.1, 131.3, 129.5, 129.0, 128.7, 126.4, 123.6, 112.4, 102.7, 52.6, 43.9, 42.4, 42.1, 31.7, 28.1, 27.6, 14.7, 13.4; HRMS (ESI) Calcd for [C_52_H_62_N_4_O_2_Cl]^+^ *m*/*z* 809.4561, found *m*/*z* 809.4547; λ_abs_ = 826 nm in EtOH.

#### 3-(3-((4-Acetoxyphenethyl)­amino)-3-oxopropyl)-2-((E)-2-((E)-3-((E)-2-(3-(3-((4-acetoxyphenethyl)­amino)-3-oxopropyl)-1,1-dimethyl-1,3-dihydro-2H-benzo­[*e*]­indol-2-ylidene)­ethylidene)-2-chlorocyclohex-1-en-1-yl)­vinyl)-1,1-dimethyl-1H-benzo­[*e*]­indol-3-ium Bromide (**TEA5**)

Yield (51%, 0.25 g); mp 185–187 °C; ^1^H NMR (400 MHz, MeOD) δ 8.55 (d, *J* = 14.1 Hz, 2H), 8.29 (d, *J* = 8.6 Hz, 2H), 8.05 (dd, *J* = 13.2, 8.6 Hz, 4H), 7.76–7.61 (m, 4H), 7.53 (t, *J* = 8.6 Hz, 2H), 7.03 (d, *J* = 8.2 Hz, 4H), 6.85 (d, *J* = 8.2 Hz, 4H), 6.46 (d, *J* = 14.1 Hz, 2H), 4.59 (t, *J* = 6.4 Hz, 4H), 3.35 (t, *J* = 7.3 Hz, 4H), 2.81 (t, *J* = 6.4 Hz, 4H), 2.74 (t, *J* = 6.2 Hz, 4H), 2.64 (t, *J* = 7.3 Hz, 4H), 2.21 (s, 6H), 2.02 (s, 12H), 1.98 (br p, 2H). ^13^C NMR (101 MHz, MeOD) δ: 175.8, 172.6, 171.4, 150.9, 144.9, 140.9, 138.0, 135.5, 133.8, 132.0, 131.3, 131.0, 130.6, 129.6, 129.0, 128.7, 126.5, 123.6, 123.4, 122.8, 112.5, 102.9, 52.6, 42.3, 42.0, 35.7, 35.3, 28.1, 27.7, 22.4, 21.0. HRMS (ESI) Calcd for [C_64_H_66_N_4_O_6_Cl]^+^ *m*/*z* 1021.4671, found *m*/*z* 1021.4670; λ_abs_ = 830 nm in EtOH.

#### 3-(3-((2-(1H-Indol-3-yl)­ethyl)­amino)-3-oxopropyl)-2-((E)-2-((E)-3-((E)-2-(3-(3-((2-(1H-indol-3-yl)­ethyl)­amino)-3-oxopropyl)-1,1-dimethyl-1,3-dihydro-2H-benzo­[*e*]­indol-2-ylidene)­ethylidene)-2-chlorocyclohex-1-en-1-yl)­vinyl)-1,1-dimethyl-1H-benzo­[*e*]­indol-3-ium Bromide (**TEA6**)

Yield (59%, 0.25 g); mp 193–195 °C; ^1^H NMR (400 MHz, MeOD/DMSO) δ 8.55 (d, *J* = 14.1 Hz, 2H), 8.34 (d, *J* = 8.6 Hz, 2H), 8.10 (t, *J* = 8.6 Hz, 4H), 7.79–7.69 (m, 4H), 7.58 (t, *J* = 8.6 Hz, 2H), 7.43 (d, *J* = 7.8 Hz, 2H), 7.35 (d, *J* = 7.8 Hz, 2H), 7.10 (t, *J* = 7.8 Hz, 2H), 7.05–6.94 (m, 4H), 6.53 (d, *J* = 14.1 Hz, 2H), 4.65 (t, *J* = 5.9 Hz, 4H), 3.42 (t, *J* = 7.5 Hz, 4H), 2.83 (t, *J* = 5.9 Hz, 4H), 2.76 (t, *J* = 7.5 Hz, 4H), 2.65 (t, *J* = 6.3 Hz, 4H), 2.06 (s, 12H), 1.96 (p, *J* = 6.3 Hz, 2H). ^13^C NMR (101 MHz, MeOD/DMSO) δ: 175.6, 172.0, 150.4, 144.5, 141.1, 138.0, 135.4, 133.5, 131.9, 131.4, 129.4, 129.1, 128.8, 128.5, 126.5, 123.7, 123.5, 122.6, 119.9, 119.5, 113.3, 112.8, 112.6, 103.0, 66.8, 52.5, 42.5, 41.4, 35.4, 28.3, 26.4, 15.9. HRMS (ESI) Calcd for [C_64_H_64_N_6_O_2_Cl]^+^ *m*/*z* 983.4779, found *m*/*z* 983.4783; λ_abs_ = 830 nm in EtOH.

### Optical Studies (Photophysical Studies)

One mM stock solution of each synthesized fluorophore was prepared in DMSO prior to all spectral measurements. The optical properties of these fluorophores were investigated in four solvents: ethanol (EtOH), dimethyl sulfoxide (DMSO), HEPES buffer, and phosphate-buffered saline (PBS). Absorbance spectra were acquired by using a Varian Cary 50 spectrophotometer (190–1100 nm). Fluorescence emission spectra were measured on a Shimadzu RF-5301PC spectrofluorometer.

### Quantum Yield of Fluorescence (Φ_f_) Calculation

The quantum yield of the synthesized fluorophores was calculated by measuring their fluorescence intensity using a Shimadzu RF-5301PC spectrofluorometer and comparing it to that of the standard FDA-approved fluorophore indocyanine green (ICG) because it had a similar heptamethine cyanine structure and a comparable fluorescence wavelength (802 nm in HEPES buffer vs 824 nm for **TEA1** as an example). The values of ICG quantum yield were retrieved from the literature, and they were 14% in ethanol,^62^ 16.7% in DMSO,^55^ and 2.9% in HEPES and PBS buffers since they were water-based buffers.^62^ The quantum yield of the synthesized fluorophores was calculated in the four different solvents used, namely: ethanol, DMSO, HEPES buffer, and PBS buffer. The fluorophore concentrations varied between 4 and 8 μM. The excitation wavelengths used were 750 or 755 nm, and the excitation and emission slit widths were both 5 nm.

### Photothermal Stability Studies

Solutions of the synthesized fluorophores were prepared in ethanol at a concentration of 4–11 μM and placed in closed microwave vials. Two sets were prepared: the first set was put in dark conditions by covering them with foil plates and keeping them in a dark drawer at room temperature (25 °C), and the second set was continuously irradiated with the light of a 6000 mW 254 nm UV lamp placed 10 cm away from the dye solutions. This second set was covered to prevent any other light from interfering and kept at room temperature (25 °C). Samples for each fluorophore were taken from both sets and their absorbance measured at 0, 24, 48, and 72 h; then the samples were returned to the vials and stored for the next round of measurements.

### Density-Functional Theory (DFT) Study

The Density-Functional Theory (DFT) study was conducted using Spartan software,^71^ where the molecules were imported into the software as mol. files, autoconverted from 2D to 3D structures, and then initial energy minimization was done using “Equilibrium Geometry” calculations at the ground state in the gas phase using the ″Molecular Mechanics MMFF” algorithm. After initial energy minimization, the Density-Functional Theory (DFT) energy minimization and calculation was done using “Equilibrium Geometry” calculations at the ground state in the gas phase using the ″Density Functional” algorithm with “B3LYP” functional and 6-31G* polarization basis set. The energies of the frontier molecular orbitals were calculated and reported in electron volts.

### Molecular Docking Study

The molecular docking study starts by minimizing the energy of the ligands (fluorophores) structures in Spartan software^71^ and then importing them into PyRx software as mol. files. The crystal structures of BSA and HPA were downloaded from the Research Collaboratory for Structural Bioinformatics (RCSB) Protein Data Bank website. For bovine serum albumin, the crystal structure with PDB ID 4JK4 was used in the docking study,^78^ and for human parvalbumin, the PDB ID 9BB8 was used.^79^ The protein structures were first imported into Discovery Studio Visualizer^80^ to remove one of the identical chains in each protein, and then the structure was saved as a PDB file, which was prepared in PyRx software^77^ using the “Make macromolecule” command. The docking was done using AutoDock integrated in PyRx, where the prepared protein was chosen as the “protein” using the ″Make macromolecule” command, and the fluorophore structures were chosen as the ligand using the ″Convert to Autodock Ligand (pdbqt)” command. Since the exact binding site was not known in each of the proteins, blind docking was used, in which the whole protein chain was chosen as the grid box for the docking. The docking grid box for the BSA has the following dimensions in Angstroms: “X: 91.3471, Y: 61.5461, Z: 73.2728”, and that for HPA has the following dimensions in Angstroms: “X: 30.7483, Y: 34.3797, Z: 32.4298”. The algorithm provided nine different poses (conformations) for each docking run, which were saved as PDB files, and their energies were reported as binding affinities in kilocalories per mole.

The resulting docking poses were saved as PDB files and then imported into Discovery Studio Visualizer to show the 2D interaction diagrams. The protein structure was marked as the receptor, and the fluorophore pose was marked as the ligand, and then the ″Show 2D diagram” command was chosen to show the 2D interaction diagrams. The PDB files of the different fluorophore poses were also imported into ChimeraX software to show the 3D interaction diagrams.^81,82^ Two 3D diagrams were snapped in the software: one using the “Cartoon” visualization of the protein and another using the “Surface” visualization of the protein. Distances of the hydrogen bond interactions were measured by using the “Distance” annotation command in the software.

### Fluorescence Enhancement with Protein Binding Studies

Stock solutions of the proteins, substrates, or ions were prepared in 100% deionized water. Table 9 shows the sources of the ions used.

**Table 9:** Sources of the Ions

Source	Ion	Source	Ion
Aluminum nitrate	Nitrate (NO_3_^–^)	Sodium bisulfite	bisulfite (HSO_3_^–^)
Sodium nitrite	Nitrite (NO_2_^–^)	Sodium thiosulfate-5-hydrate	thiosulfate (S_2_O_3_^–2^)
Sodium sulfite	Sulfite (SO_3_^–2^)	Sodium hypochlorite	Hypochlorite (OCl^–^)

Solutions of the peptidomimetic dyes **TEA1–6** were prepared in 1× PBS buffer at a concentration of 10 μM, and their fluorescence was measured before the addition of the protein or substrate solutions. Increasing amounts of protein or substrate solutions were added gradually to the peptidomimetic dye solutions, and the fluorescence was recorded after each addition. The fluorescence intensity was recorded on a Shimadzu RF-5301PC spectrofluorometer.

### Viscosity Sensing Studies

Six different deionized water/glycerol solutions were prepared with ratios ranging from 0% glycerol to 100% glycerol. 10 μM solutions of **TEA1** and 3 μM solutions of each of the other dyes (**TEA2–6**) were used for both absorbance and fluorescence viscosity-sensing studies.

## Supplementary Material





























## Notes

^1^HNMR, ^13^CNMR, and HRMS spectra of the synthesized dyes, their photophysical properties, docking studies screenshots, and fluorescence enhancement with protein binding studies (PDF)
Videos showing the docking studies’ expected binding mode of the highest ranked poses of each dye inside the binding pockets of each of BSA and HPA; Binding mode of TEA1 inside the binding pocket of HPA as predicted by the docking study (MP4)
Binding mode of TEA2 inside the binding pocket of HPA as predicted by the docking study (MP4)
Binding mode of TEA3 inside the binding pocket of HPA as predicted by the docking study (MP4)
Binding mode of TEA4 inside the binding pocket of HPA as predicted by the docking study (MP4)
Binding mode of TEA5 inside the binding pocket of HPA as predicted by the docking study (MP4)
Binding mode of TEA6 inside the binding pocket of HPA as predicted by the docking study (MP4)
Binding mode of TEA1 inside the binding pocket of BSA as predicted by the docking study (MP4)
Binding mode of TEA2 inside the binding pocket of BSA as predicted by the docking study (MP4)
Binding mode of TEA3 inside the binding pocket of BSA as predicted by the docking study (MP4)
Binding mode of TEA4 inside the binding pocket of BSA as predicted by the docking study (MP4)
Binding mode of TEA5 inside the binding pocket of BSA as predicted by the docking study (MP4)
Binding mode of TEA6 inside the binding pocket of BSA as predicted by the docking study (MP4)
Molecular formula string (CSV)

## Abbreviations

BSA: bovine serum albumin
DFT: density-functional theory
DMSO: dimethyl sulfoxide
DCM: dichloromethane
eq: equivalent
eV: electron Volt
FDA: Food and Drug Administration
HEPES: 4-(2-Hydroxyethyl)-1-piperazineethanesulfonic acid
HAS: human serum albumin
HBTU: hexafluorophosphate benzotriazole tetramethyl uranium
HCl: hydrochloric acid
HPA: human parvalbumin
ICG: indocyanine green
kcal/mol: kilocalories per mole
LOD: limit of detection
LOQ: limit of quantitation
MeOH: methanol
mW: milliwatt
NIR: near-infrared
nm: nanometer
PBS: phosphate-buffered saline
POCl_3_: phosphorus oxychloride
THF: tetrahydrofuran
TPSA: total polar surface area
UV: ultraviolet
