# Supplementary material for: Peptidomimetic Modified Heptamethine Cyanine Dyes for Enhanced Bioimaging Targetability: A Molecular Interaction Study on Bovine Serum Albumin and Human Parvalbumin
Source: J Med Chem. 2026 Mar 20;69(7):7995–8019. doi: 10.1021/acs.jmedchem.5c03388 (PMC13071880; doi:10.1021/acs.jmedchem.5c03388)
Supplement: Supplementary file 1 [file jm5c03388_si_001.pdf]

Supporting Information

Peptidomimetic modified heptamethine cyanine dyes for enhanced bioimaging targetability: A molecular interaction study on bovine serum albumin and human parvalbumin

Tarek E. Ahmed[1], Nasibeh A. Khereshki[1], Maged Henary[1,2*]

[1]Department of Chemistry, Georgia State University, Atlanta, Georgia, 30303

[2]Center For Diagnostics and Therapeutics, Georgia State University, Atlanta, Georgia, 30303

*Address correspondence to
Dr. Maged Henary
Professor and Associate Chair of Chemistry
Department of Chemistry
Georgia State University
Atlanta, Georgia 30303
USA
mhenary1@gsu.edu
Phone: 404-413-5566

Table of Contents

1. ^{1}HNMR, ^{13}CNMR, and HRMS Spectra 3

2. Absorbance spectra, Calibration curves and fluorescence spectra of the synthesized fluorophores 28

3. Hydrophobicity Studies 34

4. DFT Studies for calculation of HOMO and LUMO orbitals 35

5. Overlapped normalized absorbance and fluorescence spectra for determining the wavelength of the 0-0 transition 36

6. Docking Studies 37

6.1. Docking figures of the synthesized dyes with bovine serum albumin (BSA) 37

6.2. Docking figures of the synthesized dyes with human parvalbumin (HPA) 45

7. Fluorescence enhancement protein binding studies 53

7.1. Kinetic Analysis 53

7.2. Kinetic analysis at different pH values 62

7.3. Calculation of the binding constant of the dyes with BSA and HPA using the Benesi-Hildebrand plot 64

7.4. Fluorescence intensity vs concentration of BSA or HPA for the calculation of limit of detection (LOD) and limit of quantitation (LOQ) 66

7.5. Selectivity studies 68

7.6. Viscosity sensing studies 70

1. ^{1}HNMR, ^{13}CNMR, and HRMS Spectra

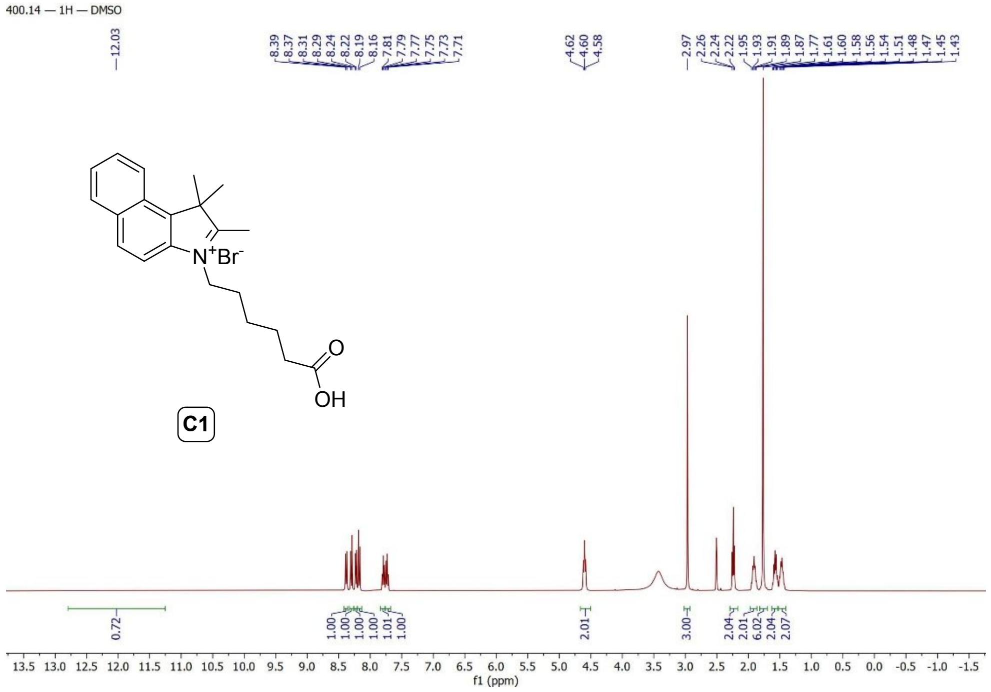

Figure S1. ^{1}H NMR spectrum of **C1** in DMSO-d_6 (400 MHz)

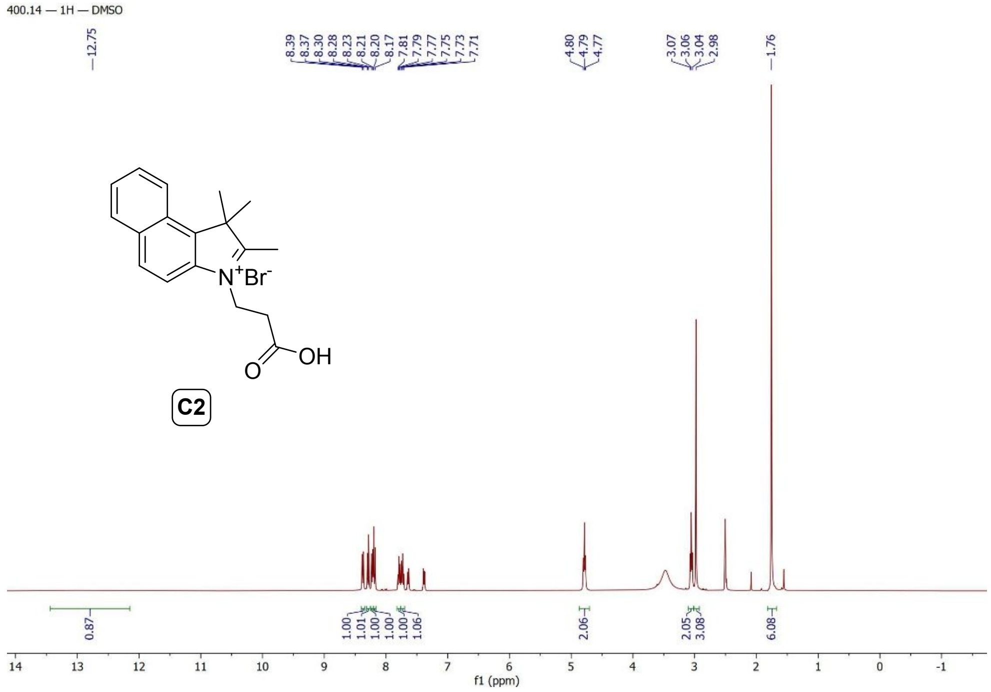

Figure S2. ^{1}H NMR spectrum of **C2** in DMSO-d_6 (400 MHz)

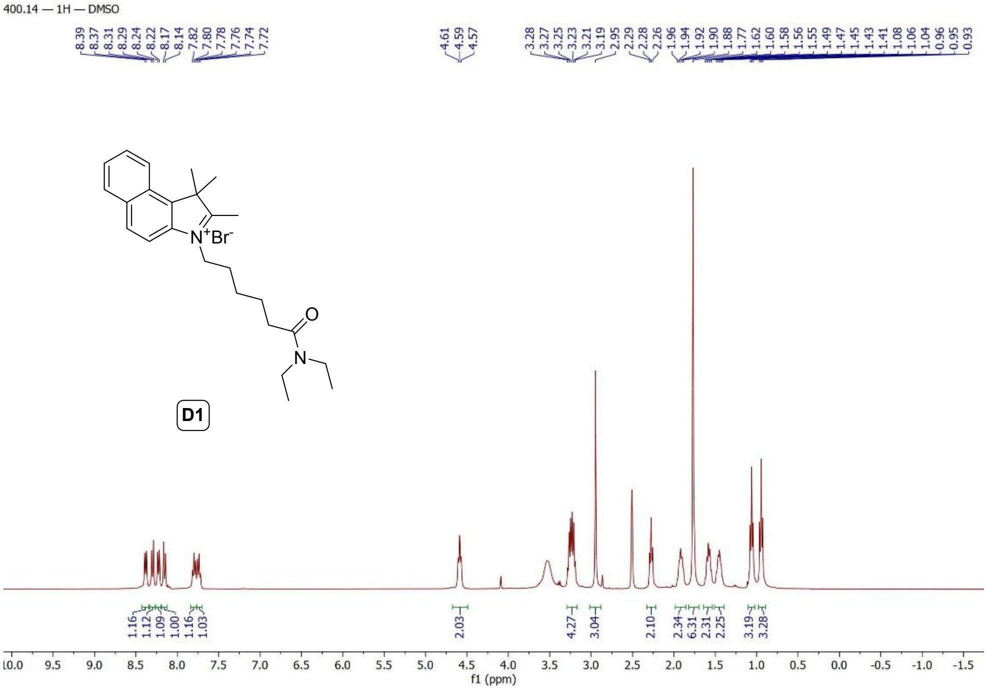

Figure S3. ^{1}H NMR spectrum of **D1** in DMSO-d_6 (400 MHz)

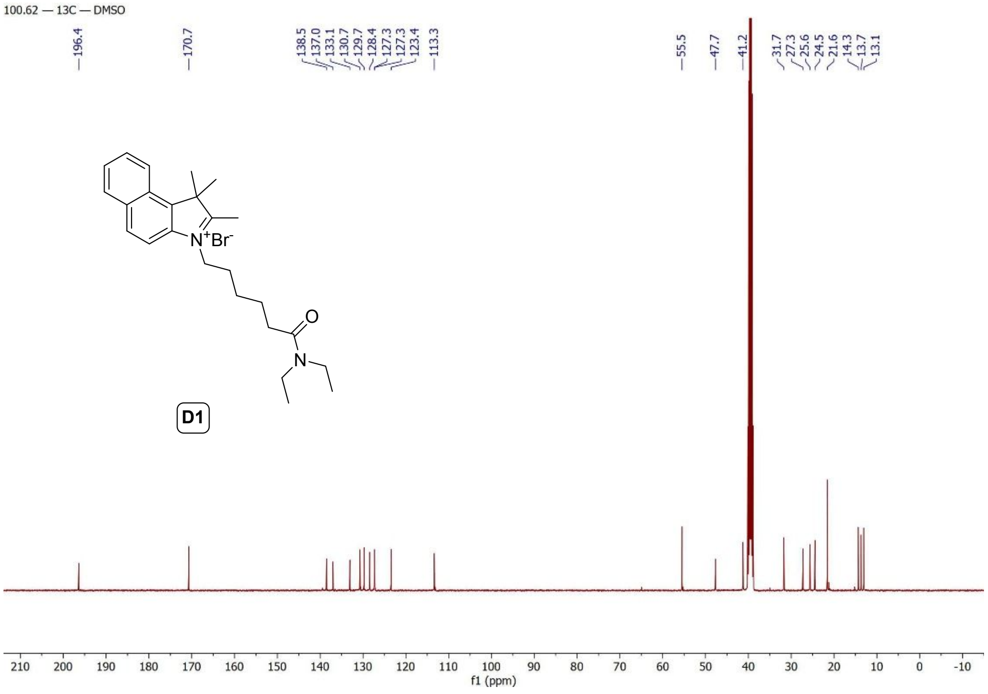

Figure S4. ^{13}C NMR spectrum of **D1** in DMSO-d_6 (101 MHz)

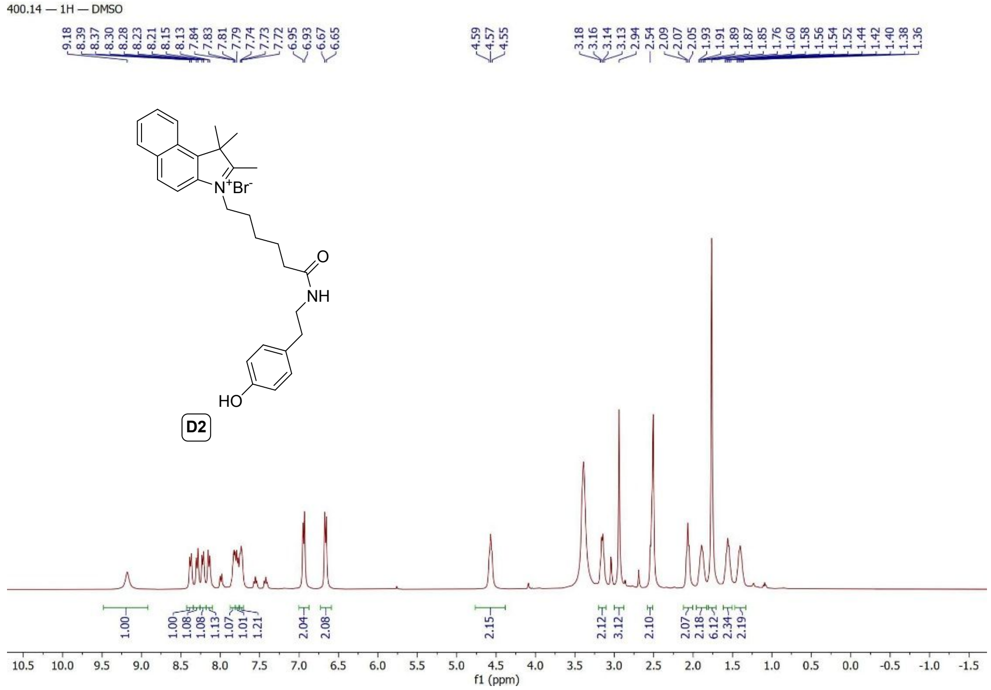

Figure S5. ^{1}H NMR spectrum of **D2** in DMSO-d_6 (400 MHz)

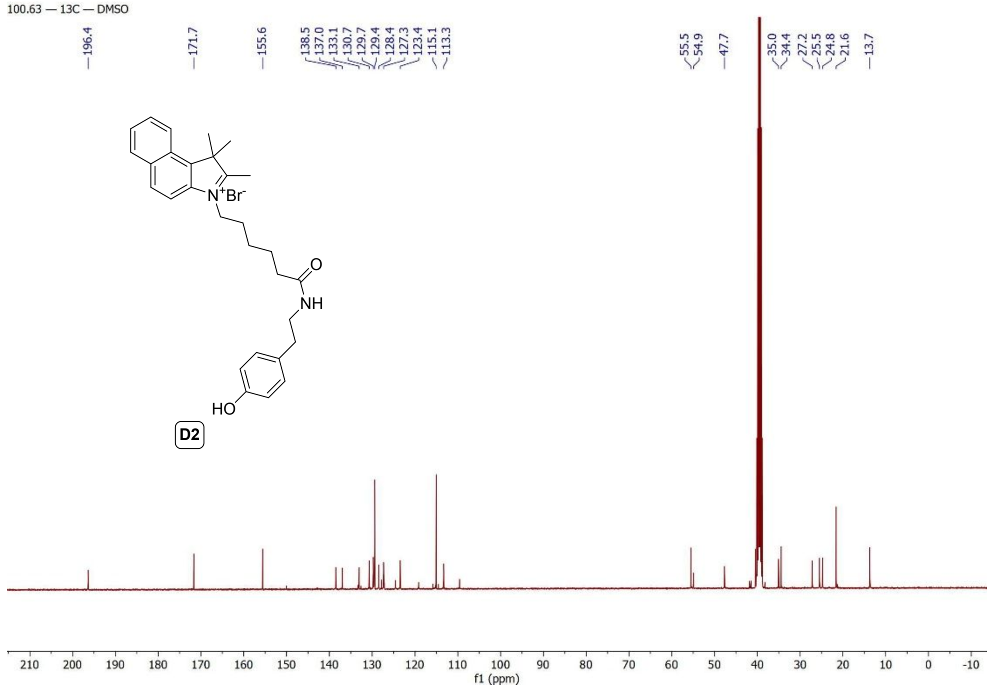

Figure S6. ^{13}C NMR spectrum of **D2** in DMSO-d_6 (101 MHz)

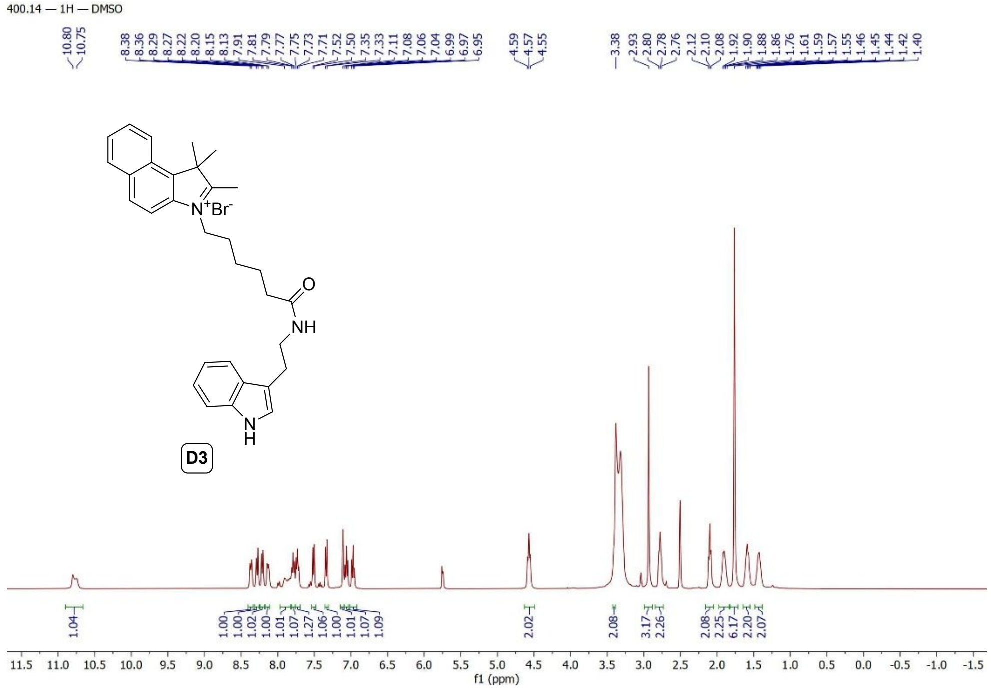

Figure S7. ^{1}H NMR spectrum of **D3** in DMSO-d_6 (400 MHz)

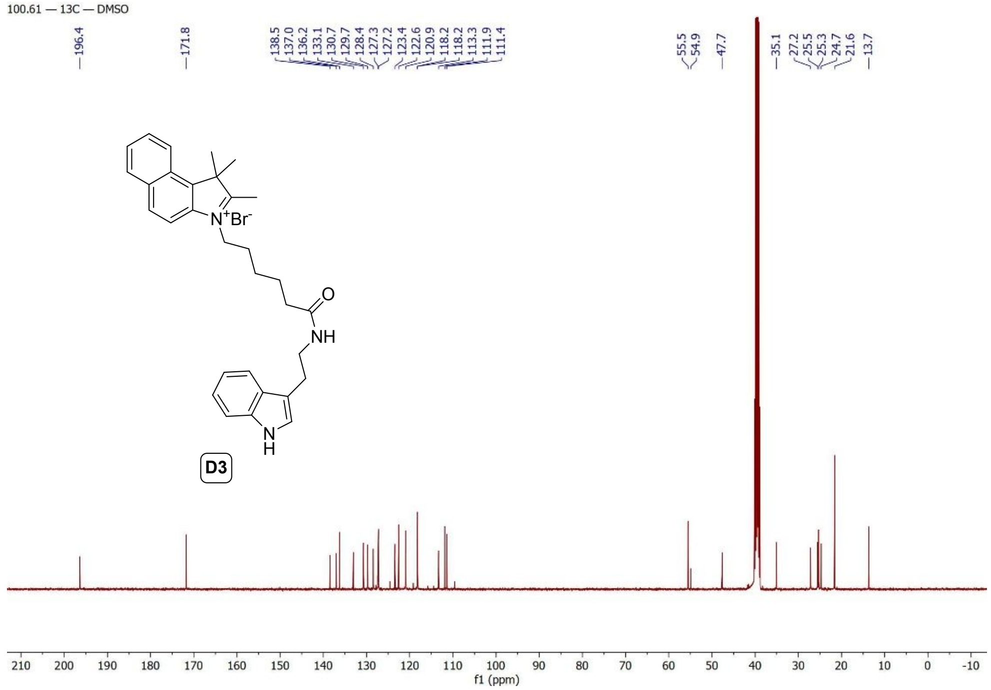

Figure S8. ^{13}C NMR spectrum of **D3** in DMSO-d_6 (101 MHz)

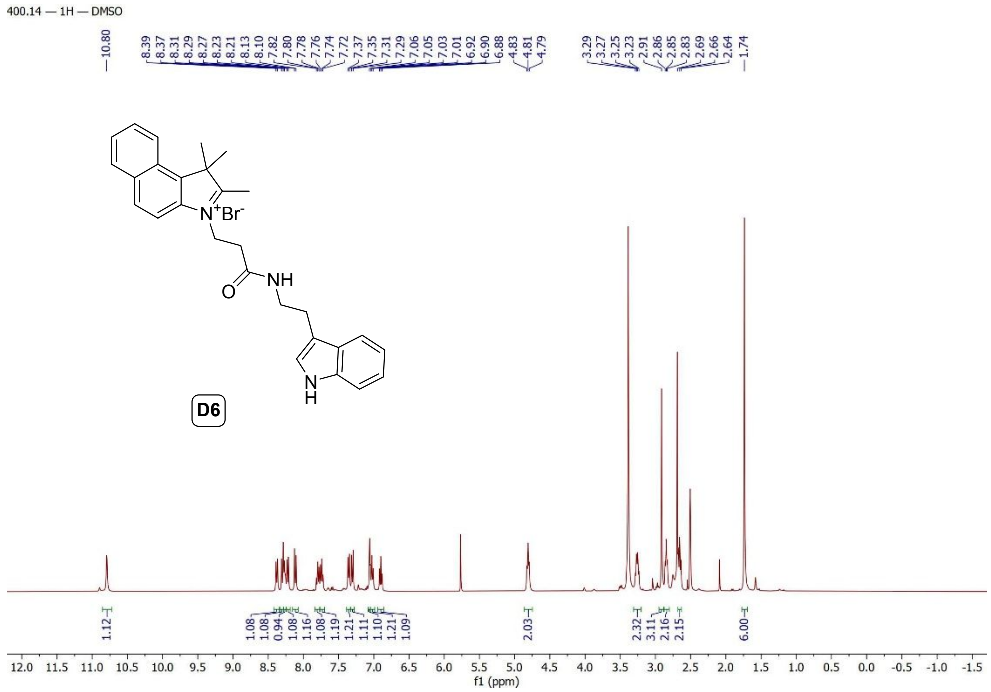

Figure S9. ^{1}H NMR spectrum of **D6** in DMSO-d_6 (400 MHz)

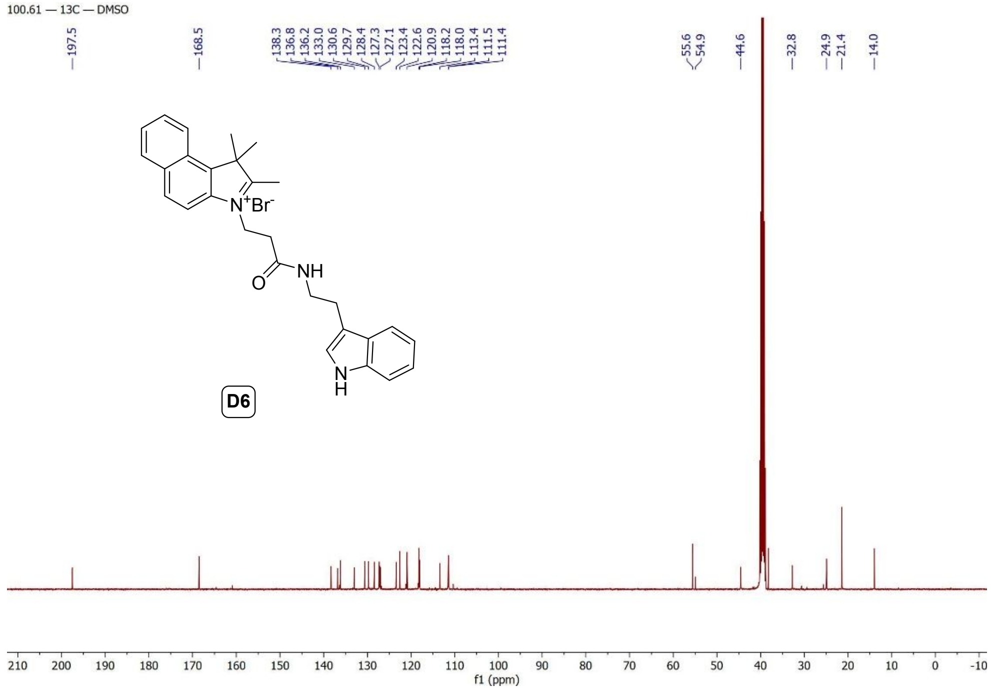

Figure S10. ^{13}C NMR spectrum of **D6** in DMSO-d_6 (101 MHz)

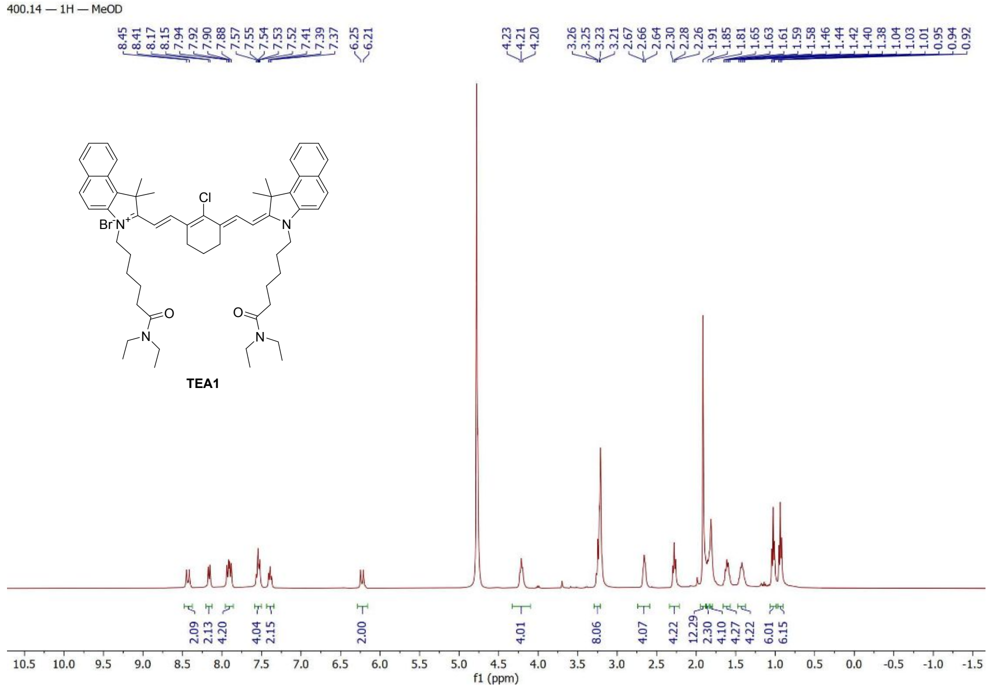

Figure S11. ^{1}HNMR spectrum of **TEA1** in MeOD (400 MHz)

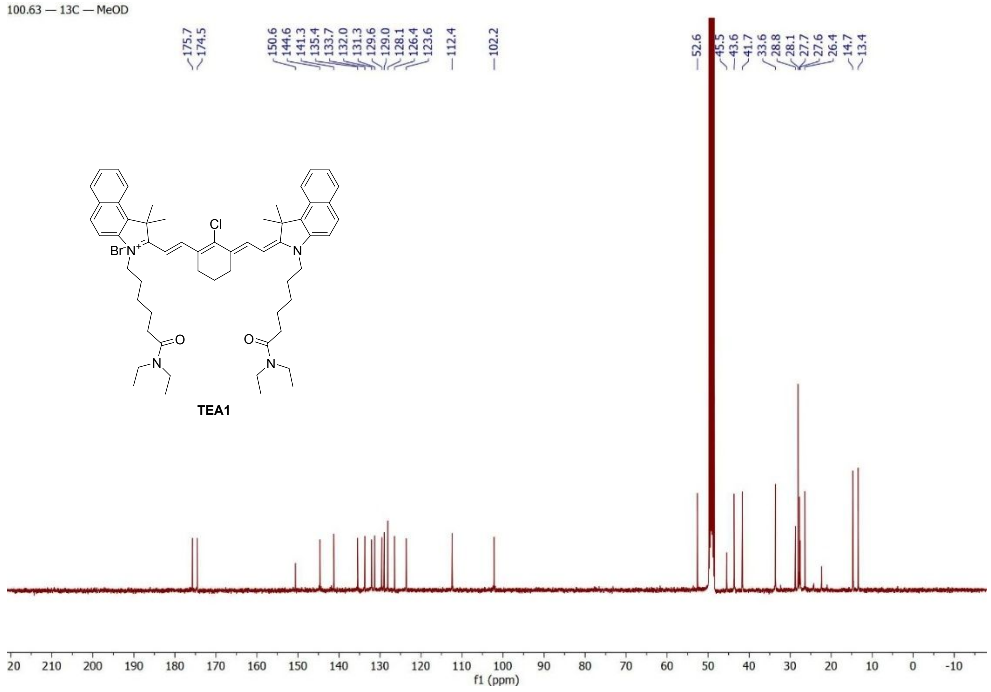

Figure S12. ^{13}CNMR spectrum of **TEA1** in MeOD (101 MHz)

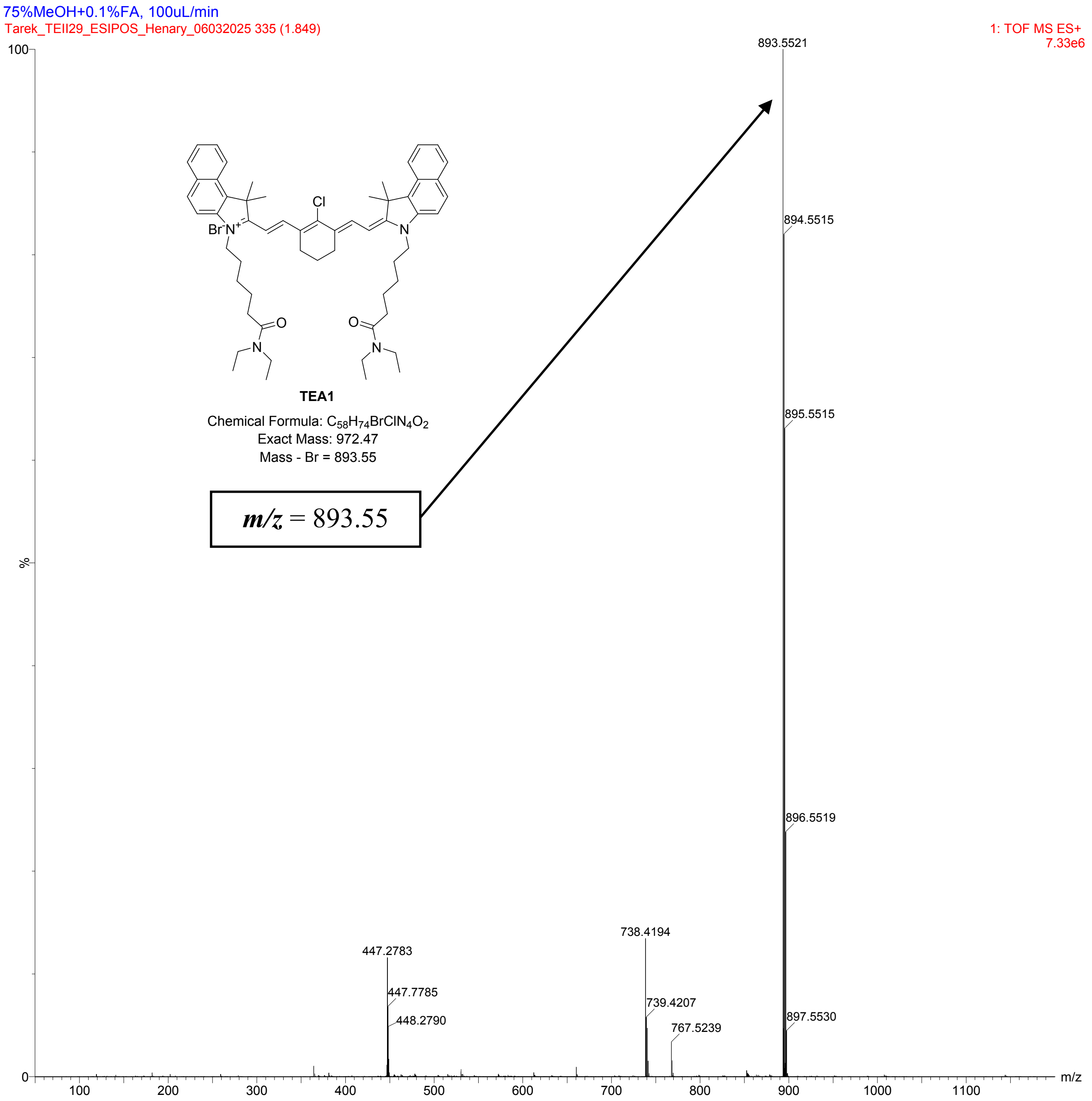

Figure S13a. HRMS spectrum of **TEA1**

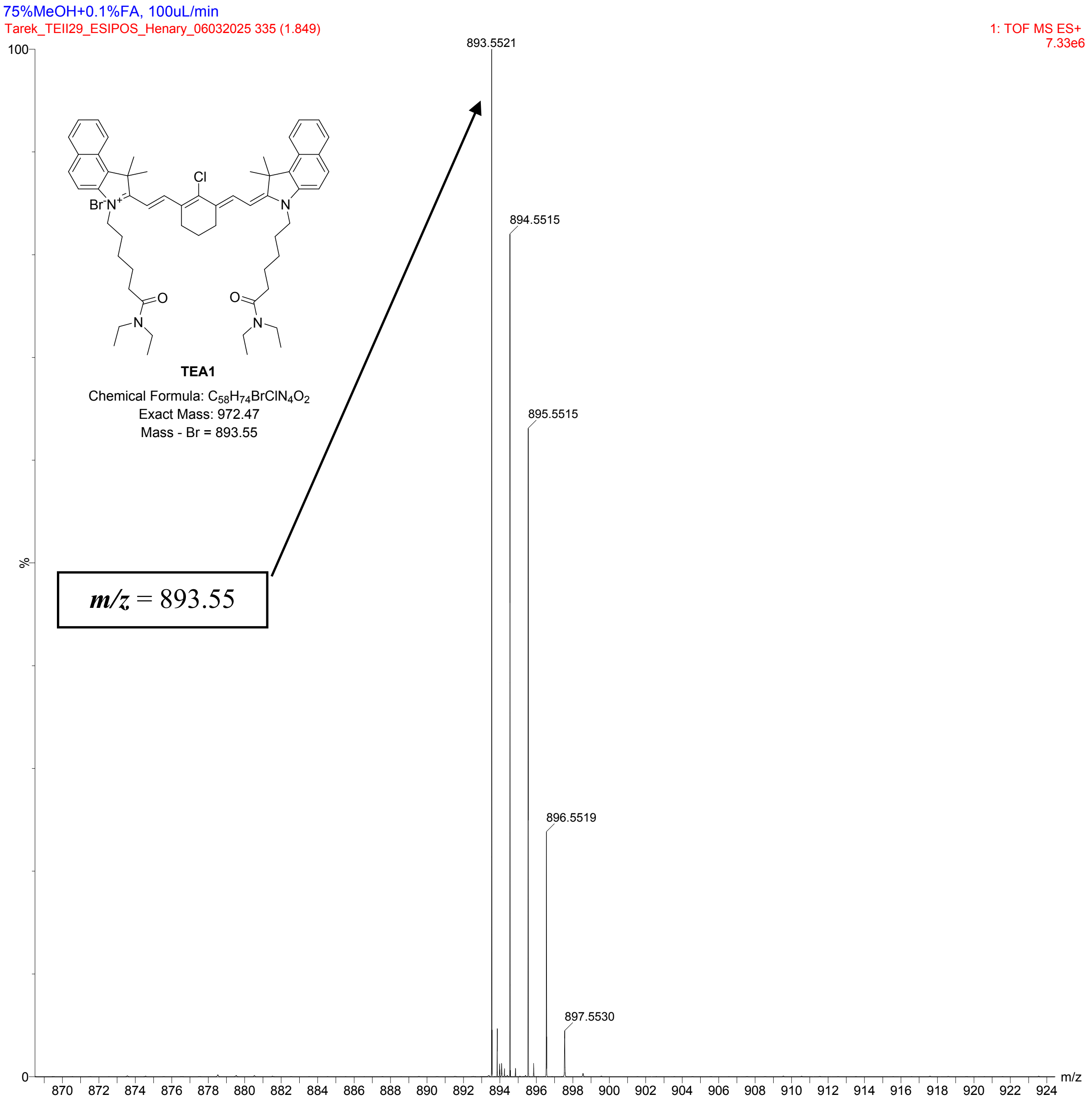

Figure S13b. HRMS spectrum of **TEA1**

Elemental Composition Report

Single Mass Analysis

Tolerance = 10.0 PPM / DBE: min = -50.0, max = 500.0

Element prediction: Off

Monoisotopic Mass, Odd and Even Electron Ions

4870 formula(e) evaluated with 3 results within limits (all results (up to 1000) for each mass)

Elements Used:

C: 58-58 H: 0-100 N: 0-30 O: 0-50 Cl: 0-3

Minimum:				-50.0		
Maximum:		1000.0		10.0	500.0	
Mass	Calc. Mass	mDa	PPM		DBE	Formula
893.5521	893.5500	2.1	2.4	23.5	C58 H74 N4 O2 Cl	

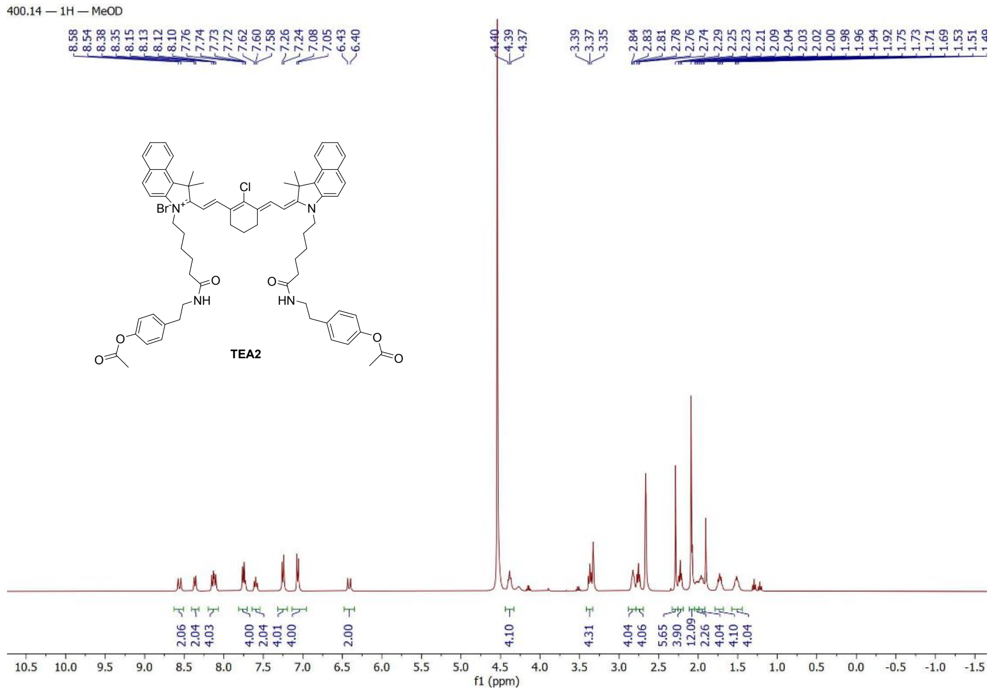

Figure S14. ^{1}HNMR spectrum of **TEA2** in MeOD/DMSO (400 MHz)

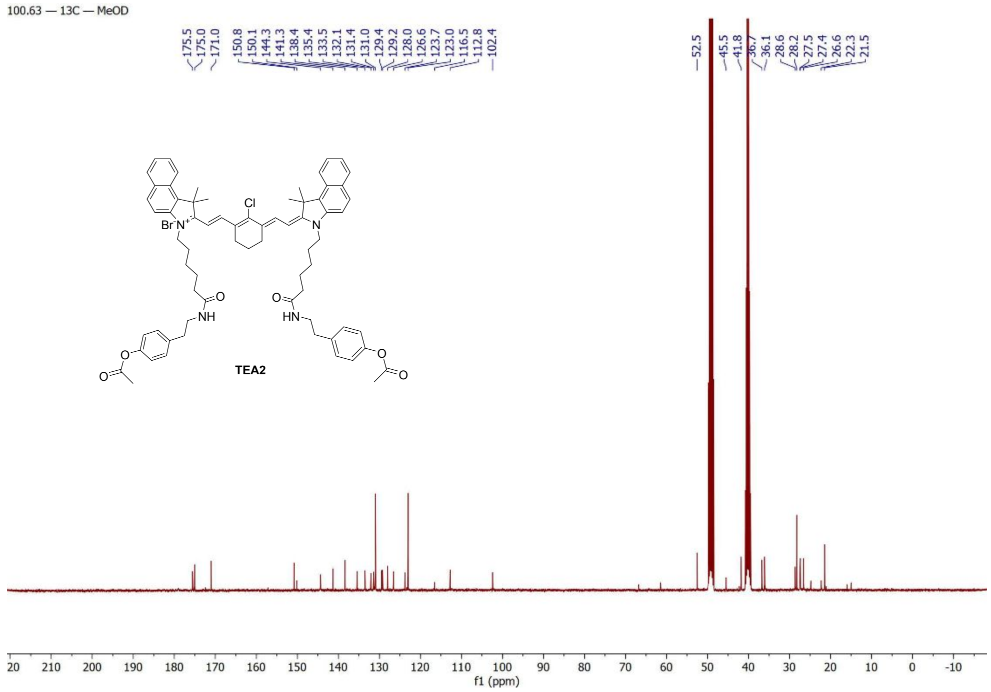

Figure S15. ^{13}CNMR spectrum of **TEA2** in MeOD/DMSO (101 MHz)

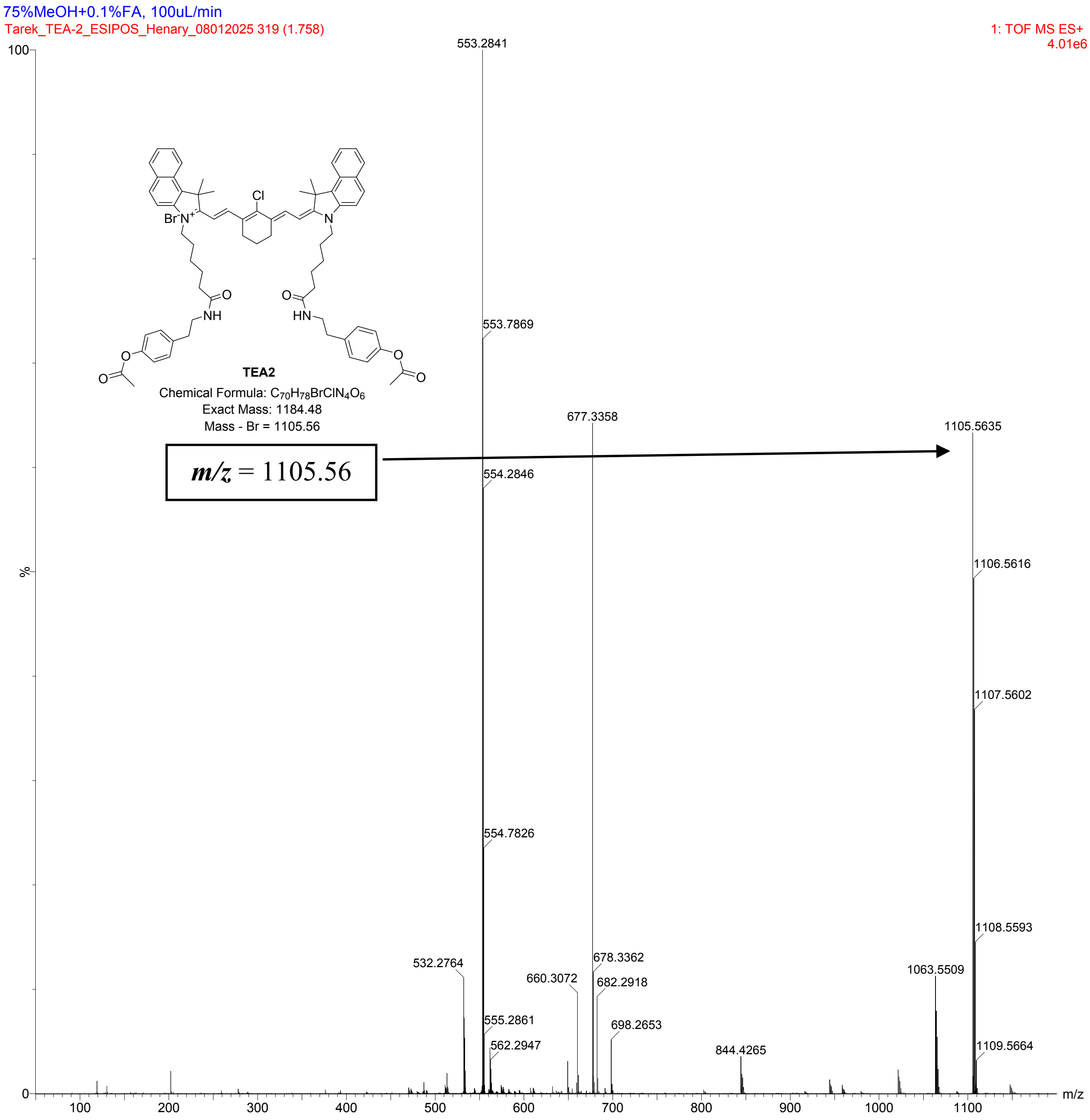

Figure S16. HRMS spectrum of **TEA2**

Elemental Composition Report

Single Mass Analysis

Tolerance = 20.0 PPM / DBE: min = -50.0, max = 500.0

Element prediction: Off

Monoisotopic Mass, Even Electron Ions

8995 formula(e) evaluated with 11 results within limits (all results (up to 1000) for each mass)

Elements Used:

C: 70-70 H: 0-200 N: 0-30 O: 0-50 S: 0-2 Cl: 0-1

Minimum:				-50.0		
Maximum:		1000.0		20.0	500.0	
Mass	Calc. Mass	mDa	PPM		DBE	Formula
1105.5635	1105.5610	2.5	2.3		33.5	C70 H78 N4 O6 Cl

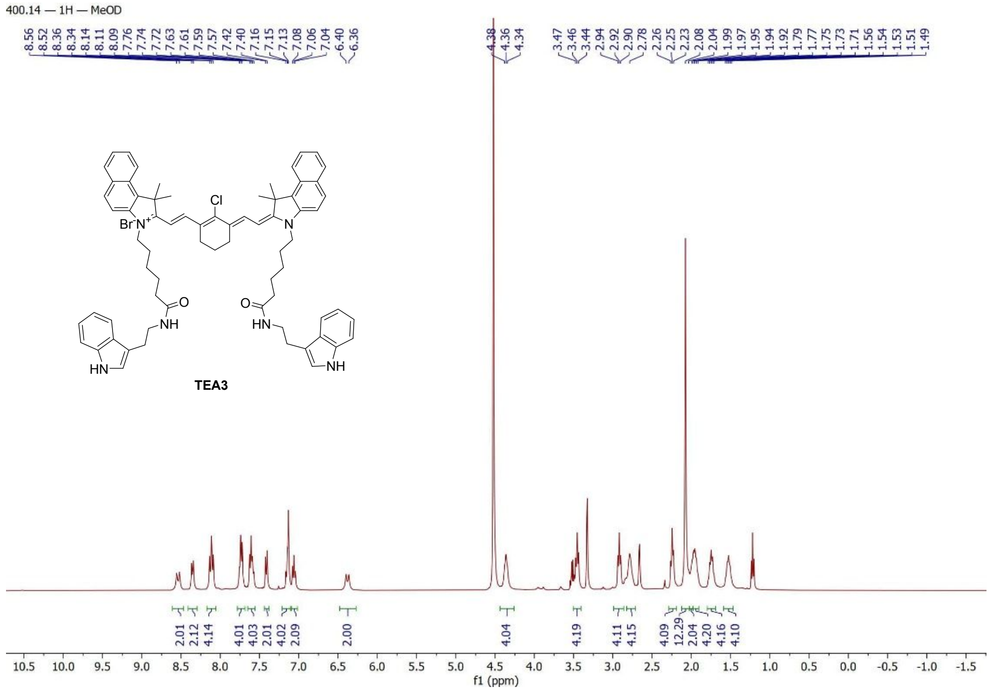

Figure S17. ^{1}HNMR spectrum of **TEA3** in MeOD/DMSO (400 MHz)

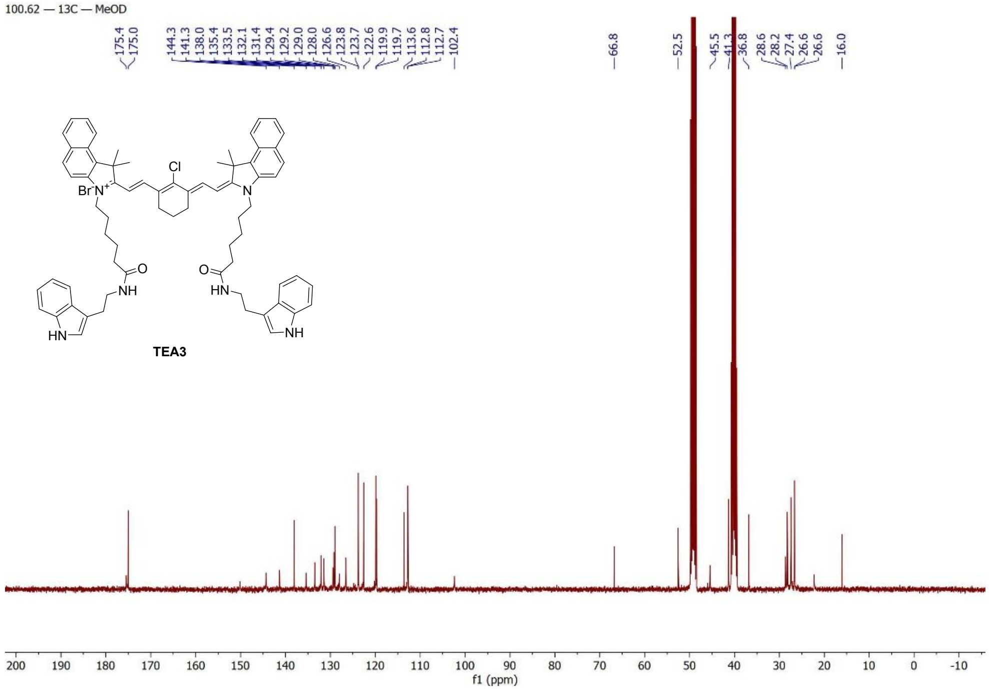

Figure S18. ^{13}CNMR spectrum of **TEA3** in MeOD/DMSO (101 MHz)

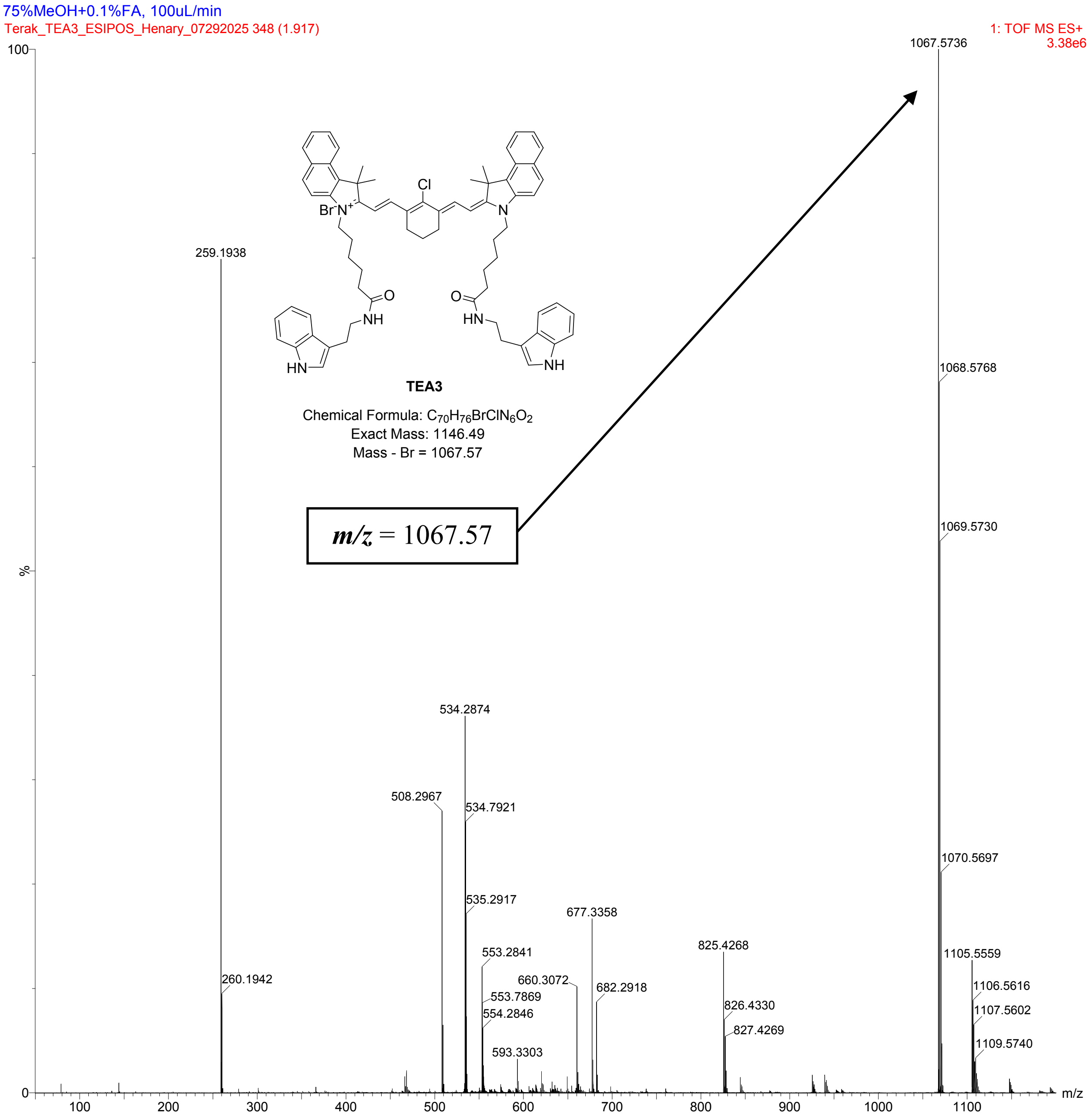

Figure S19a. HRMS spectrum of **TEA3**

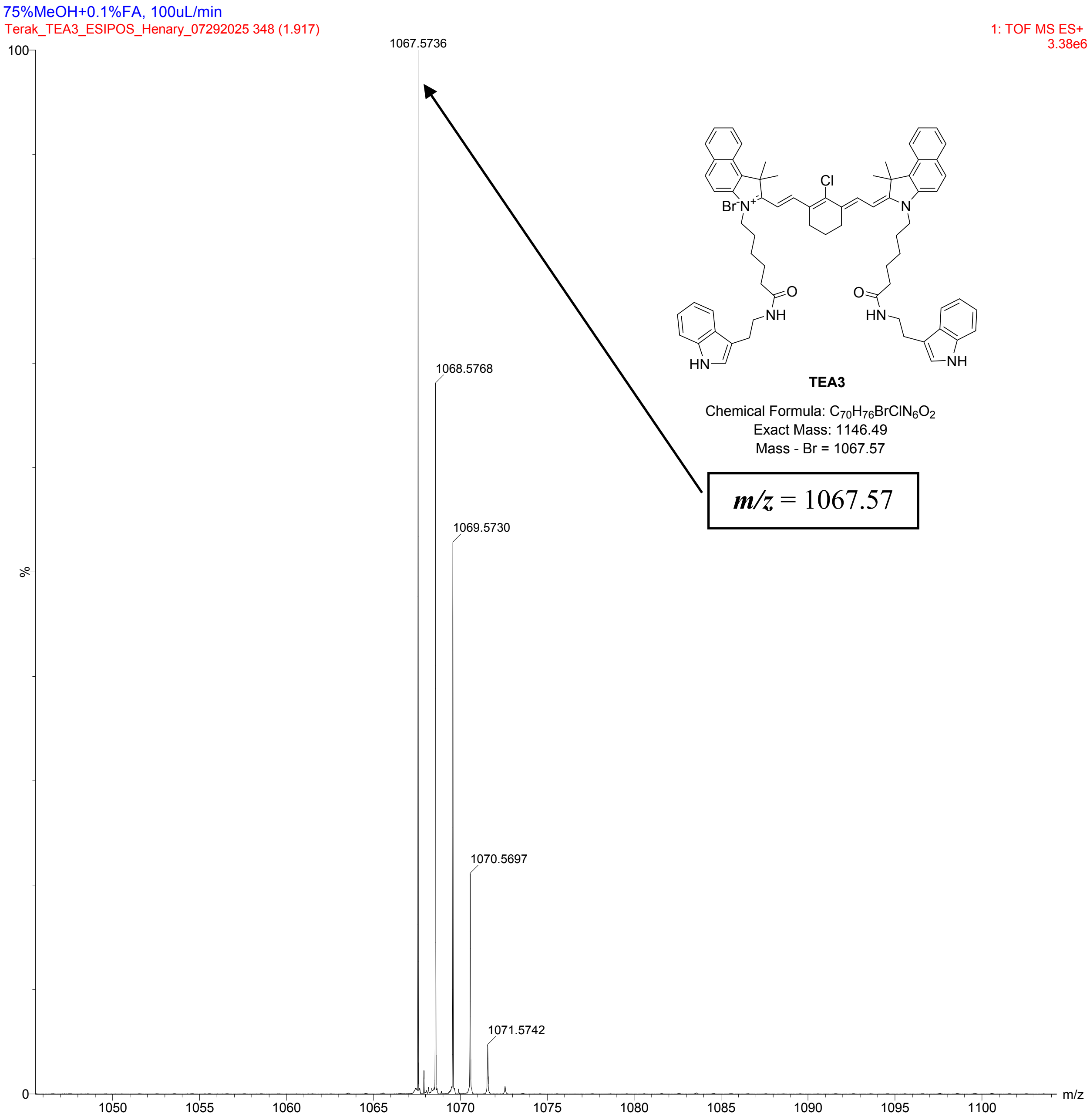

Figure S19b. HRMS spectrum of **TEA3**

Elemental Composition Report

Single Mass Analysis

Tolerance = 20.0 PPM / DBE: min = -50.0, max = 500.0

Element prediction: Off

Monoisotopic Mass, Even Electron Ions

2888 formula(e) evaluated with 3 results within limits (all results (up to 1000) for each mass)

Elements Used:

C: 70-70 H: 0-200 N: 0-30 O: 0-50 Cl: 1-1 Br: 0-1

Minimum:				-50.0		
Maximum:		1000.0		20.0	500.0	
Mass	Calc. Mass	mDa	PPM		DBE	Formula
1067.5736	1067.5718	1.8	1.7	35.5	C70 H76 N6 O2 Cl	

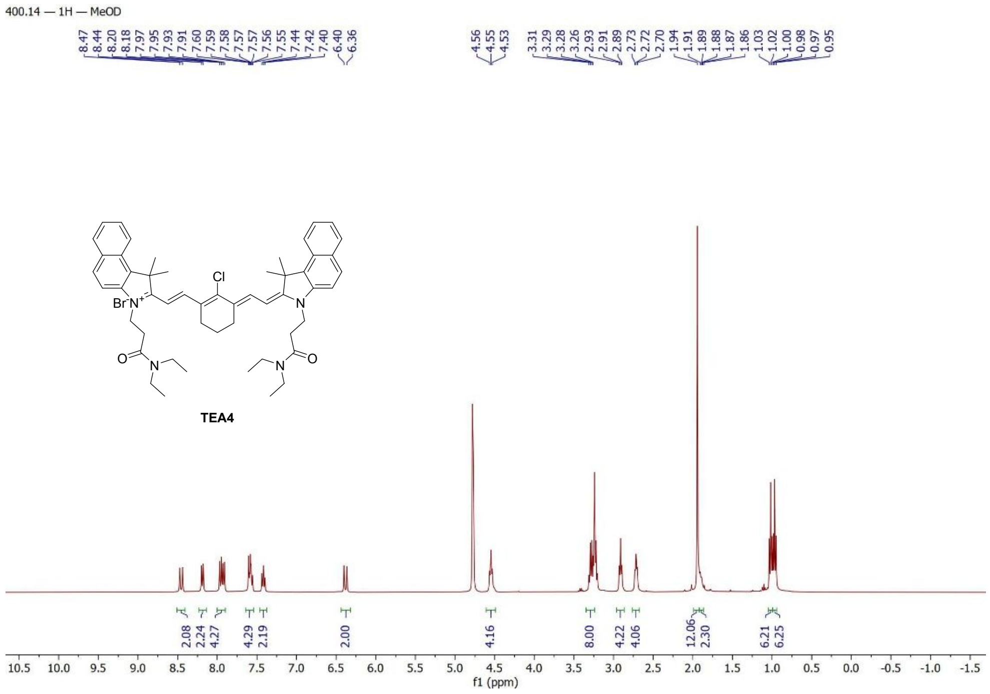

Figure S20. ^{1}HNMR spectrum of **TEA4** in MeOD (400 MHz)

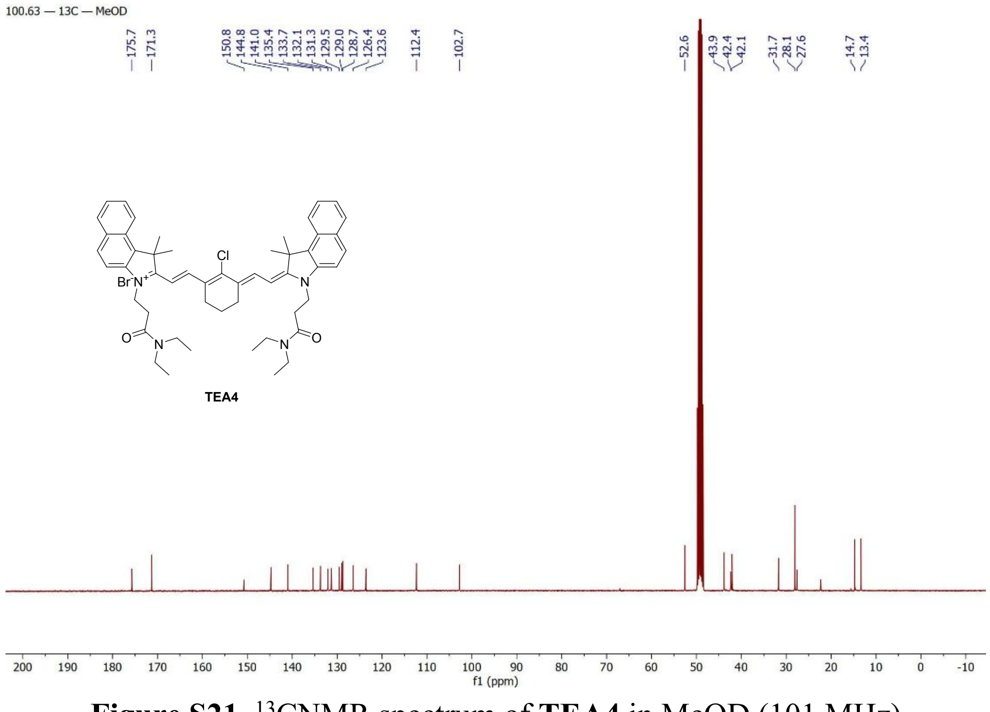

Figure S21. ^{13}CNMR spectrum of **TEA4** in MeOD (101 MHz)

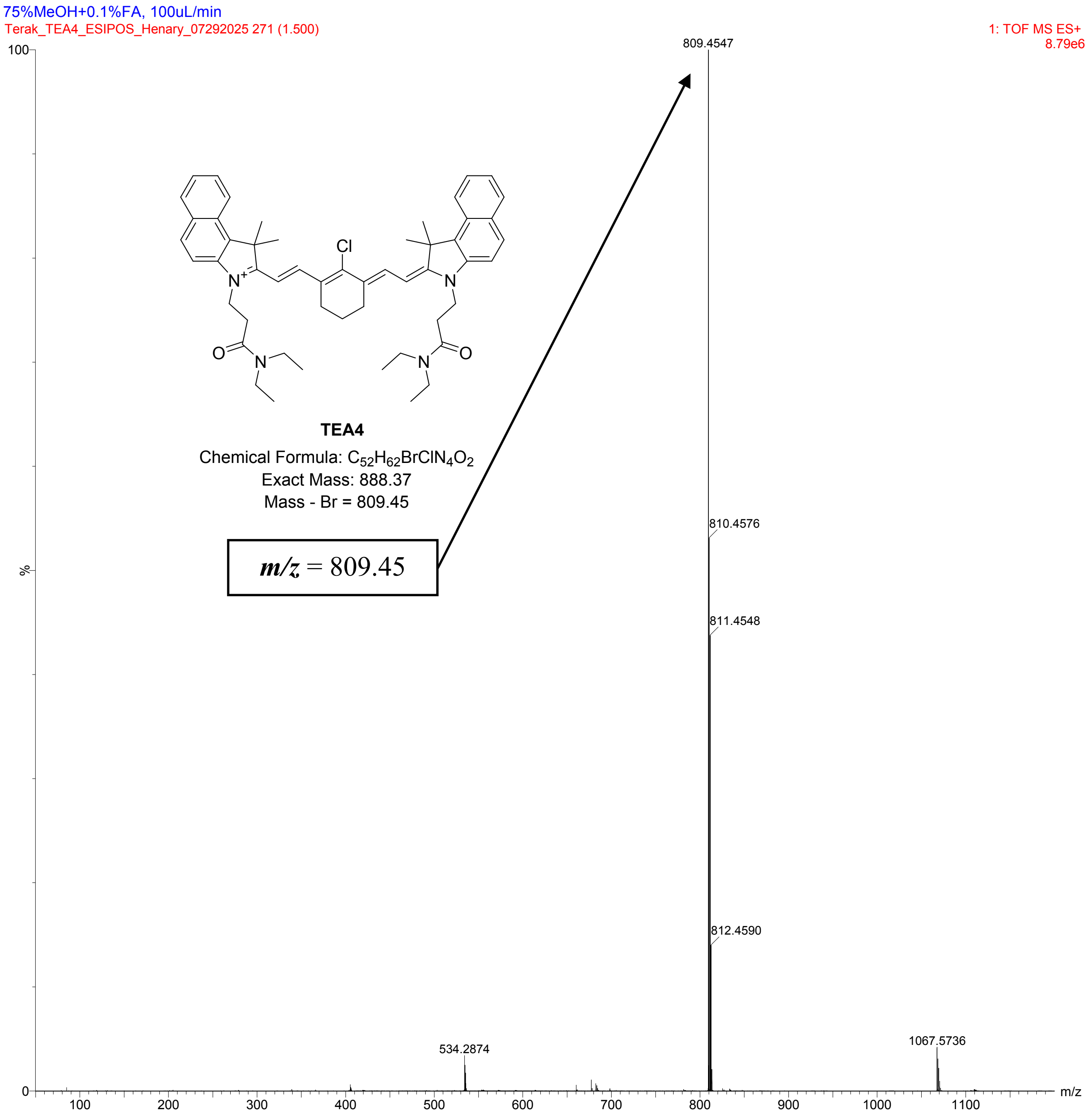

Figure S22. HRMS spectrum of **TEA4**

Elemental Composition Report

Single Mass Analysis

Tolerance = 20.0 PPM / DBE: min = -50.0, max = 500.0

Element prediction: Off

Monoisotopic Mass, Even Electron Ions

5917 formula(e) evaluated with 2 results within limits (all results (up to 1000) for each mass)

Elements Used:

C: 52-52 H: 0-200 N: 0-30 O: 0-50 Na: 0-2 Cl: 1-1 Br: 0-1

Minimum:				-50.0		
Maximum:		1000.0		20.0	500.0	
Mass	Calc. Mass	mDa	PPM		DBE	Formula
809.4547	809.4561	-1.4	-1.7	23.5	C52 H62 N4 O2 Cl	

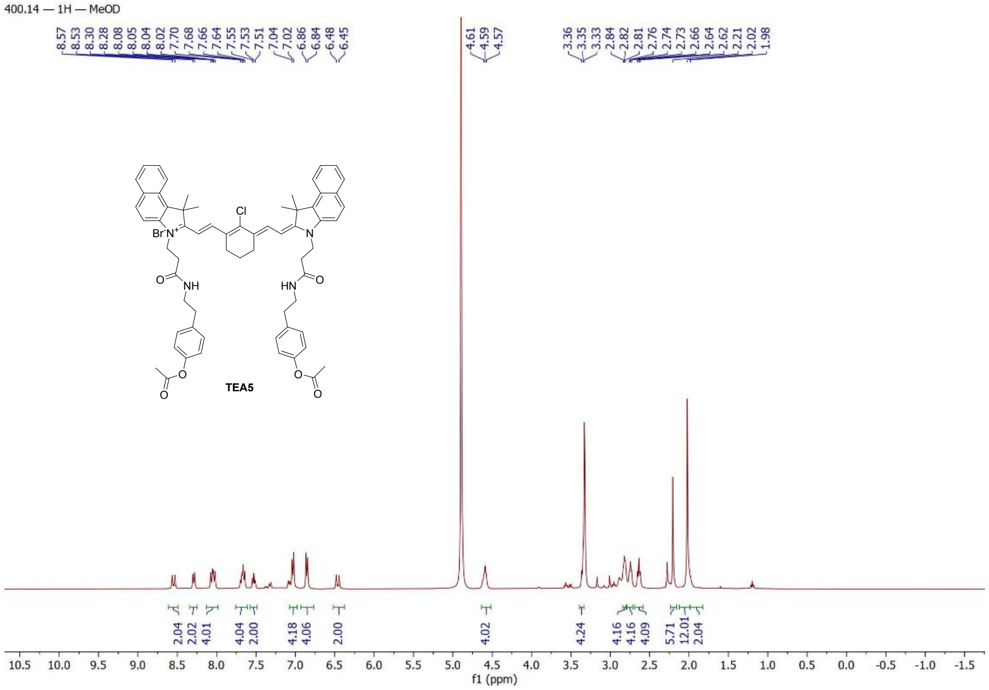

Figure S23. ^{1}HNMR spectrum of **TEA5** in MeOD (400 MHz)

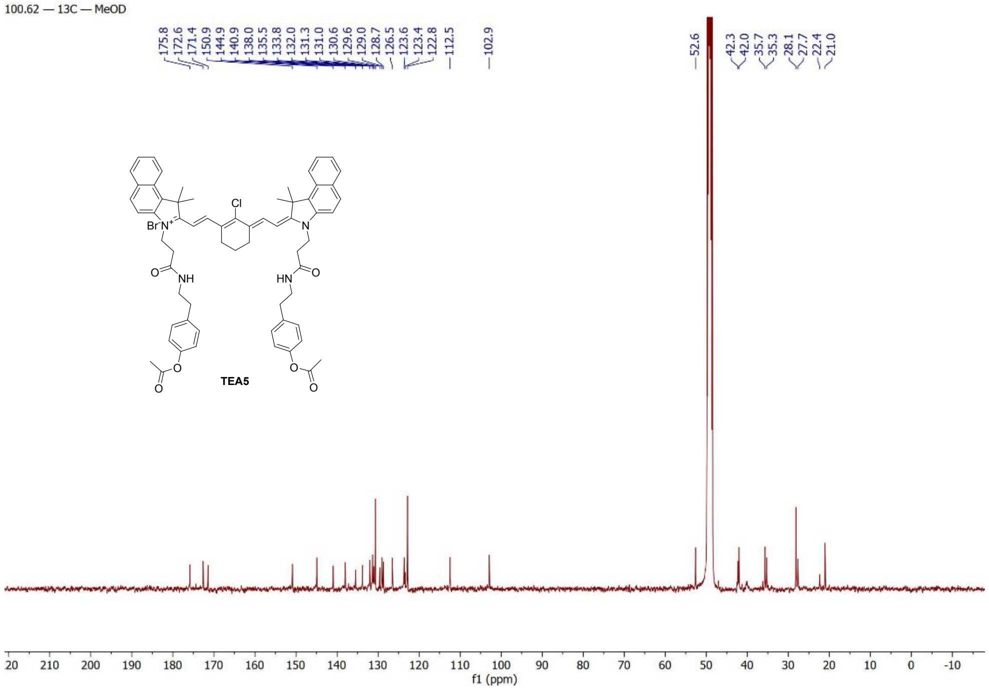

Figure S24. ^{13}CNMR spectrum of **TEA5** in MeOD (101 MHz)

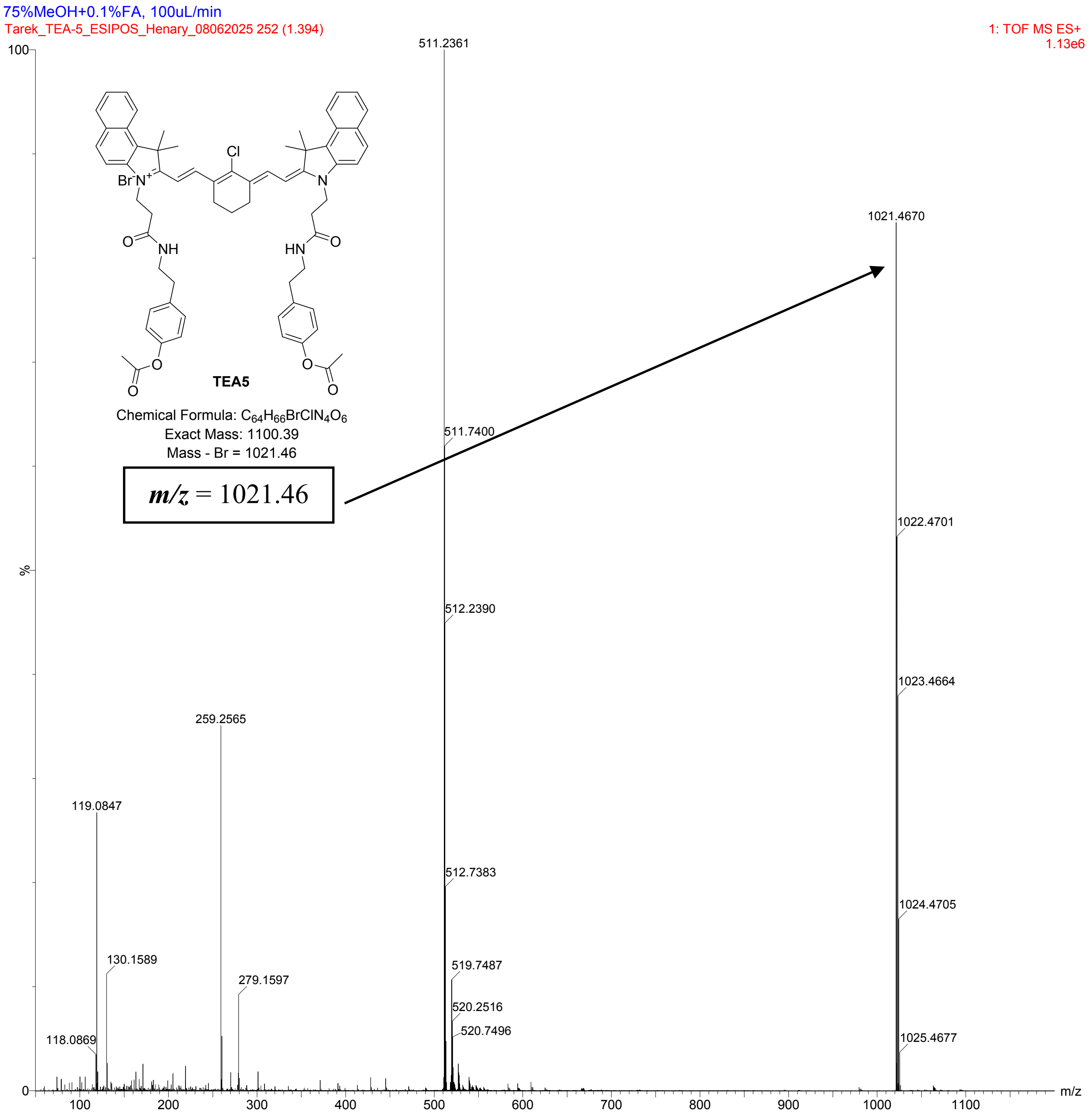

Figure S25. HRMS spectrum of **TEA5**

Elemental Composition Report

Single Mass Analysis

Tolerance = 20.0 PPM / DBE: min = -50.0, max = 500.0

Element prediction: Off

Monoisotopic Mass, Even Electron Ions

8508 formula(e) evaluated with 12 results within limits (all results (up to 1000) for each mass)

Elements Used:

C: 64-64 H: 0-200 N: 0-30 O: 0-50 S: 0-2 Cl: 0-1

Minimum:				-50.0		
Maximum:		1000.0		20.0	500.0	
Mass	Calc. Mass	mDa	PPM		DBE	Formula
1021.4670	1021.4671	-0.1	-0.1	33.5	C64 H66 N4 O6 Cl	

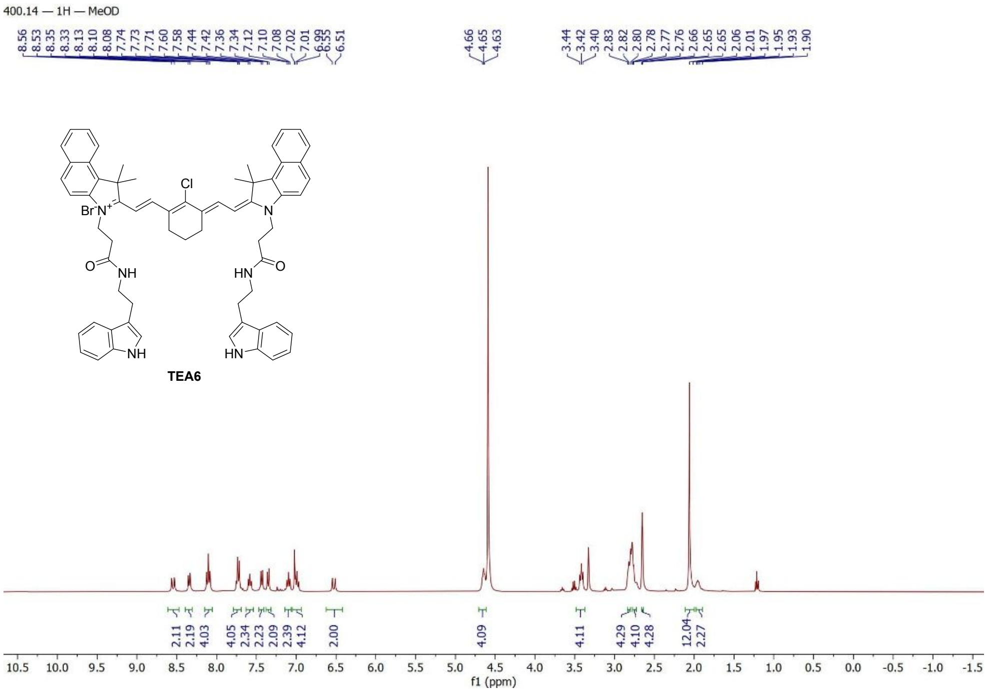

Figure S26. ^{1}HNMR spectrum of **TEA6** in MeOD/DMSO (400 MHz)

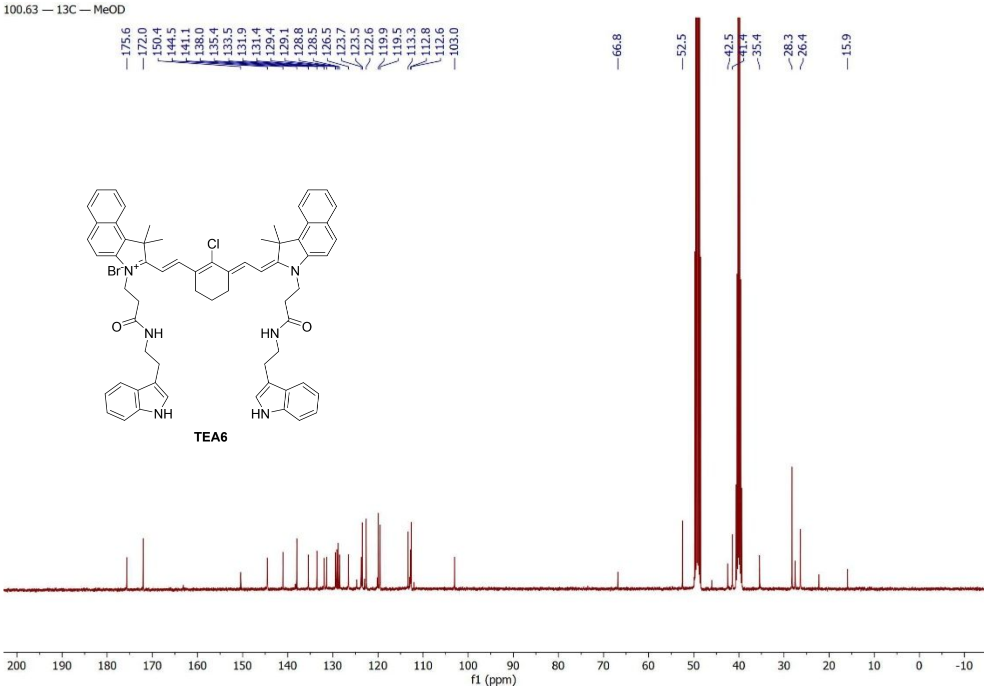

Figure S27. ^{13}CNMR spectrum of **TEA6** in MeOD/DMSO (101 MHz)

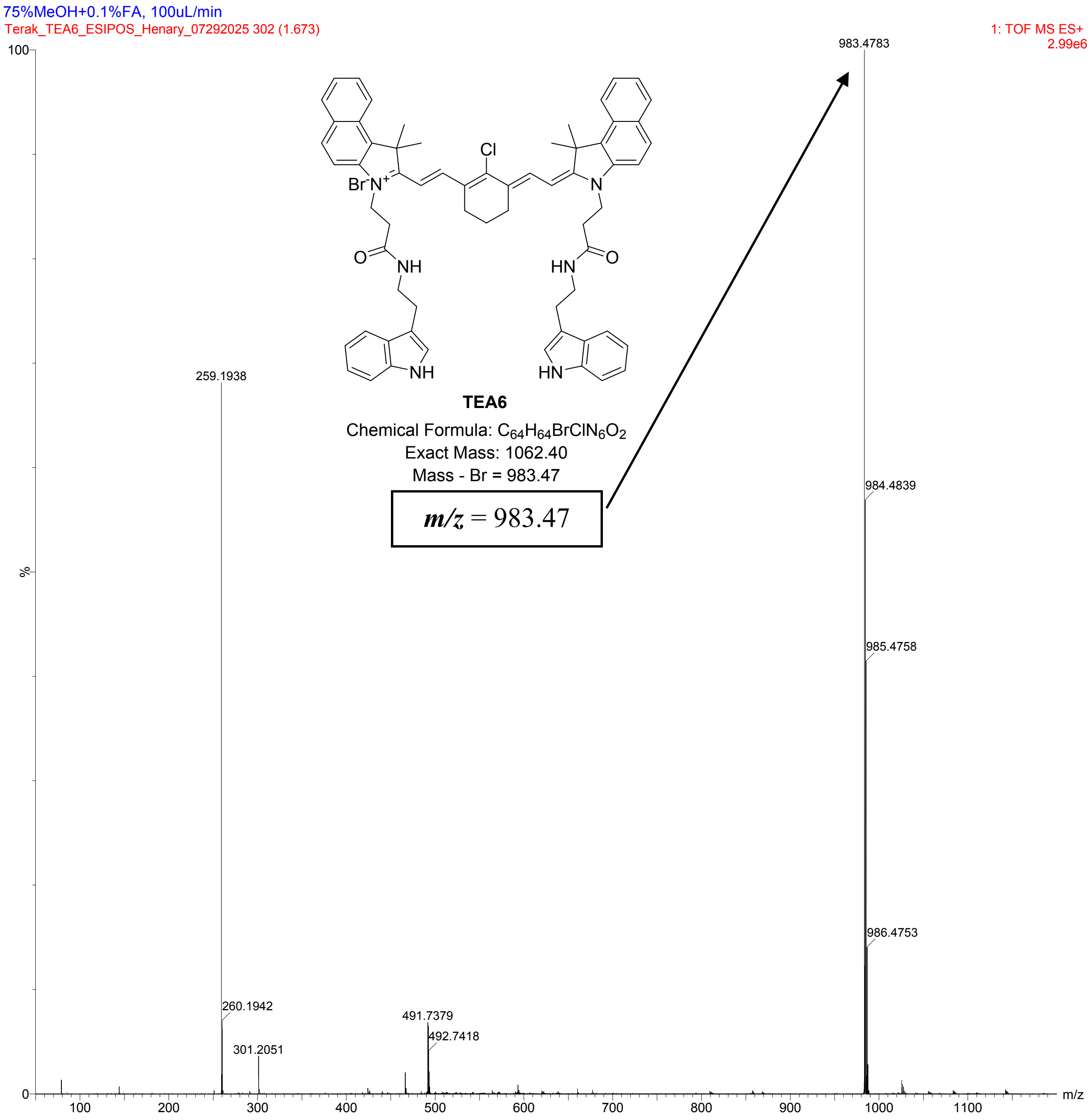

Figure S28. HRMS spectrum of **TEA6**

Elemental Composition Report

Single Mass Analysis

Tolerance = 20.0 PPM / DBE: min = -50.0, max = 500.0

Element prediction: Off

Monoisotopic Mass, Even Electron Ions

2673 formula(e) evaluated with 3 results within limits (all results (up to 1000) for each mass)

Elements Used:

C: 64-64 H: 0-200 N: 0-30 O: 0-50 Cl: 1-1 Br: 0-1

Minimum:				-50.0		
Maximum:		1000.0		20.0	500.0	
Mass	Calc. Mass	mDa	PPM		DBE	Formula
983.4783	983.4779	0.4	0.4	35.5	C64 H64 N6 O2 Cl	

2. Absorbance spectra, Calibration curves and fluorescence spectra of the synthesized fluorophores

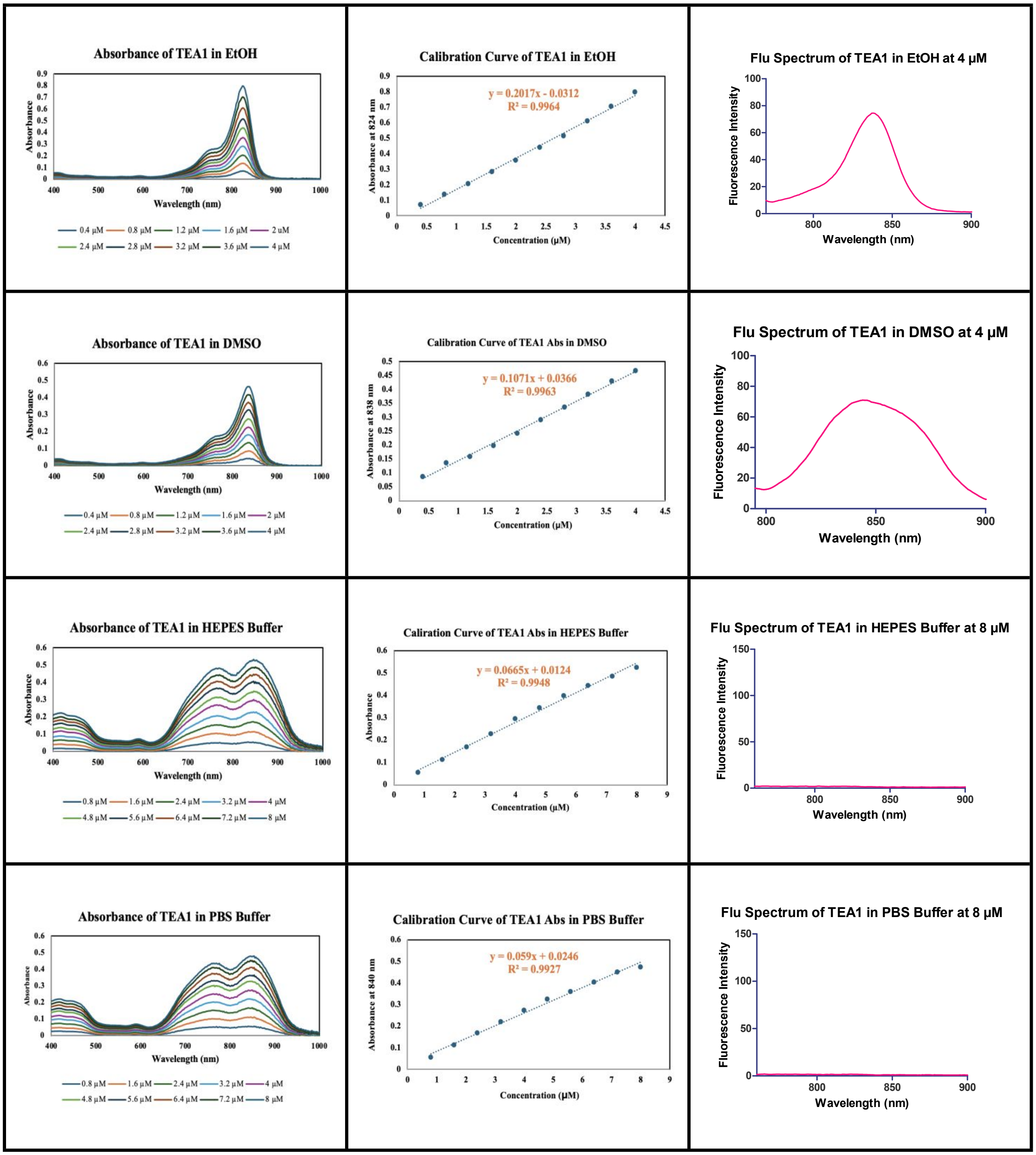

Figure S29. TEA1 Absorbance curves at different concentrations, calibration curves, and fluorescence curves in different solvents

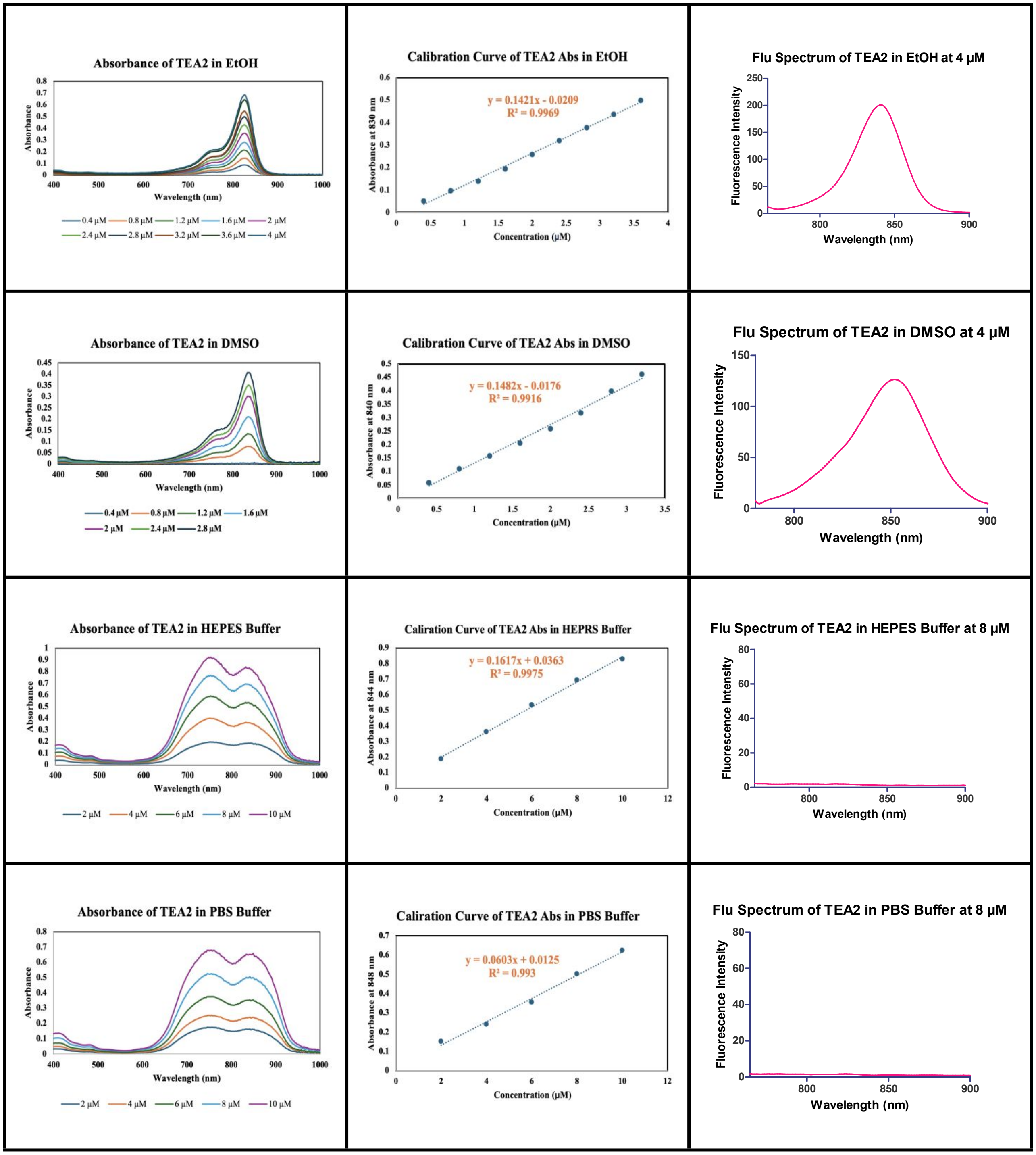

Figure S30. TEA2 Absorbance curves at different concentrations, calibration curves, and fluorescence curves in different solvents

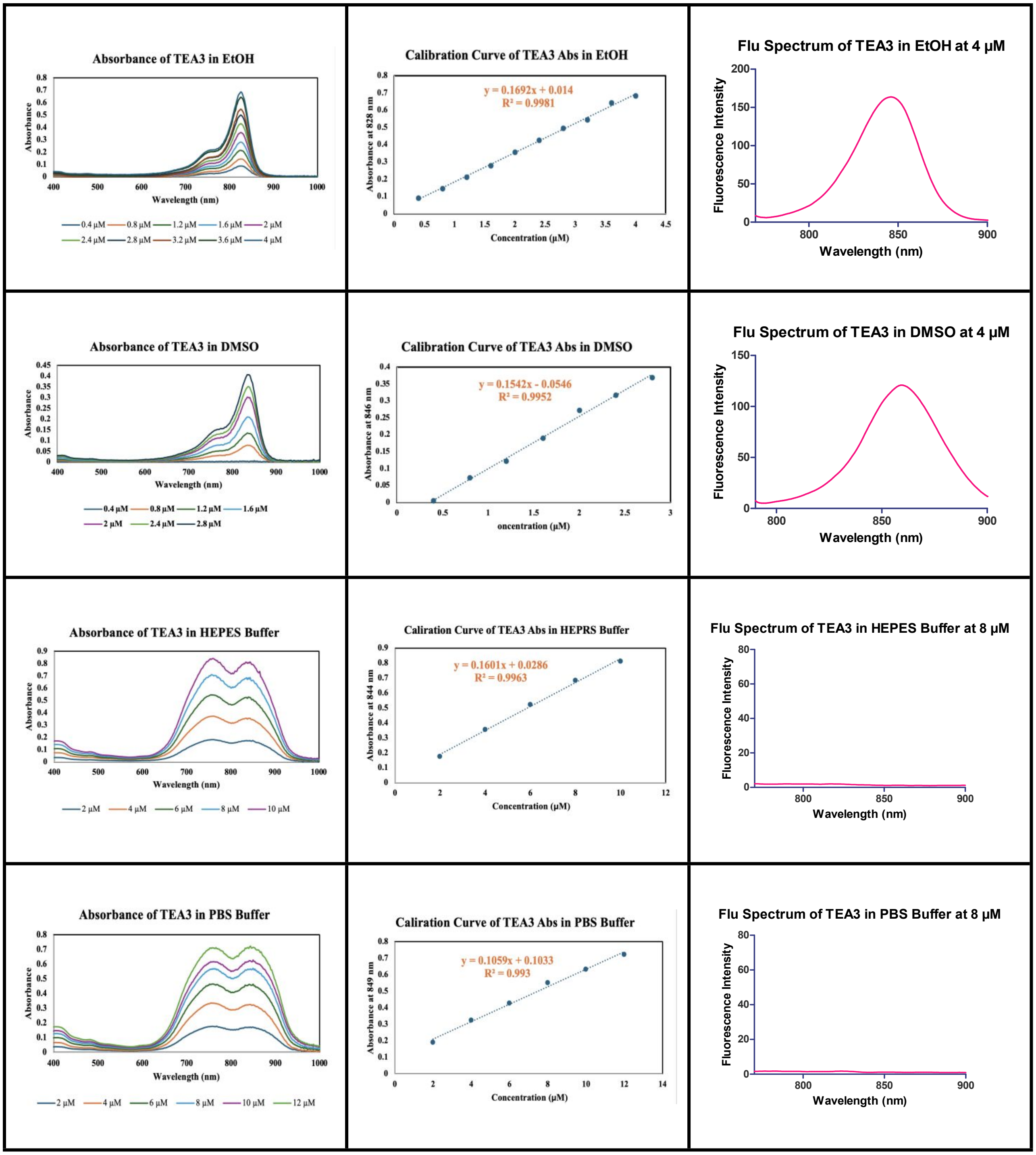

Figure S31. TEA3 Absorbance curves at different concentrations, calibration curves, and fluorescence curves in different solvents

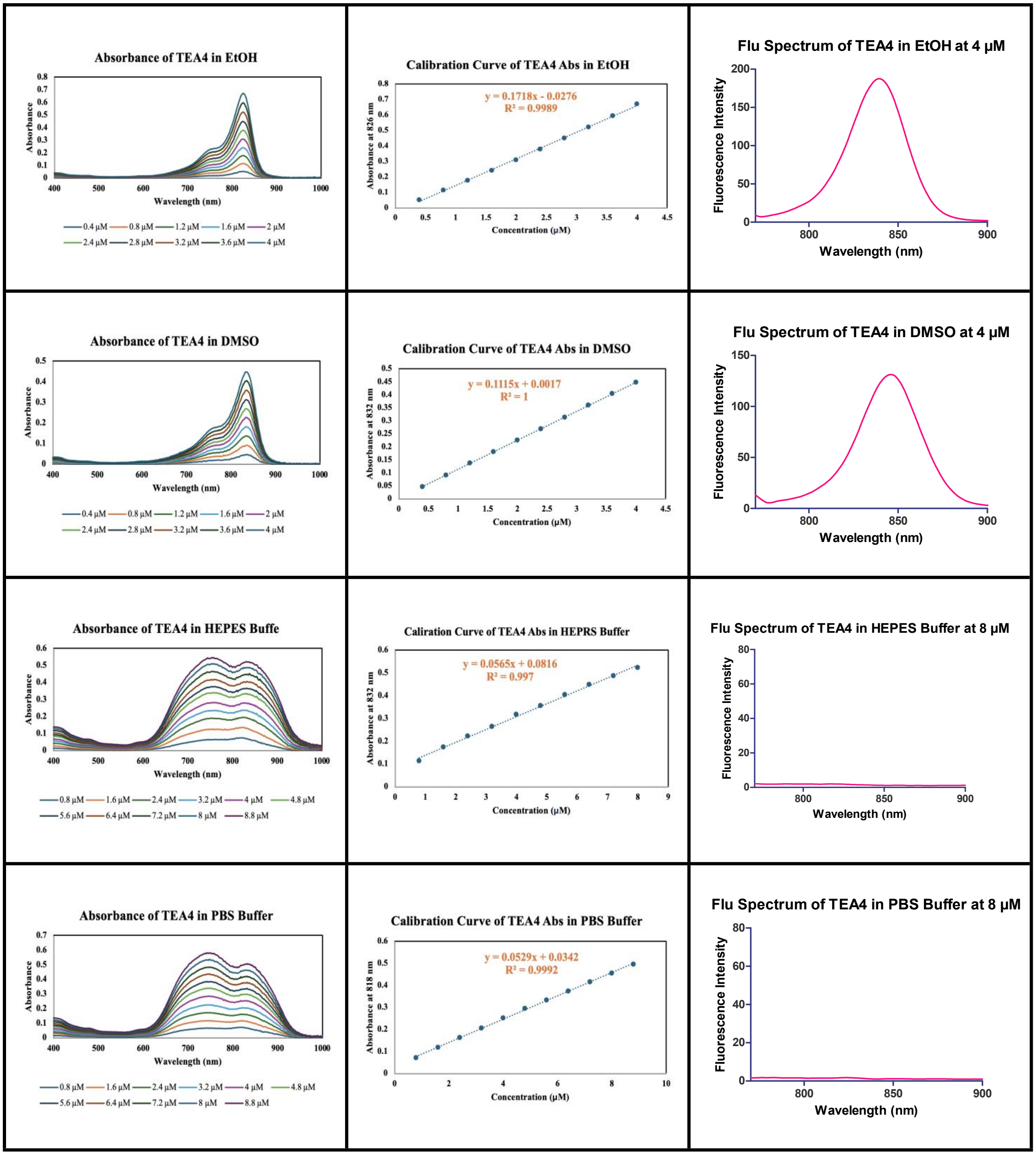

Figure S32. TEA4 Absorbance curves at different concentrations, calibration curves, and fluorescence curves in different solvents

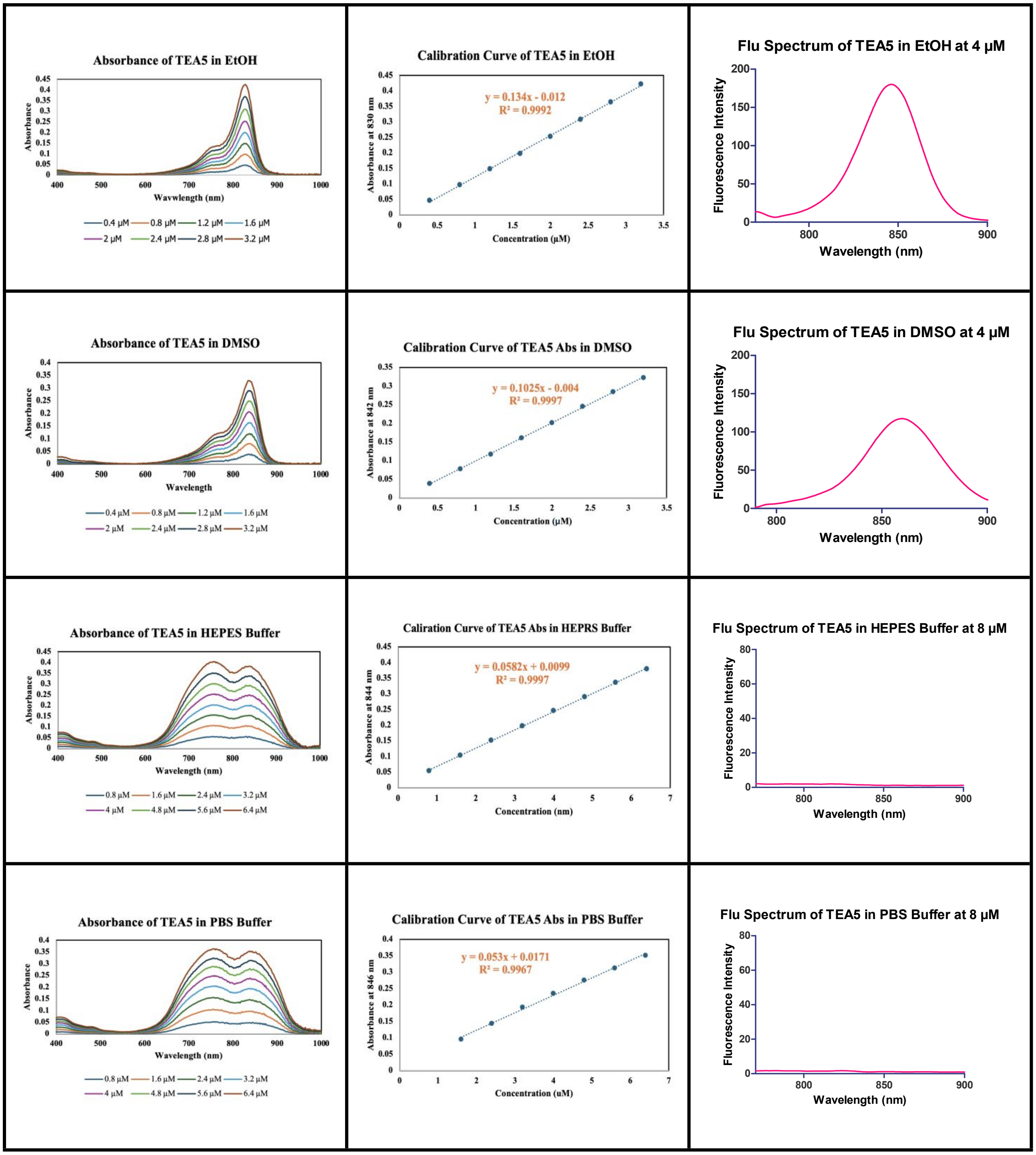

Figure S33. TEA5 Absorbance curves at different concentrations, calibration curves, and fluorescence curves in different solvents

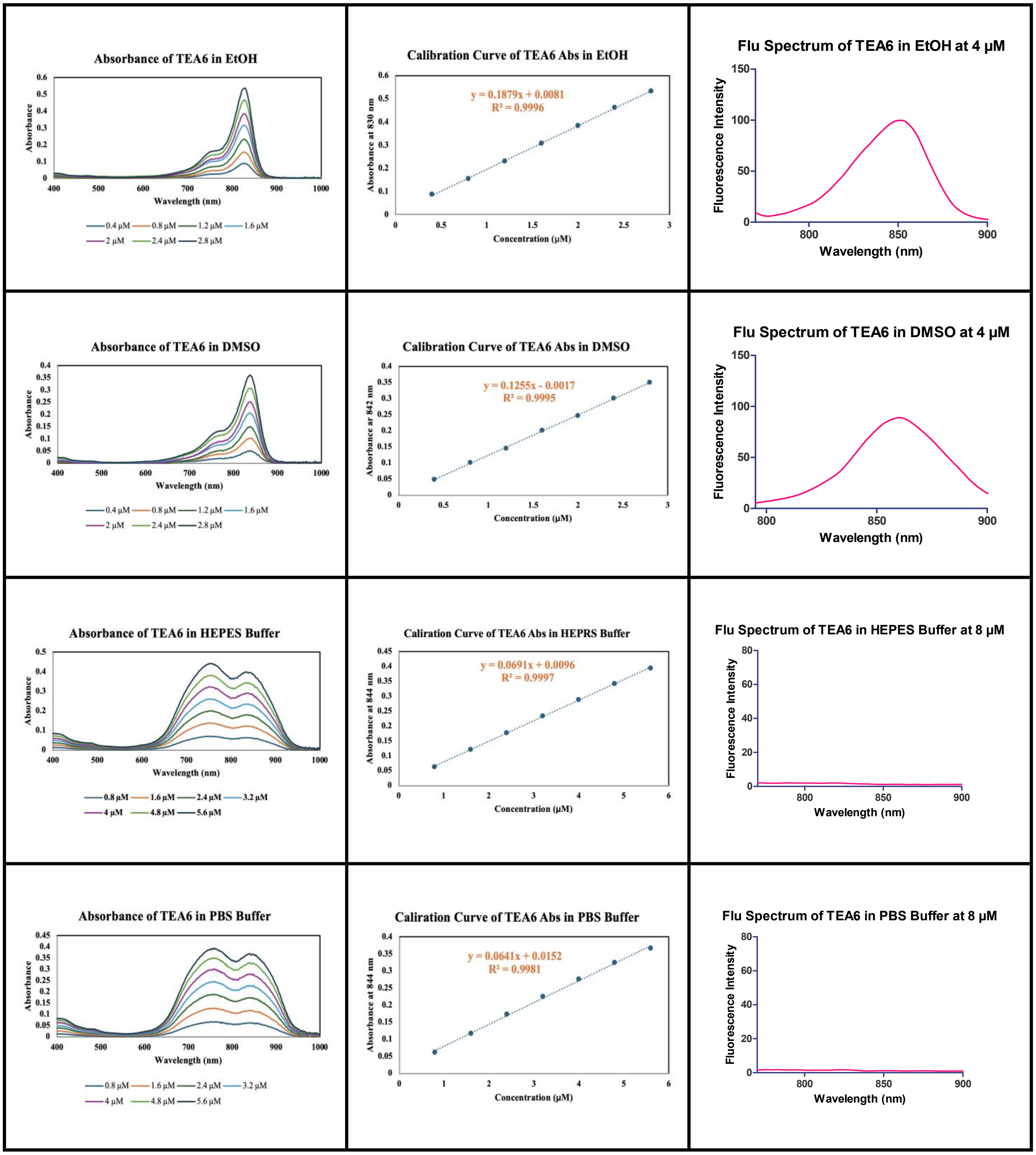

Figure S34. TEA6 Absorbance curves at different concentrations, calibration curves, and fluorescence curves in different solvents

3. Hydrophobicity Studies

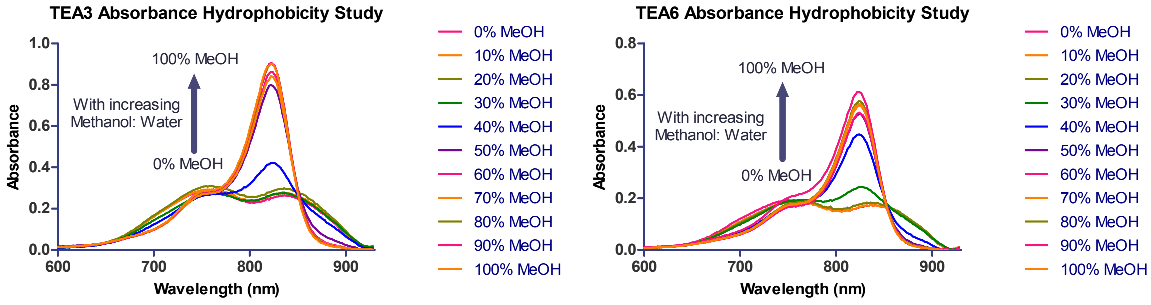

Figure S35. Absorbance spectra of **TEA3, and TEA6** in solutions with increasing methanol: water ratios

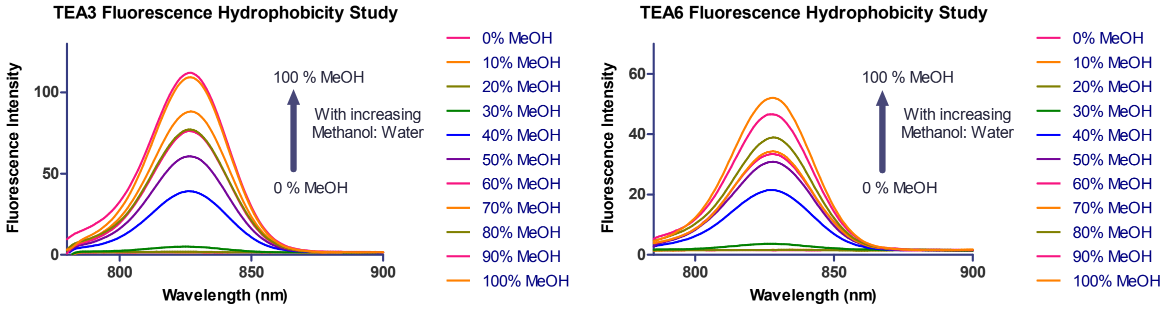

Figure S36. Fluorescence spectra of **TEA3, and TEA6** in solutions with increasing methanol: water ratios

4. DFT Studies for calculation of HOMO and LUMO orbitals

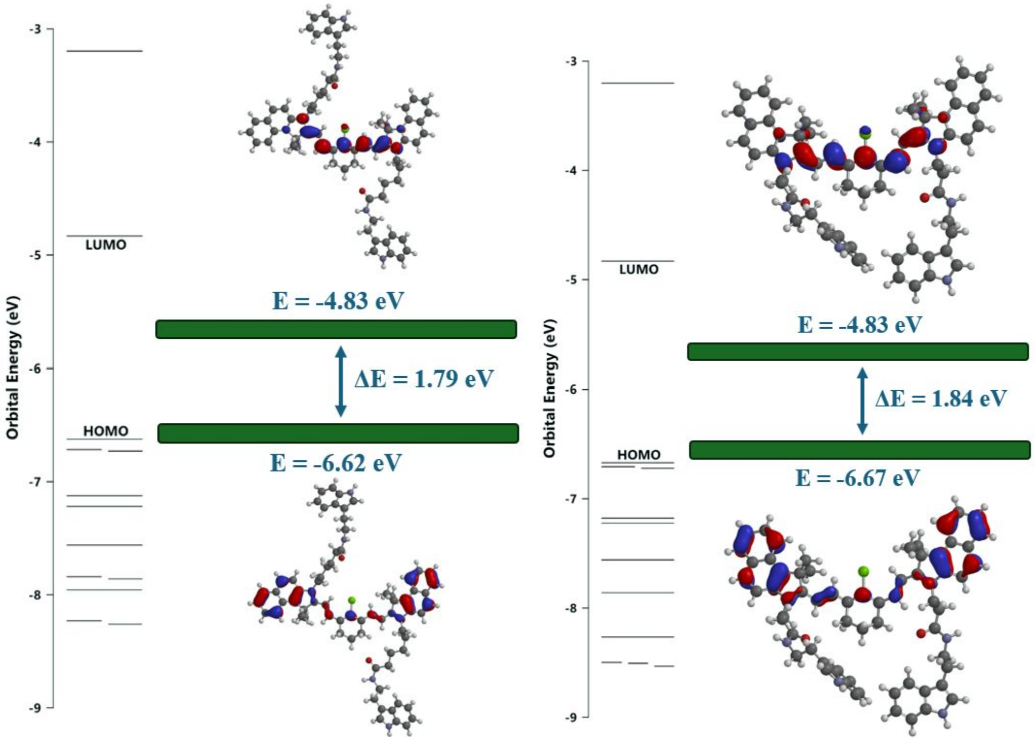

Figure S37. HOMO and LUMO of **TEA3 and TEA6**

5. Overlapped normalized absorbance and fluorescence spectra for determining the wavelength of the 0-0 transition

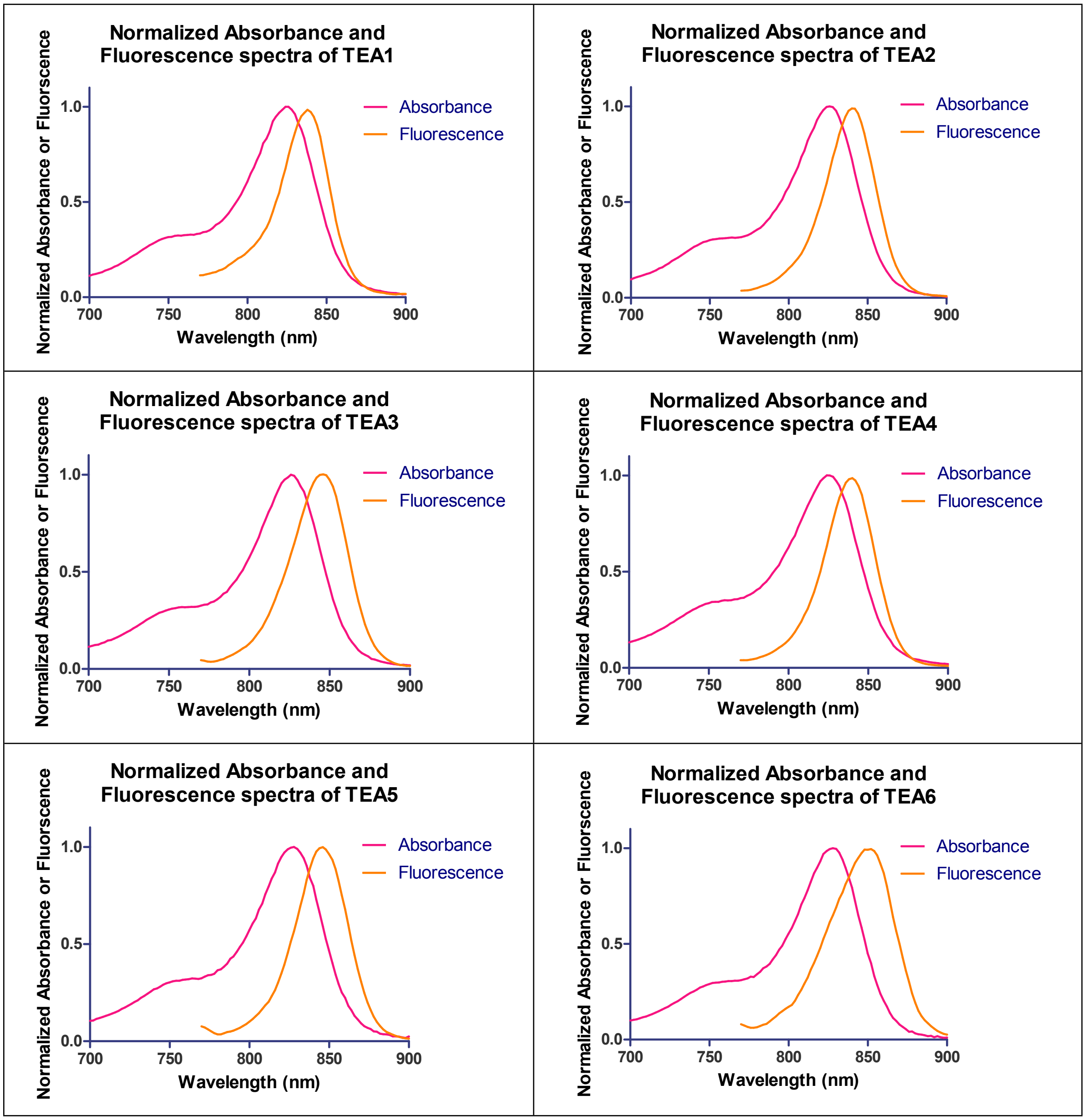

Figure S38. Overlapped normalized absorbance and fluorescence spectra of the peptidomimetic dyes

6. Docking Studies

6.1. Docking figures of the synthesized dyes with bovine serum albumin (BSA)

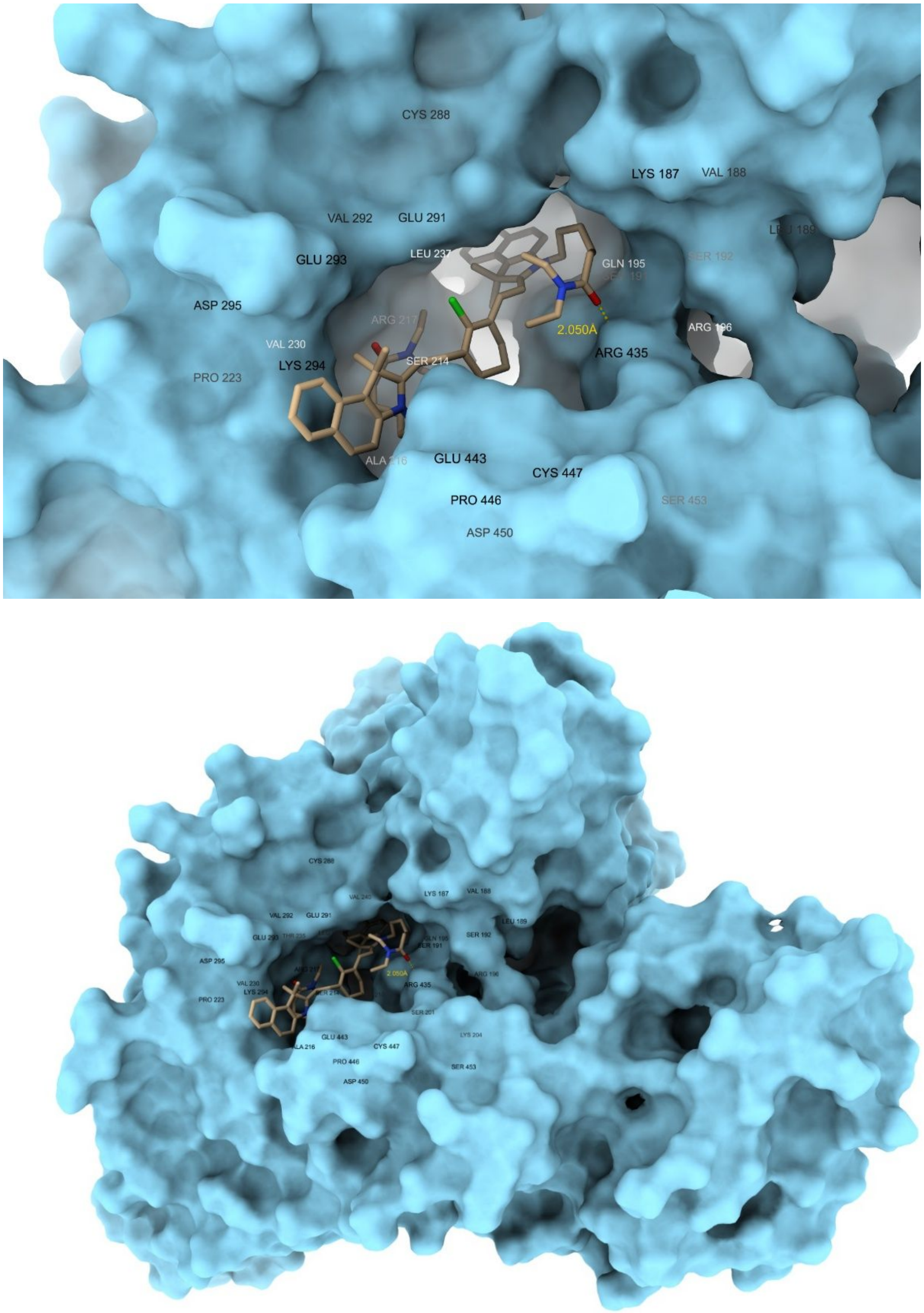

Figure S39. TEA1 highest ranking pose inside bovine serum albumin (BSA, PDB ID: 4jk4)

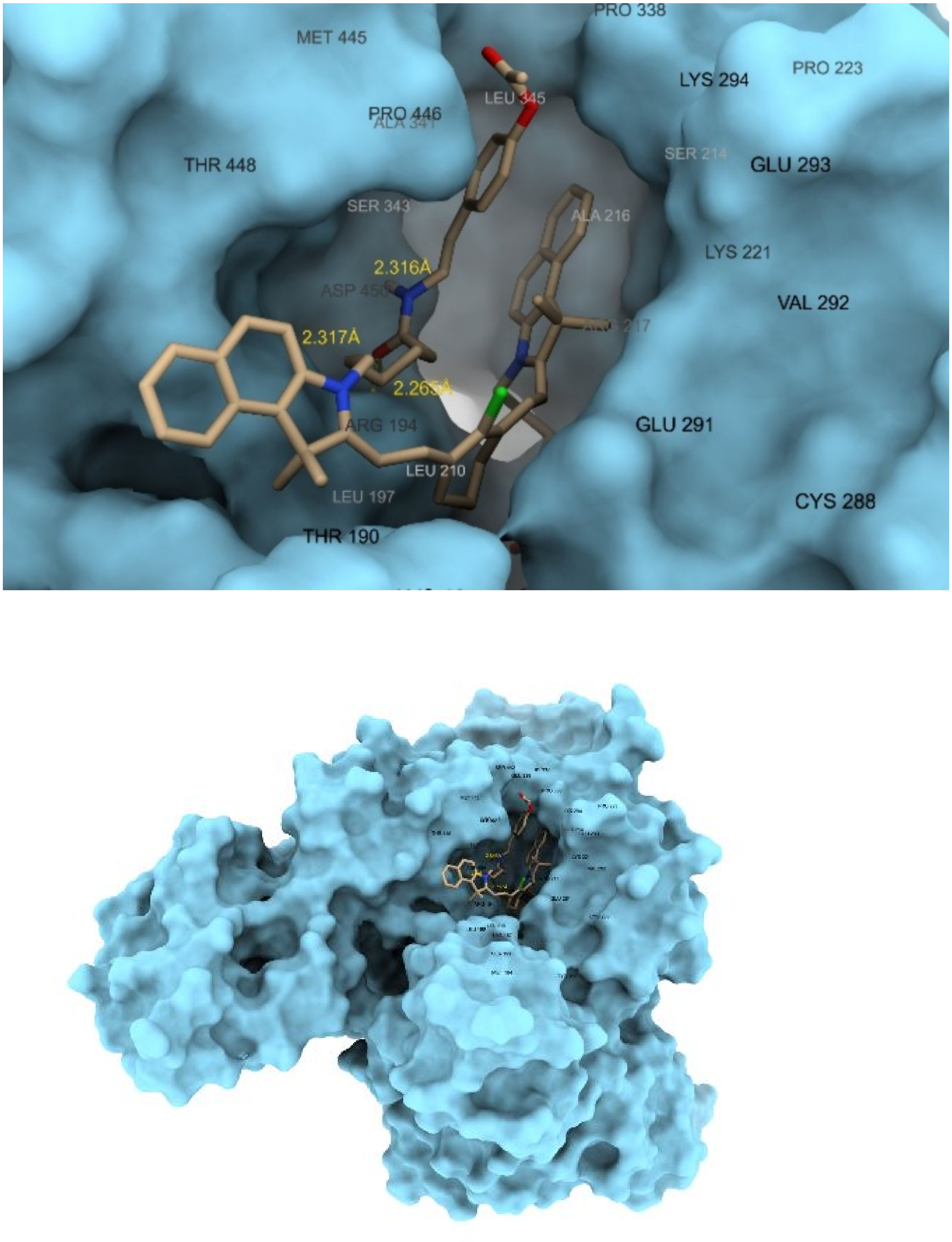

Figure S40. TEA2 highest ranking pose inside bovine serum albumin (BSA, PDB ID: 4jk4)

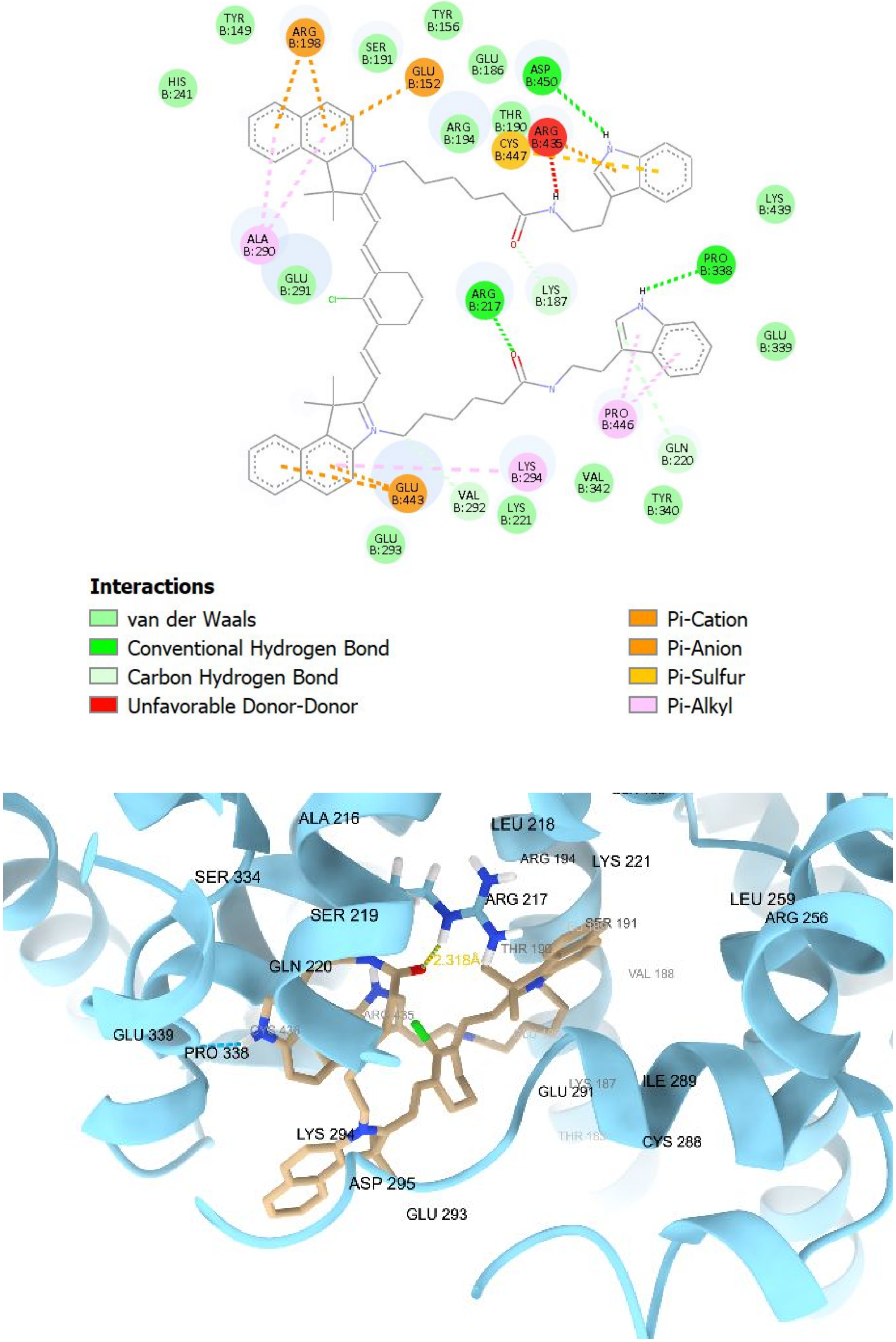

Figure S41. 2D and 3D interaction diagram of **TEA3** with *bovine serum albumin (*BSA*,* PDB ID: 4jk4)

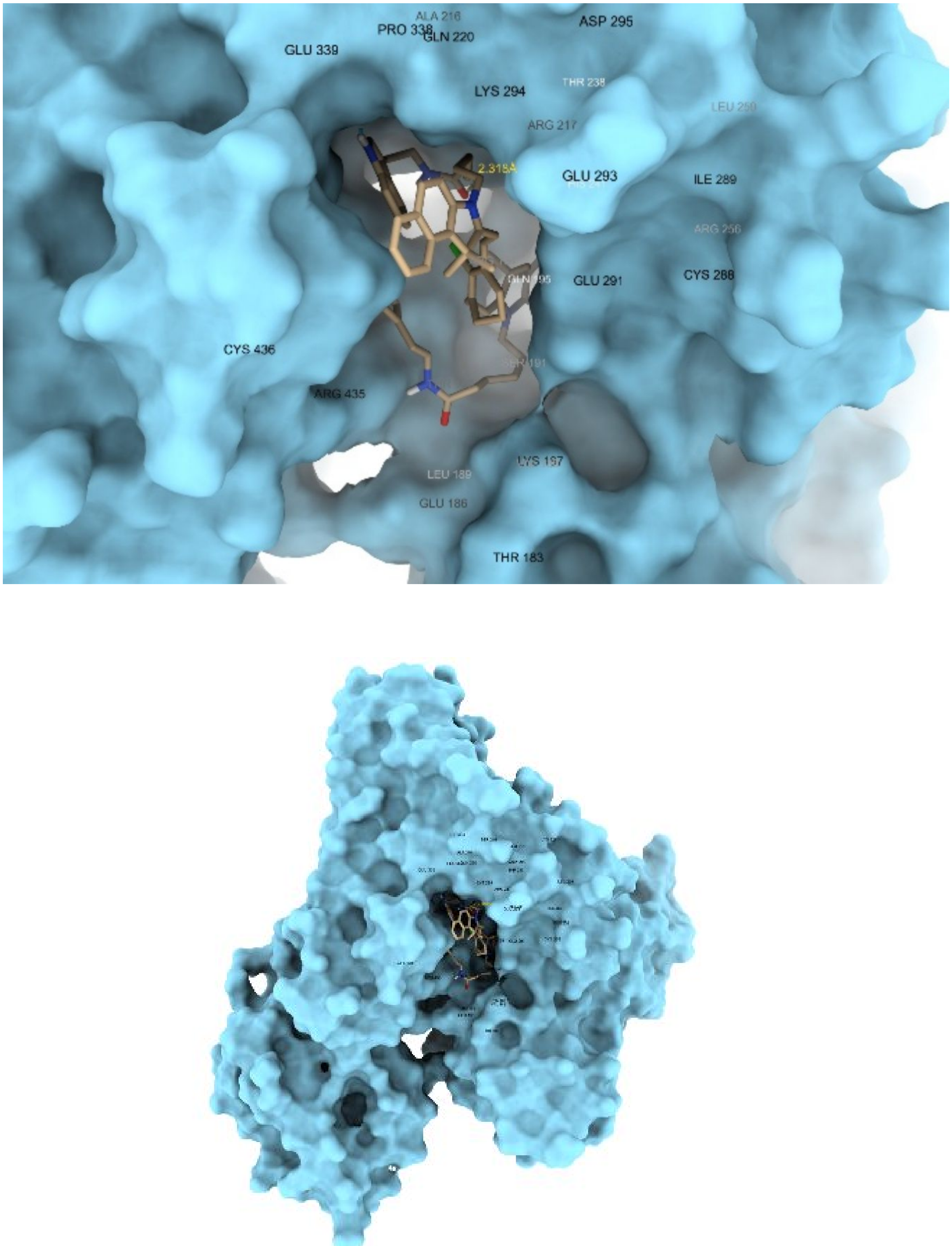

Figure S42. TEA3 highest ranking pose inside bovine serum albumin BSA (BSA, PDB ID: 4jk4)

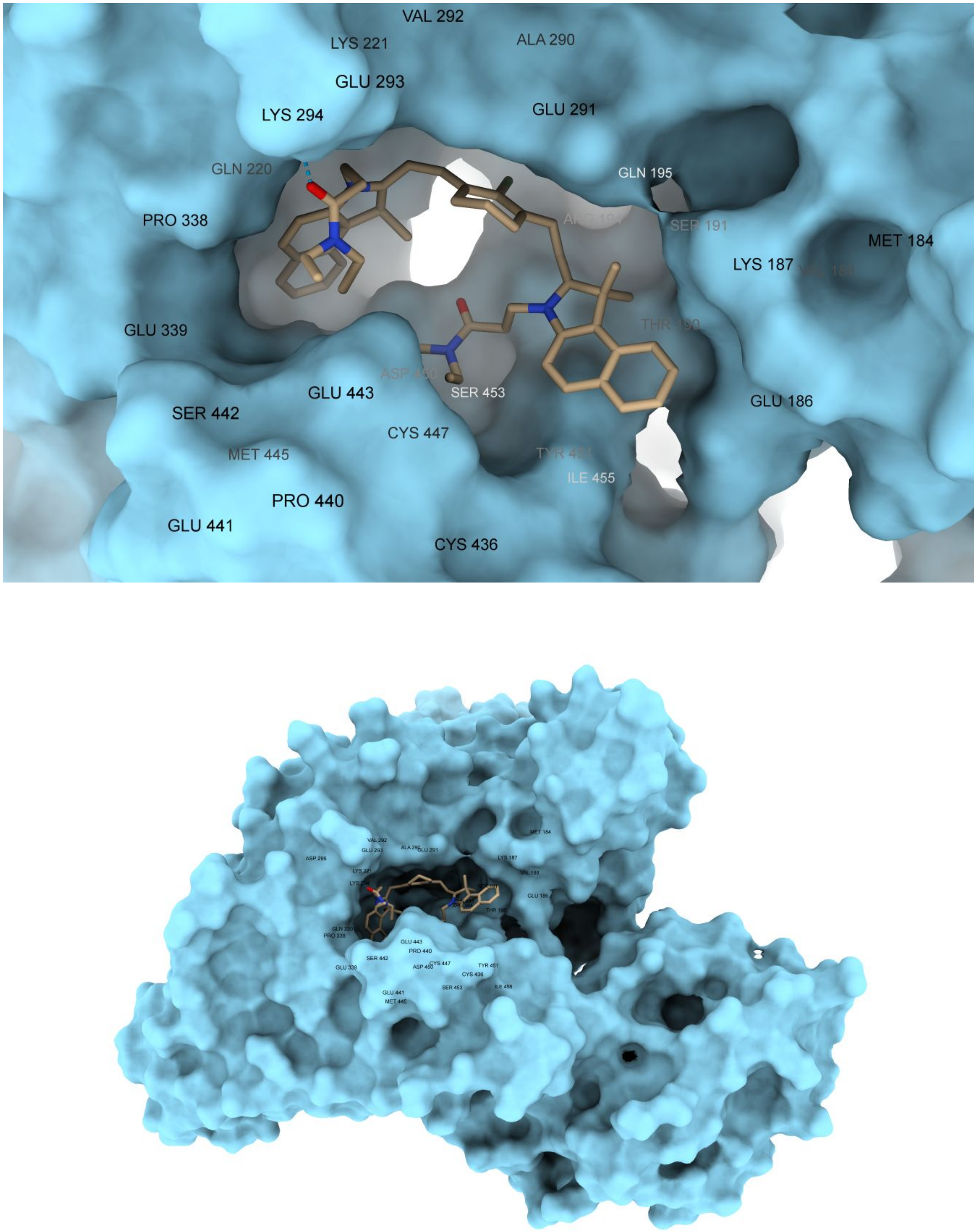

Figure S43. TEA4 highest ranking pose inside bovine serum albumin (BSA, PDB ID: 4jk4)

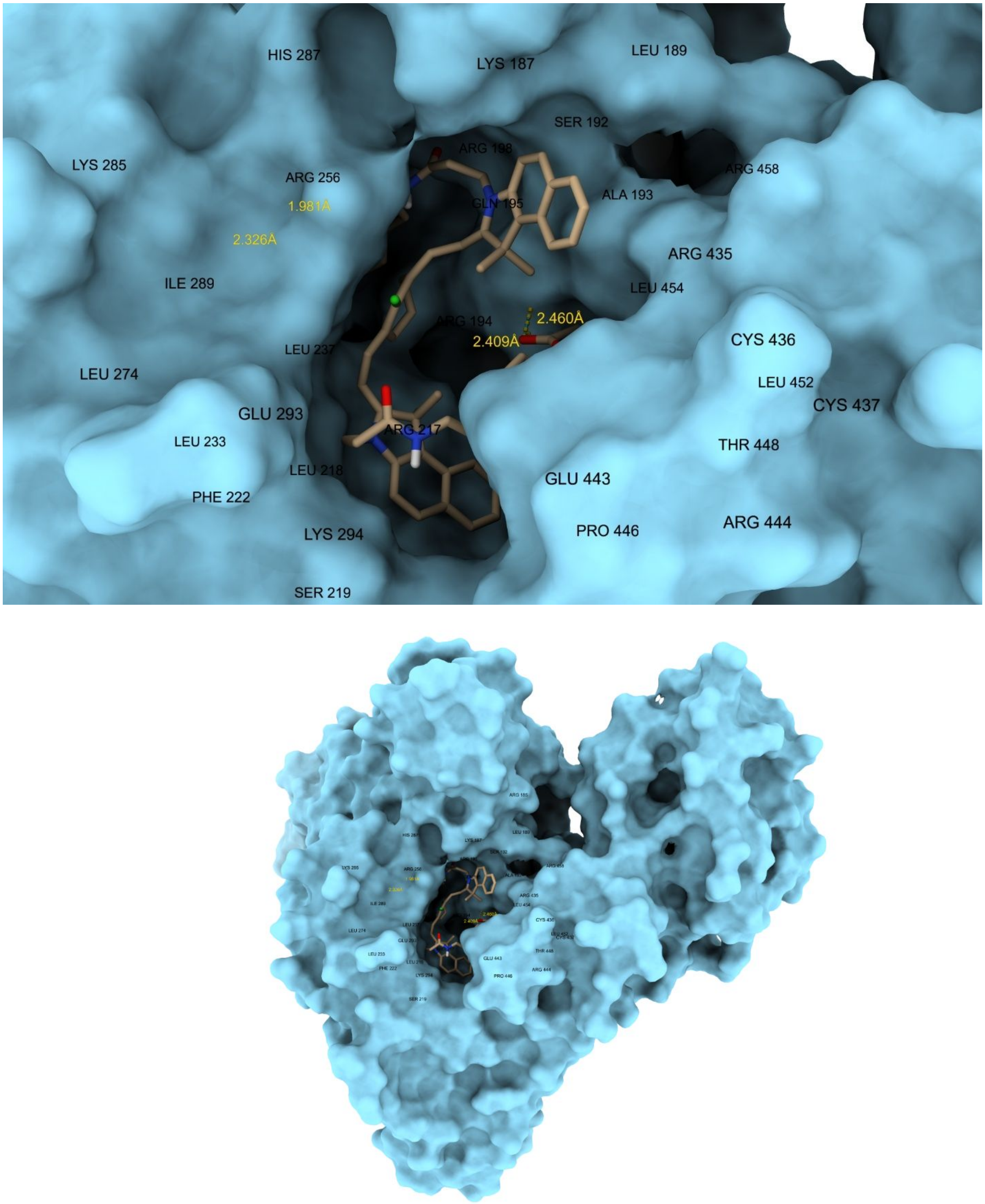

Figure S44. TEA5 highest ranking pose inside bovine serum albumin (BSA, PDB ID: 4jk4)

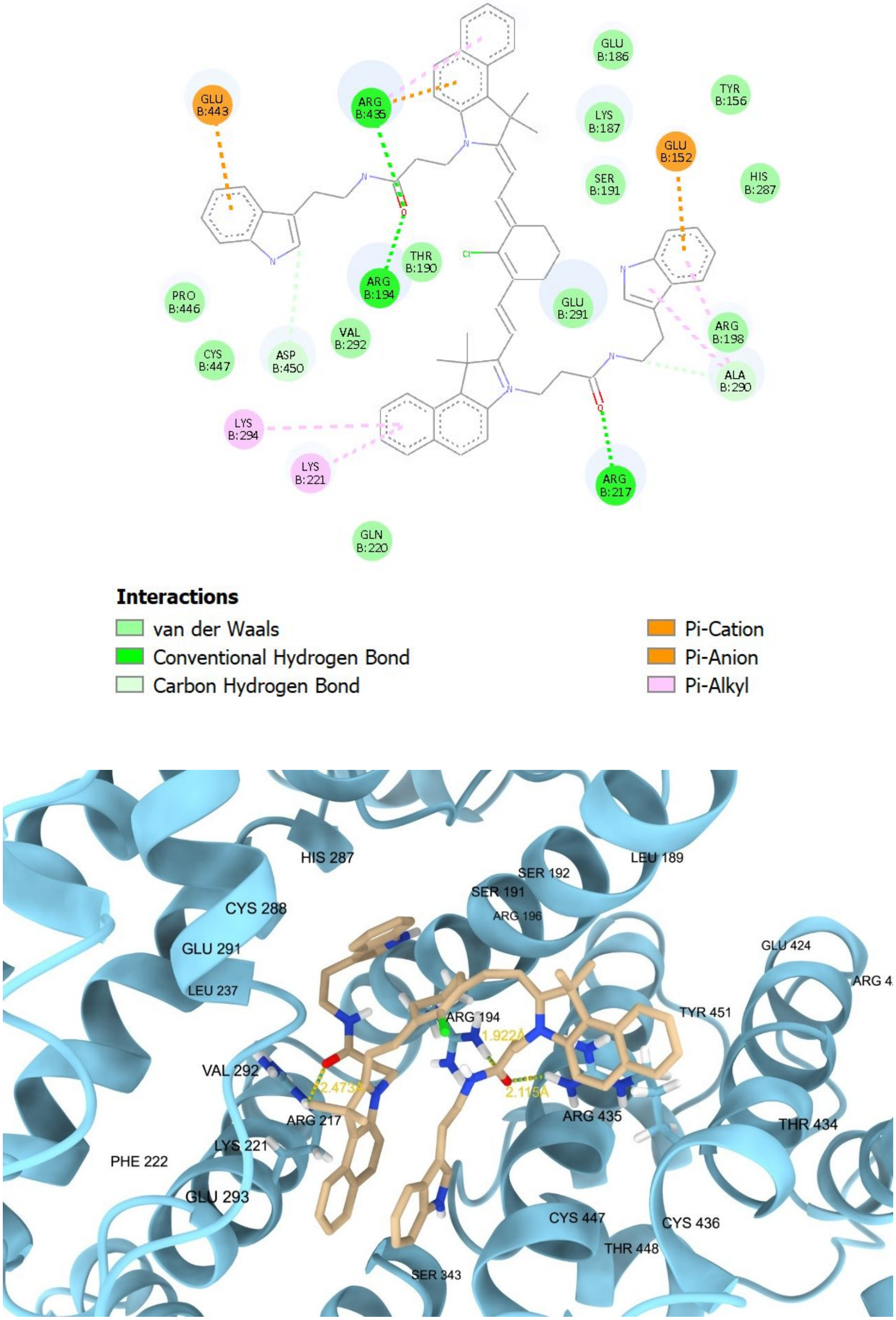

Figure S45. 2D and 3D interaction diagram of **TEA6** with *bovine serum albumin (*BSA*,* PDB ID: 4jk4)

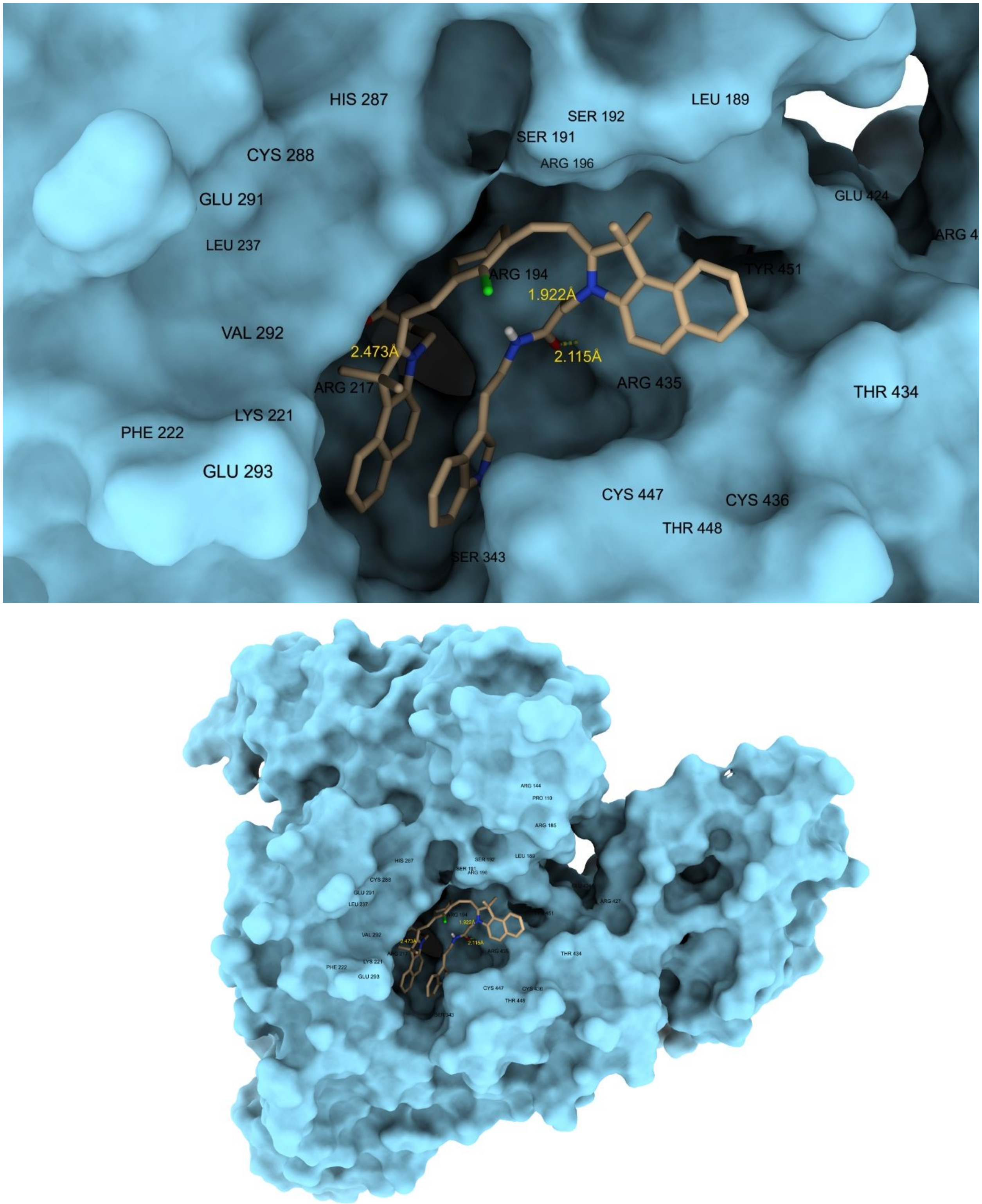

Figure S46. TEA6 highest ranking pose inside bovine serum albumin (BSA, PDB ID: 4jk4)

6.2.**Docking figures of the synthesized dyes with human parvalbumin (HPA)**

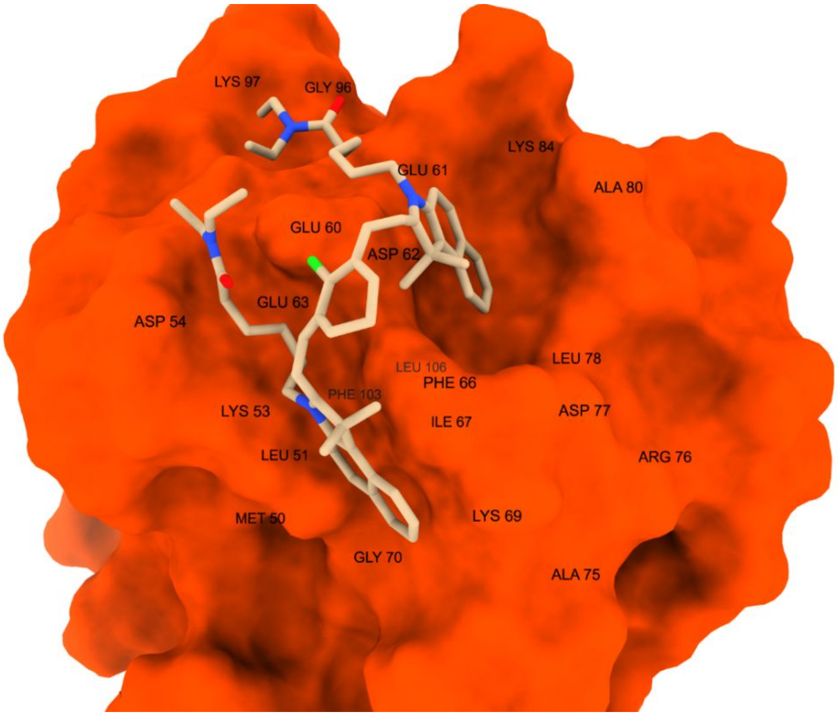

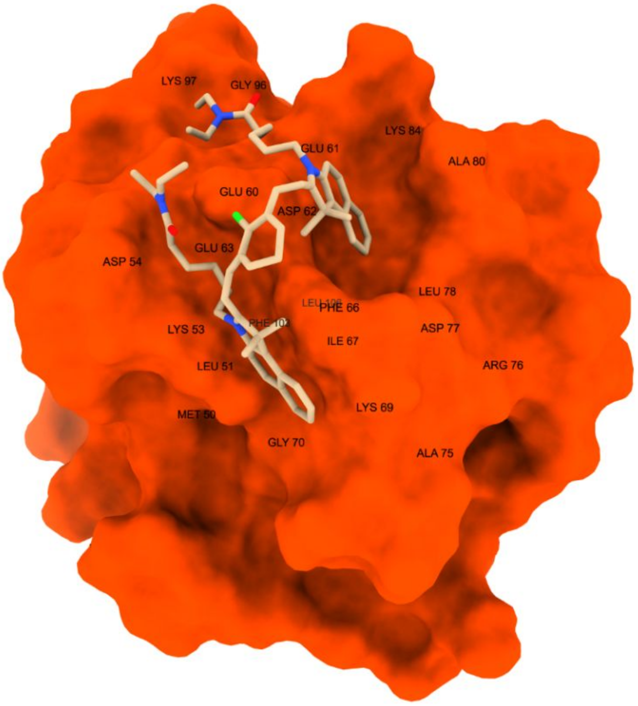

Figure S47. TEA1 highest ranking pose inside human parvalbumin (HPA, PDB ID: 9bb8)

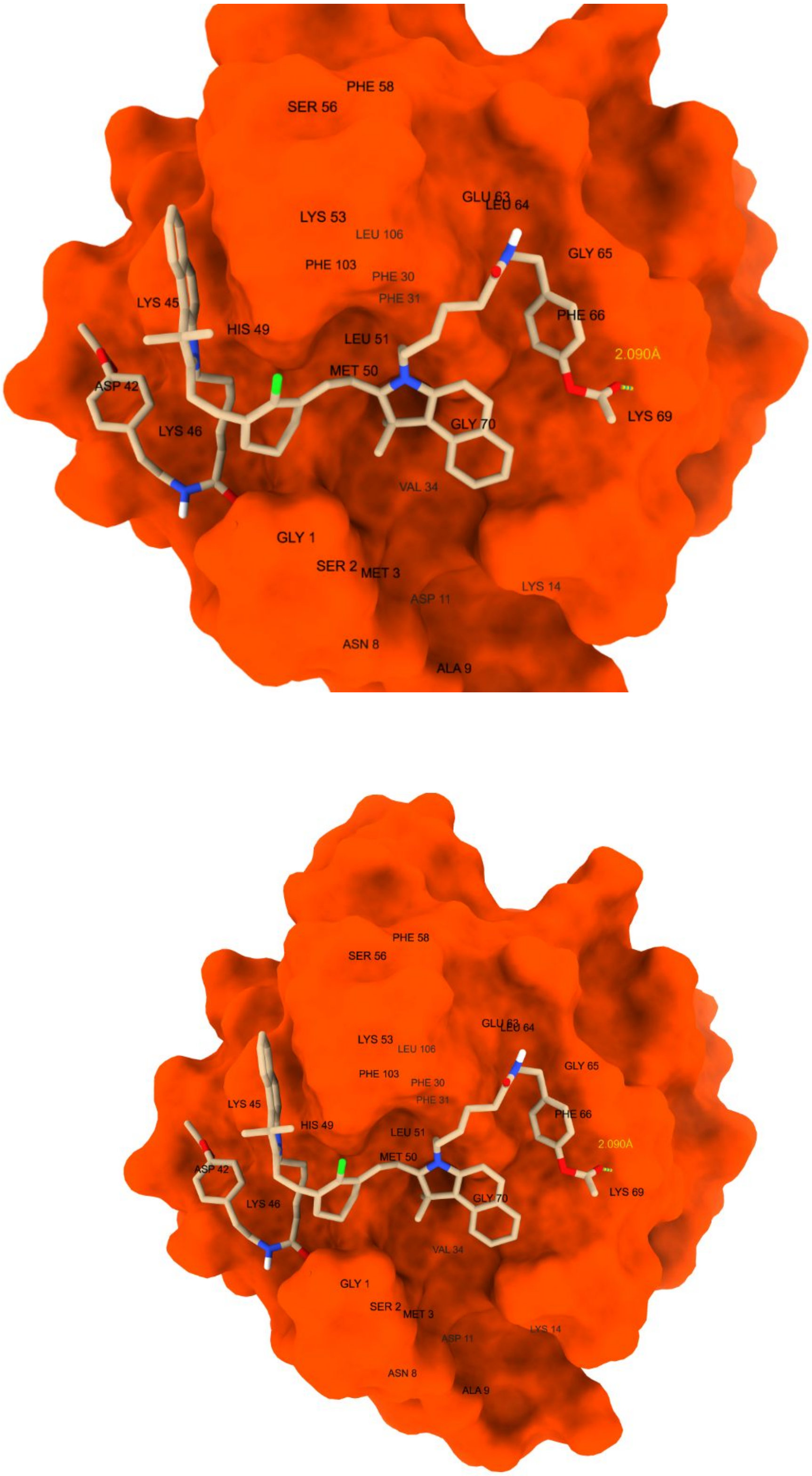

Figure S48. TEA2 highest ranking pose inside human parvalbumin (HPA, PDB ID: 9bb8)

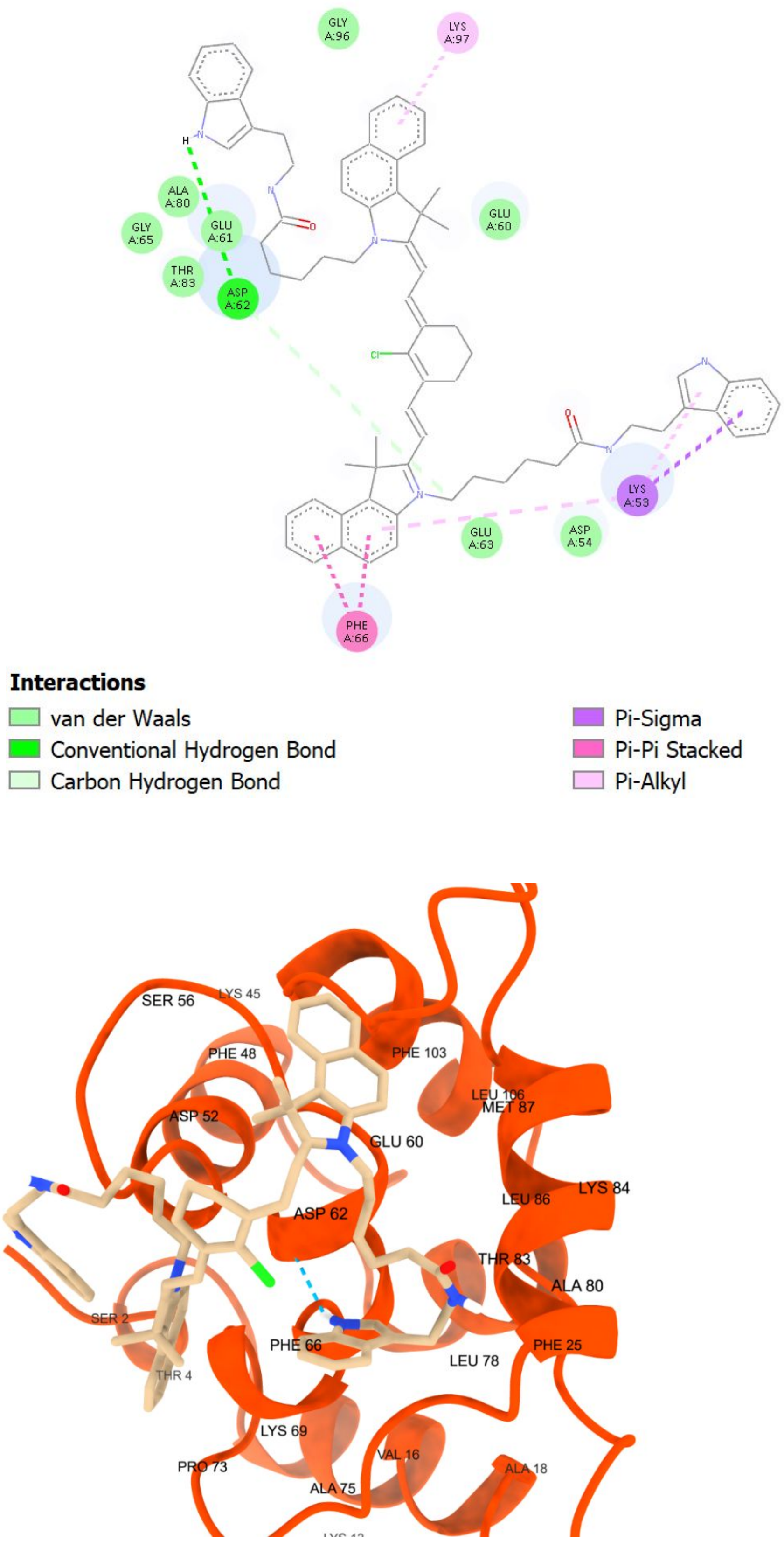

Figure S49. 2D and 3D interaction diagram of **TEA3** with human parvalbumin (HPA, PDB ID: 9bb8)

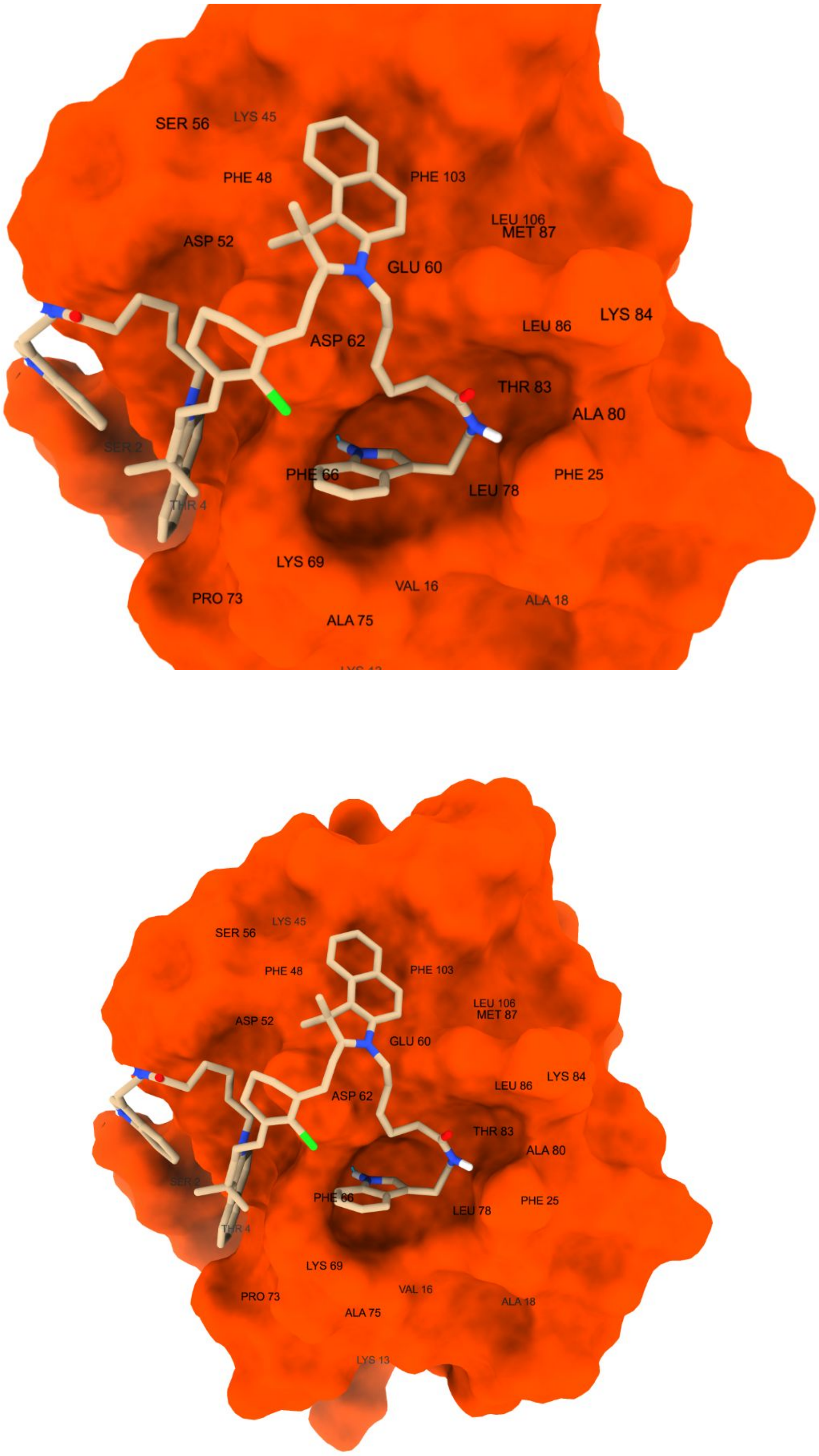

Figure S50. TEA3 highest ranking pose inside human parvalbumin (HPA, PDB ID: 9bb8)

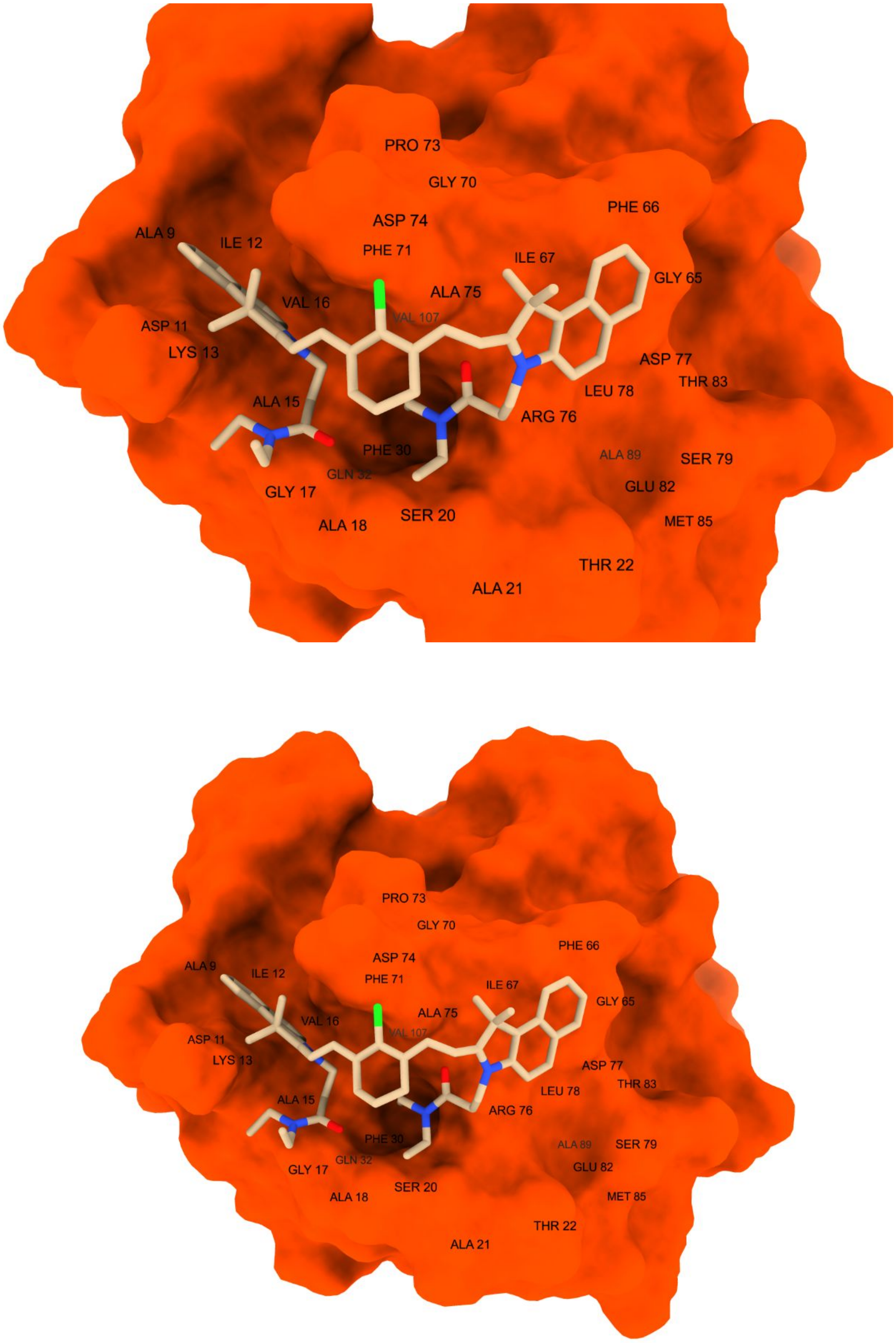

Figure S51. TEA4 highest ranking pose inside human parvalbumin (HPA, PDB ID: 9bb8)

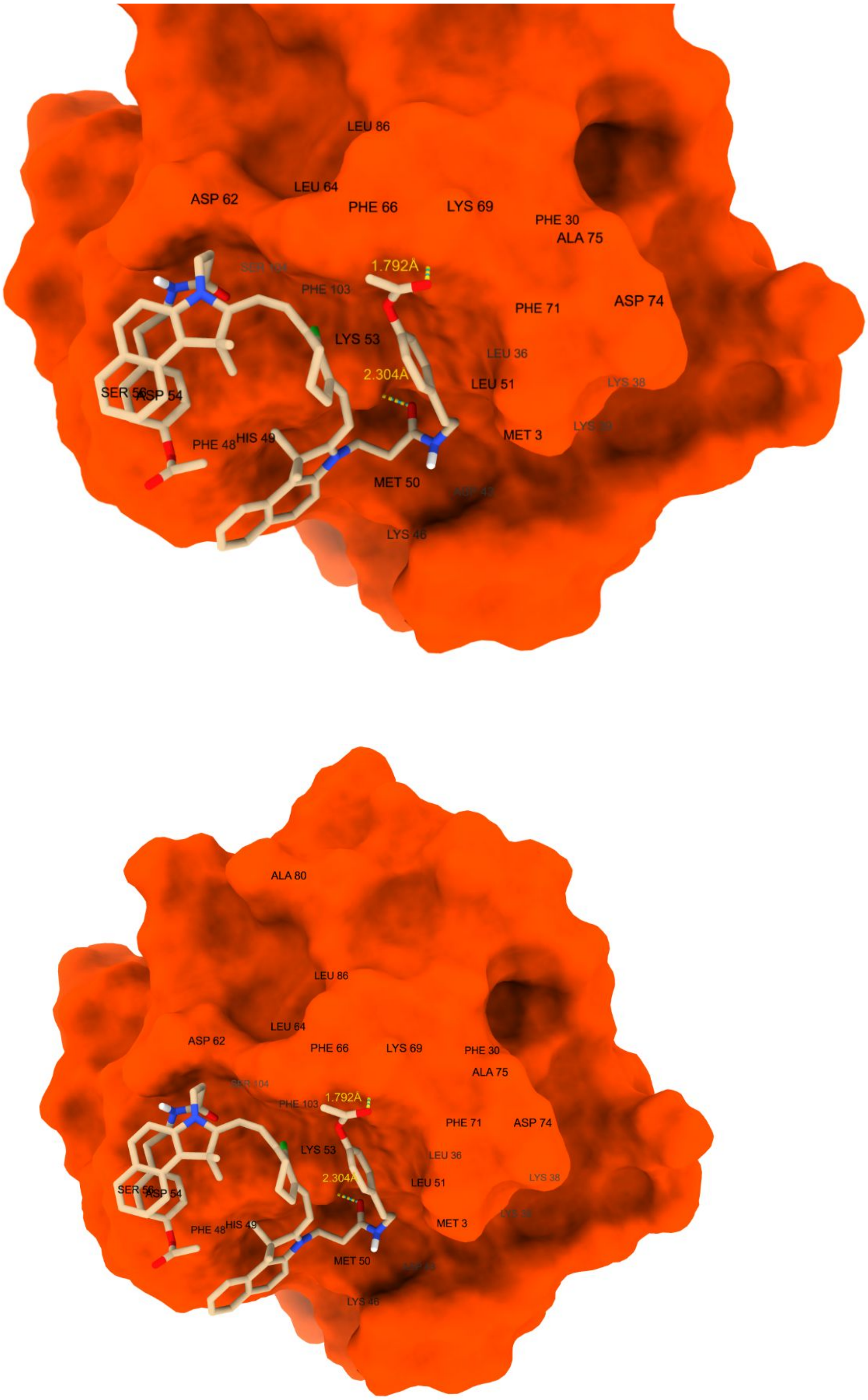

Figure S52. TEA5 highest ranking pose inside human parvalbumin (HPA, PDB ID: 9bb8)

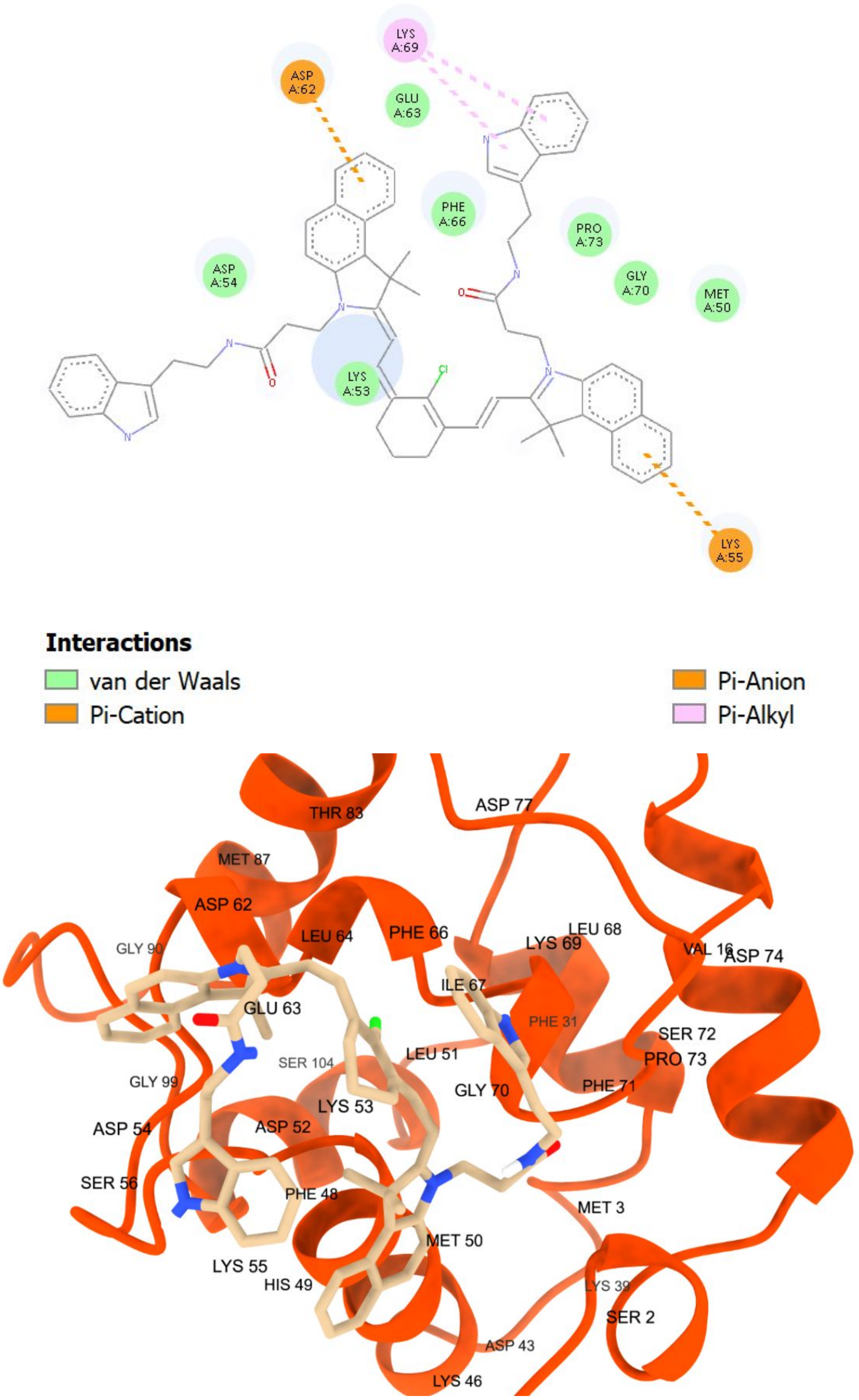

Figure S53. 2D and 3D interaction diagram of **TEA6** with human parvalbumin (HPA, PDB ID: 9bb8)

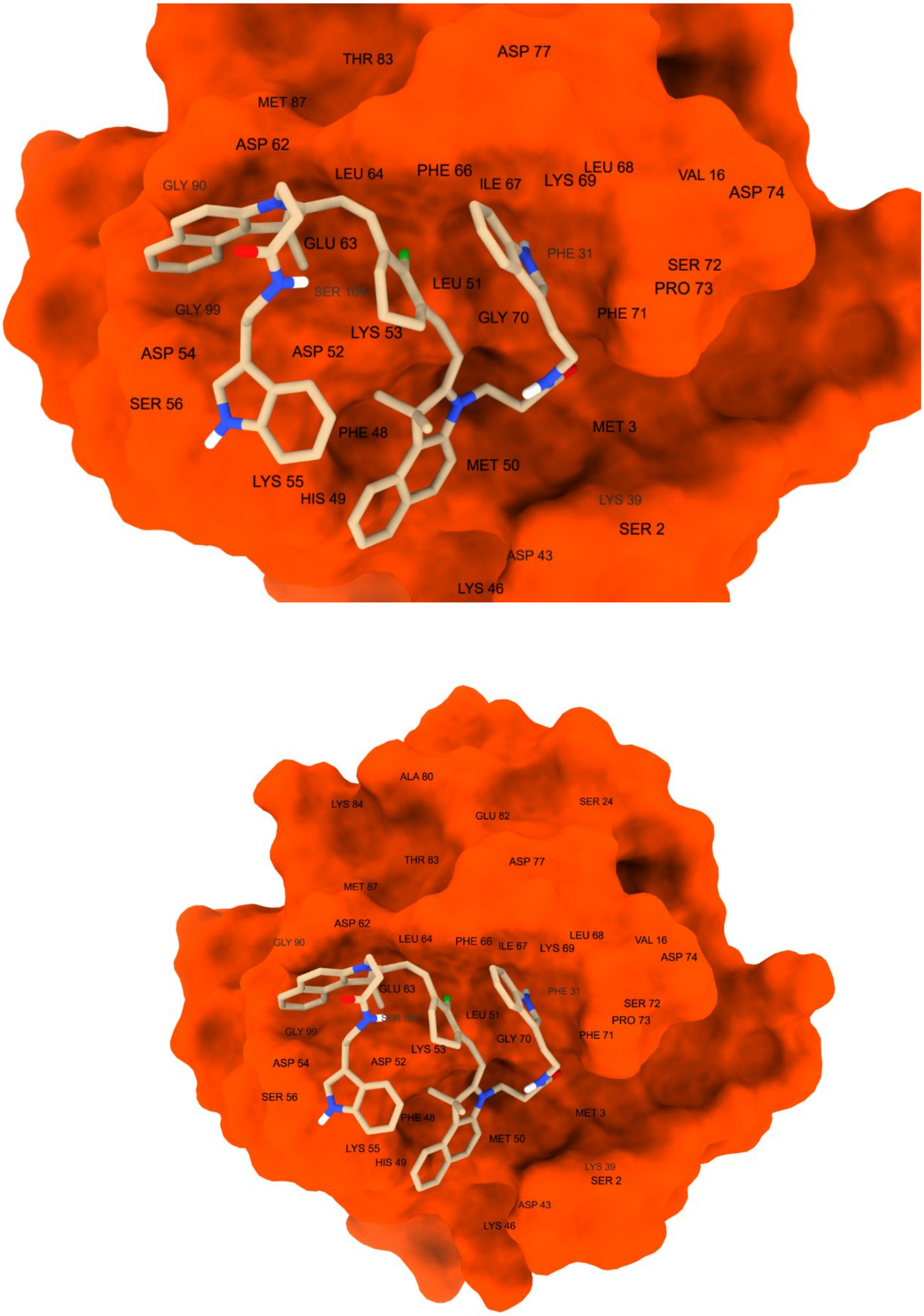

Figure S54. TEA6 highest ranking pose inside human parvalbumin (HPA, PDB ID: 9bb8)

7. Fluorescence enhancement protein binding studies

7.1. Kinetic Analysis

Table S1. Kinetic analysis parameters of fluorophores binding with BSA

Fluorophore	Parameter	Trial 1	Trial 2	Trial 3	Average
**TEA1**	Rate constant K (s^{-1})	0.00268	0.00255	0.00232	**0.00252**
	Half-Life $T_{1/2}$ (s)	200.7	272.3	298.6	**257.2**
**TEA2**	Rate constant K (s^{-1})	0.00092	0.00100	0.00087	**0.00093**
	Half-Life $T_{1/2}$ (s)	756.8	691.5	798.2	**748.8**
**TEA3**	Rate constant K (s^{-1})	-	-	-	**-**
	Half-Life $T_{1/2}$ (s)	-	-	-	**-**
**TEA4**	Rate constant K (s^{-1})	Too fast	Too fast	Too fast	**TFTM***
	Half-Life $T_{1/2}$ (s)	Too fast	Too fast	Too fast	**TFTM***
**TEA5**	Rate constant K (s^{-1})	0.00094	0.00101	0.00098	**0.00098**
	Half-Life $T_{1/2}$ (s)	737.1	689.6	705.6	**710.8**
**TEA6**	Rate constant K (s^{-1})	-	-	-	**-**
	Half-Life $T_{1/2}$ (s)	-	-	-	**-**

TFTM*: Too fast to be measured

Table S2. Kinetic analysis parameters of fluorophores binding with HPA

Fluorophore	Parameter	Trial 1	Trial 2	Trial 3	Average
**TEA1**	Rate constant K (s^{-1})	0.000854	0.000940	0.001026	**0.00094**
	Half-Life $T_{1/2}$ (s)	811.0	737.3	675.6	**741.3**
**TEA2**	Rate constant K (s^{-1})	0.0004219	0.000451388	0.000385568	**0.00042**
	Half-Life $T_{1/2}$ (s)	1643	1532	1760	**1645**
**TEA3**	Rate constant K (s^{-1})	-	-	-	**-**
	Half-Life $T_{1/2}$ (s)	-	-	-	**-**
**TEA4**	Rate constant K (s^{-1})	Too fast	Too fast	Too fast	**TFTM***
	Half-Life $T_{1/2}$ (s)	Too fast	Too fast	Too fast	**TFTM***
**TEA5**	Rate constant K (s^{-1})	0.000502588	0.000461751	0.000490792	**0.00049**
	Half-Life $T_{1/2}$ (s)	1378	1501	1412	**1430**
**TEA6**	Rate constant K (s^{-1})	-	-	-	**-**
	Half-Life $T_{1/2}$ (s)	-	-	-	**-**

TFTM*: Too fast to be measured

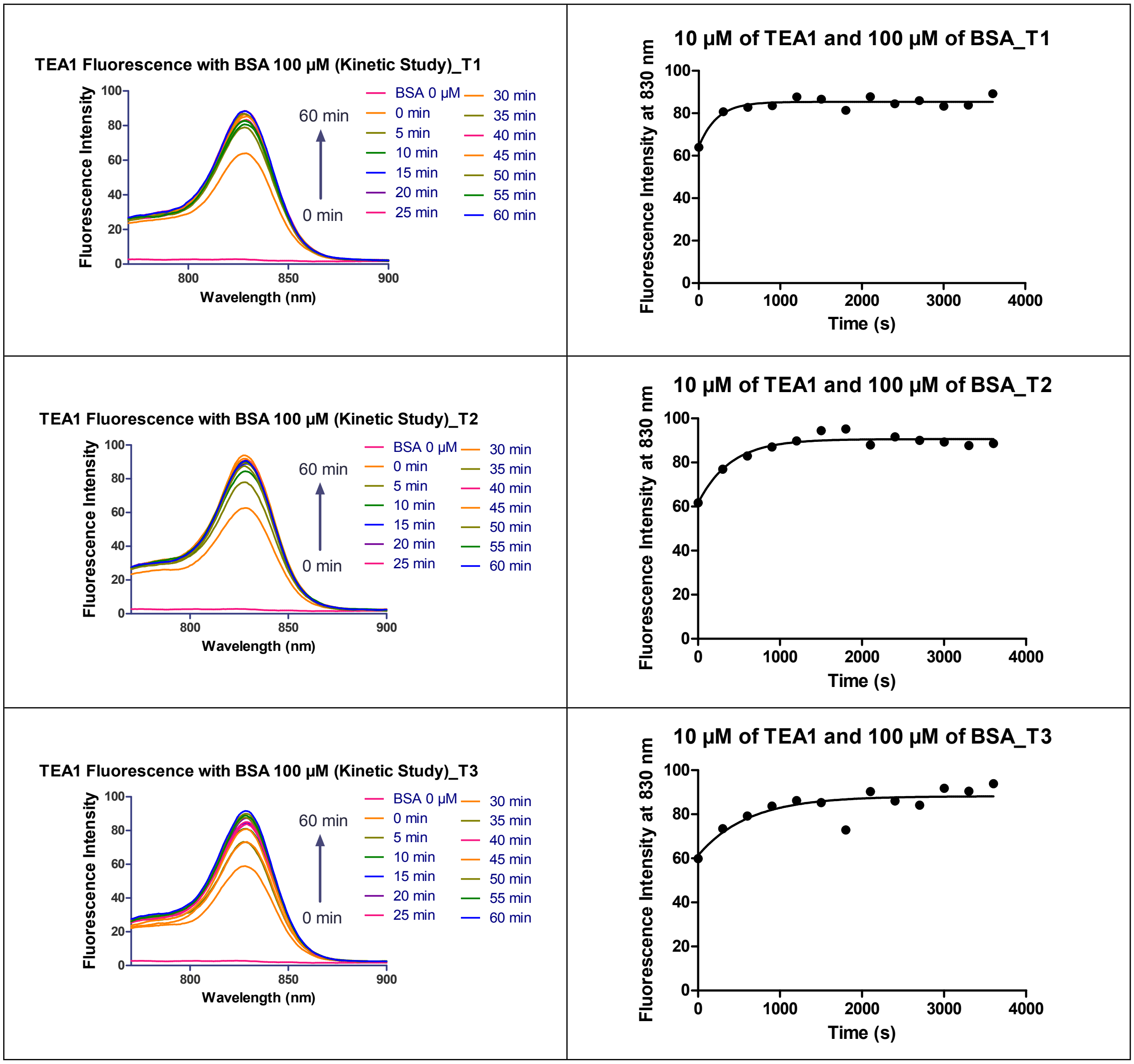

Figure S55. Fluorescence spectra of **TEA1** in PBS in the presence of 100 μM of **bovine serum albumin (BSA)** during a time interval of 60 minutes and plotting of the change in fluorescence with time. Each experiment was repeated 3 times (T1-T3)

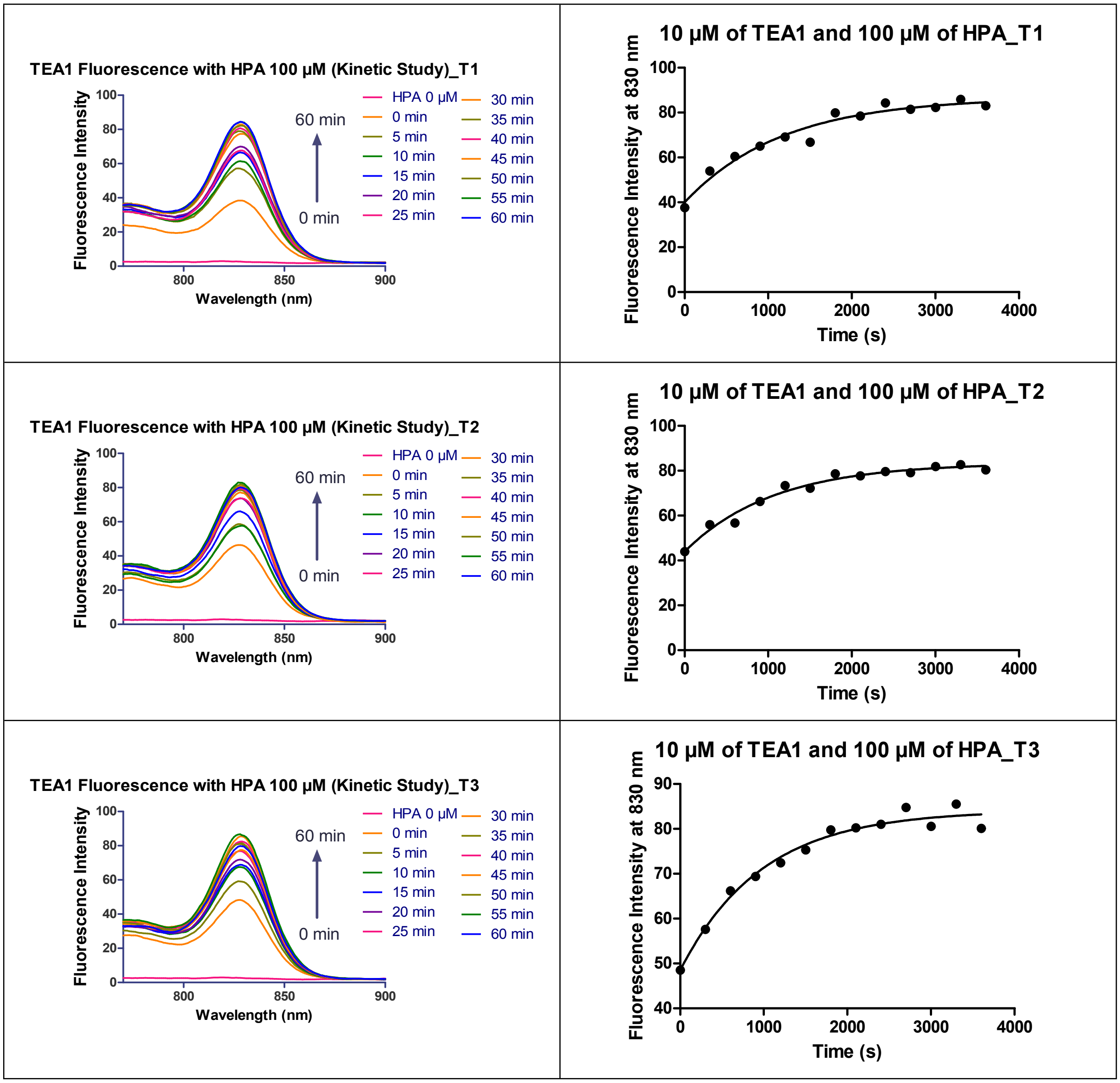

Figure S56. Fluorescence spectra of **TEA1** in PBS in the presence of 100 μM of **human parvalbumin (HPA)** during a time interval of 60 minutes and plotting of the change in fluorescence with time. Each experiment was repeated 3 times (T1-T3)

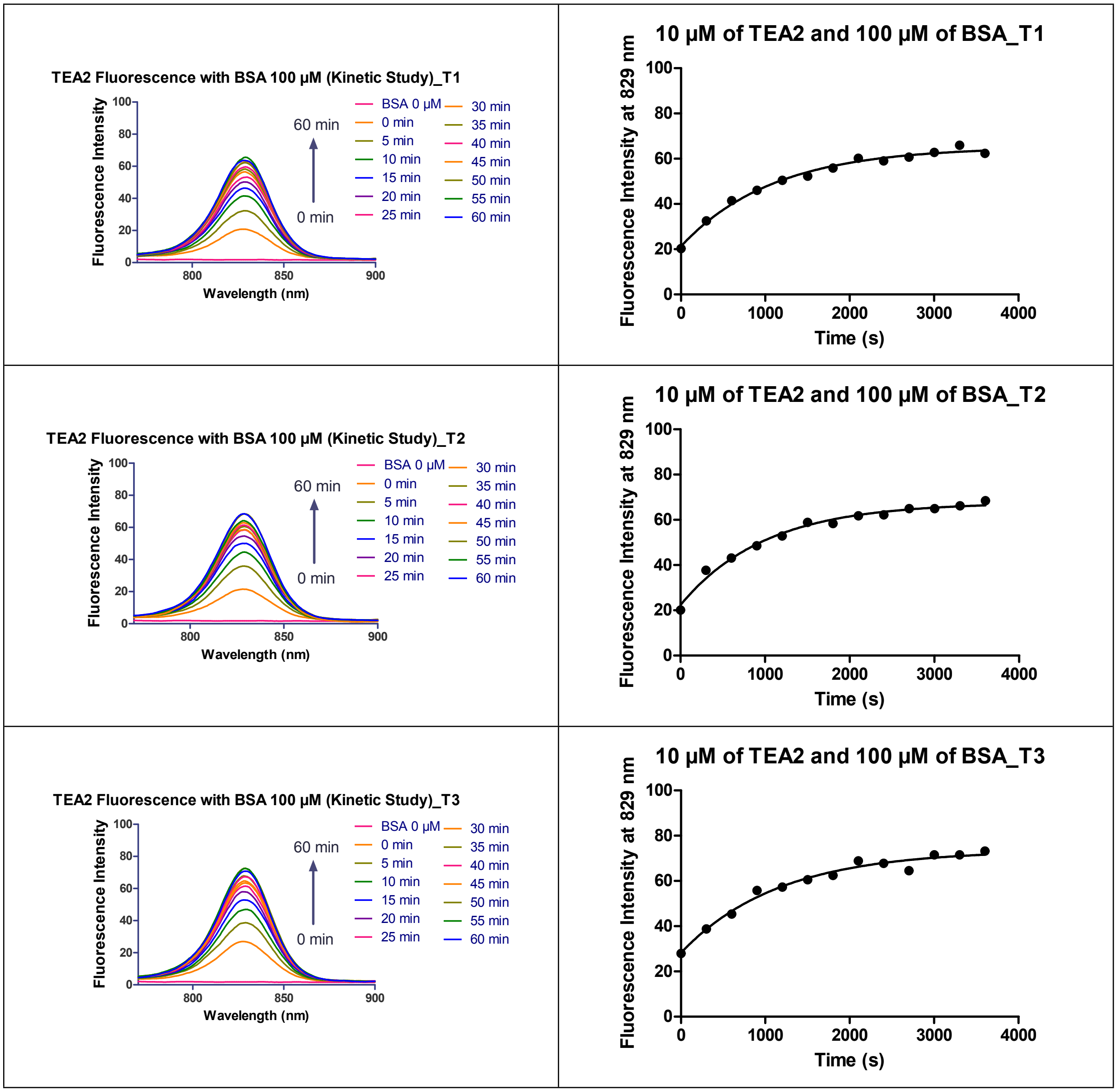

Figure S57. Fluorescence spectra of **TEA2** in PBS in the presence of 100 µM of **bovine serum albumin (BSA)** or human parvalbumin (HPA) during a time interval of 60 minutes and plotting of the change in fluorescence with time. Each experiment was repeated 3 times (T1-T3)

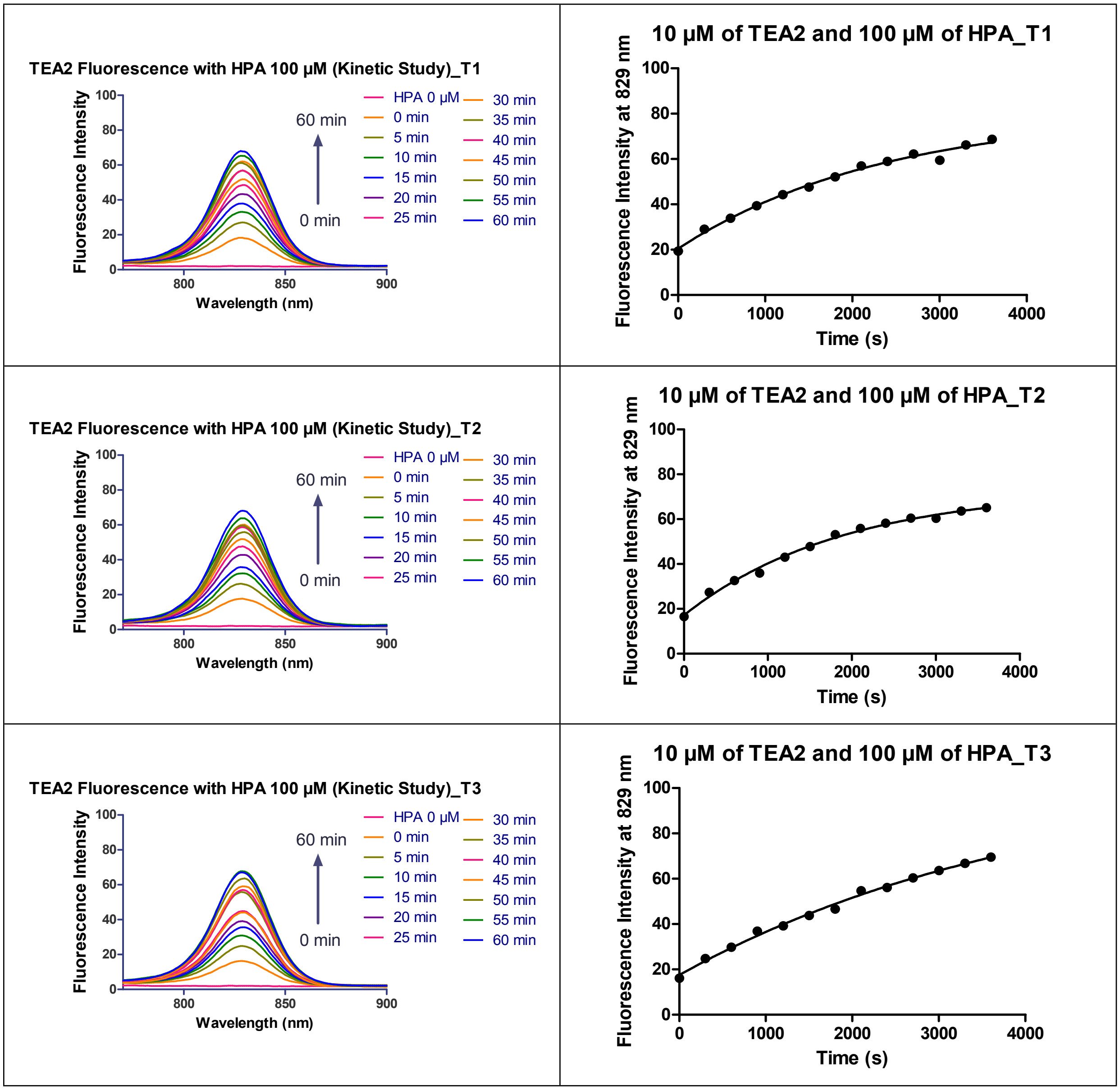

Figure S58. Fluorescence spectra of **TEA2** in PBS in the presence of 100 µM of **human parvalbumin (HPA)** during a time interval of 60 minutes and plotting of the change in fluorescence with time. Each experiment was repeated 3 times (T1-T3)

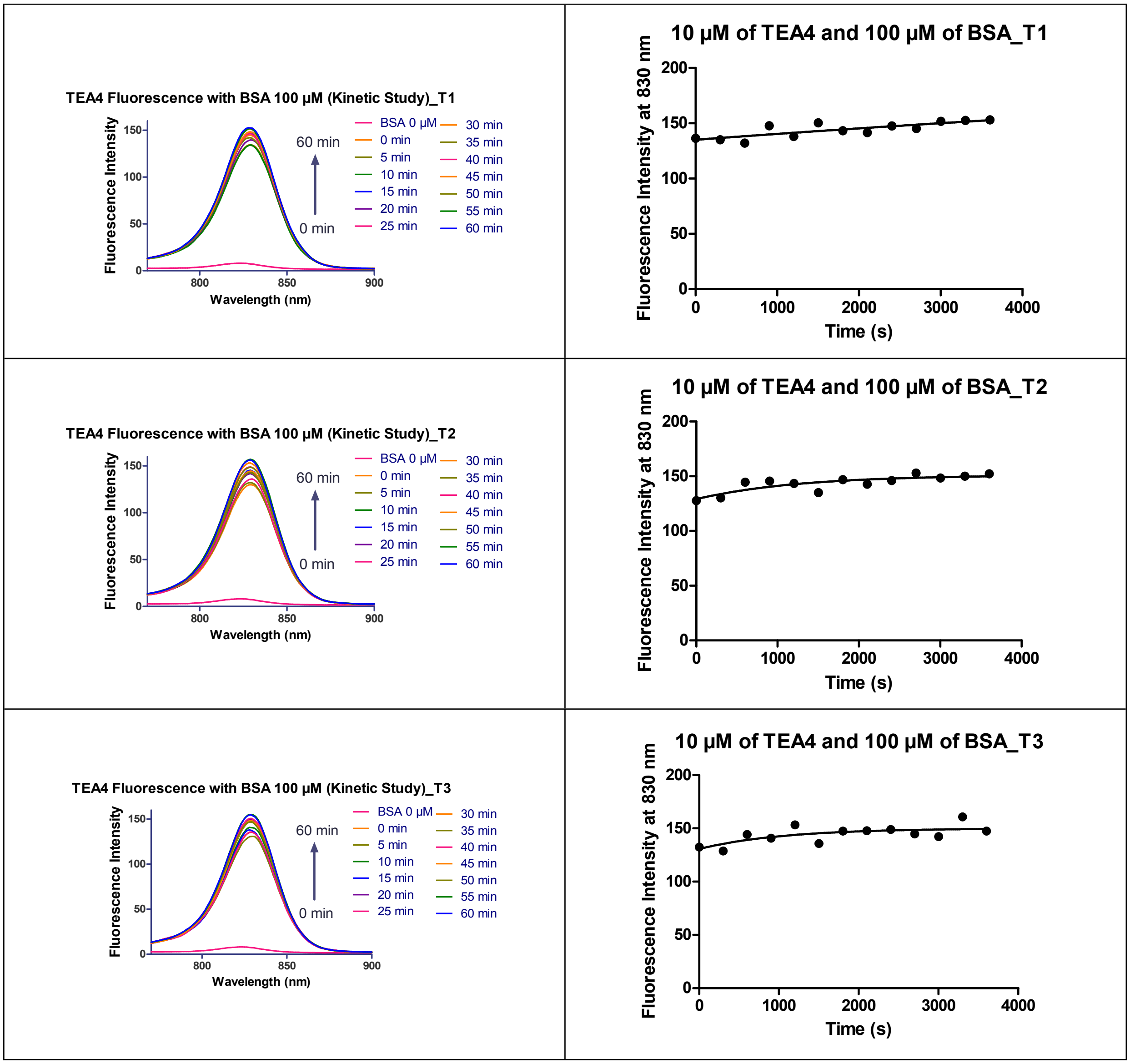

Figure S59. Fluorescence spectra of **TEA4** in PBS in the presence of 100 µM of **bovine serum albumin (BSA)** during a time interval of 60 minutes and plotting of the change in fluorescence with time. Each experiment was repeated 3 times (T1-T3)

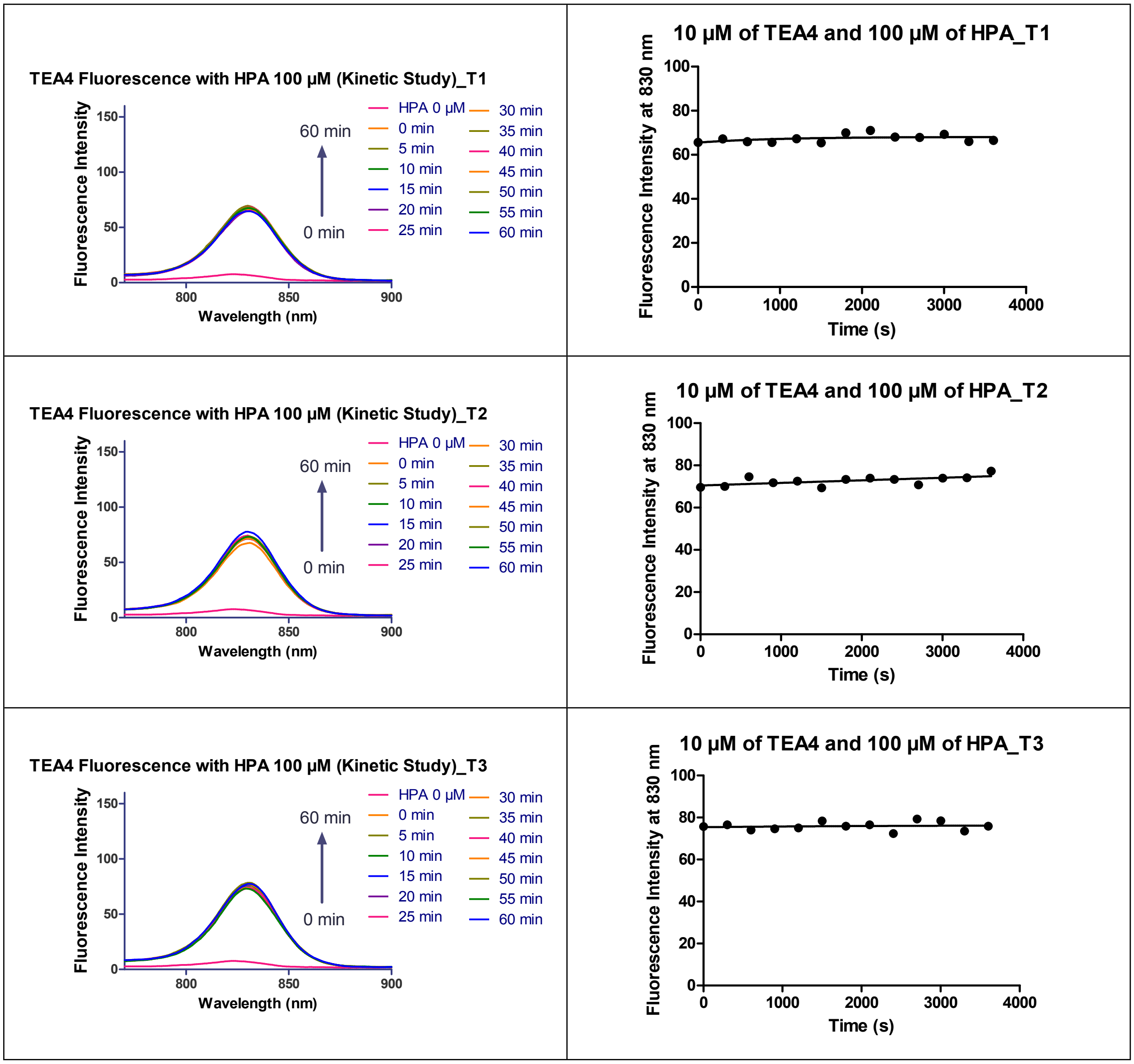

Figure S60. Fluorescence spectra of **TEA4** in PBS in the presence of 100 µM of **human parvalbumin (HPA)** during a time interval of 60 minutes and plotting of the change in fluorescence with time for the calculation of the pseudo first order rate constant (K) and the half-life of the interaction ($t_{1/2}$). Each experiment was repeated 3 times (T1-T3)

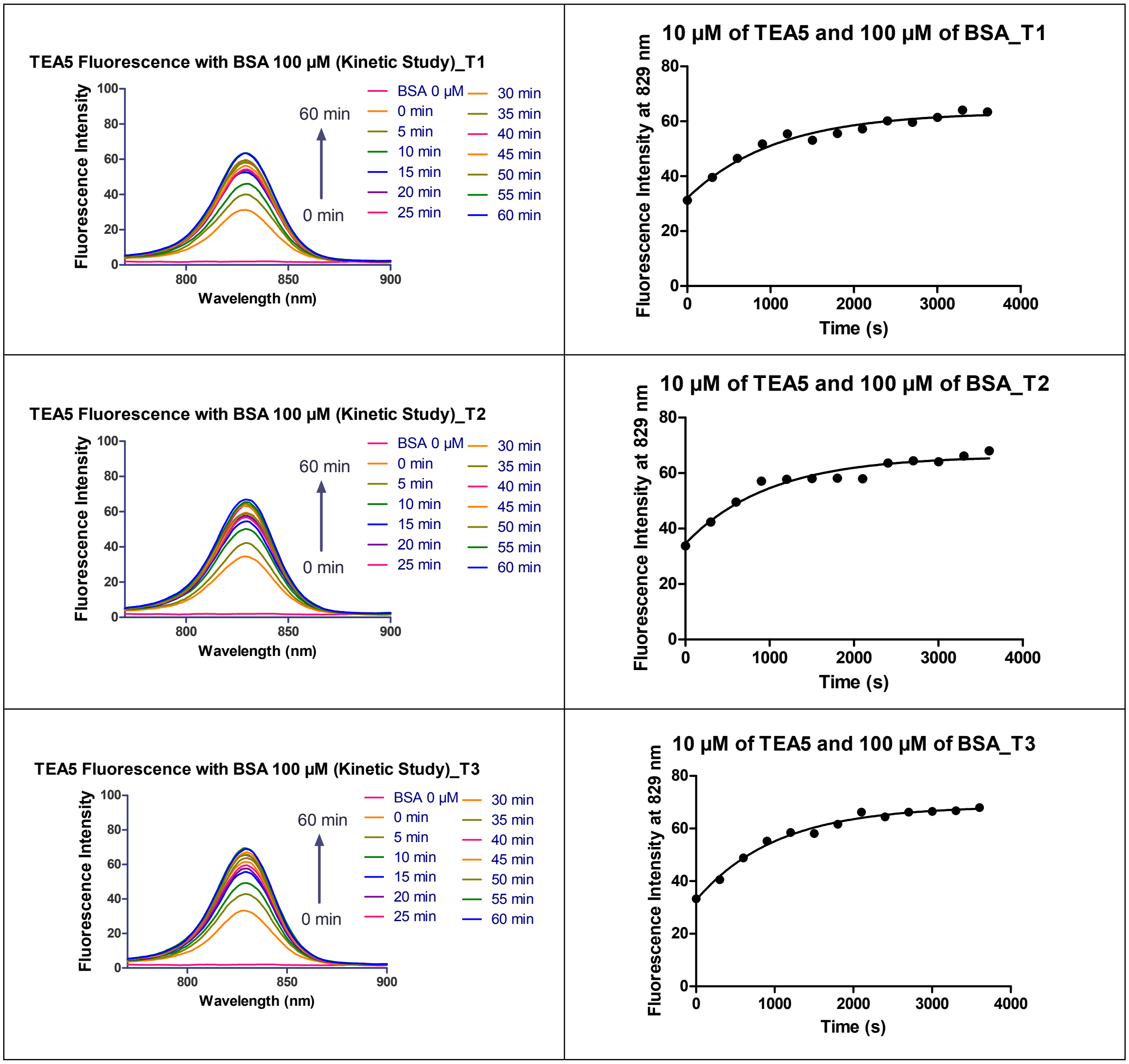

Figure S61. Fluorescence spectra of **TEA5** in PBS in the presence of 100 µM of **bovine serum albumin (BSA)** during a time interval of 60 minutes and plotting of the change in fluorescence with time for the calculation of the pseudo first order rate constant (K) and the half-life of the interaction ($t_{1/2}$). Each experiment was repeated 3 times (T1-T3)

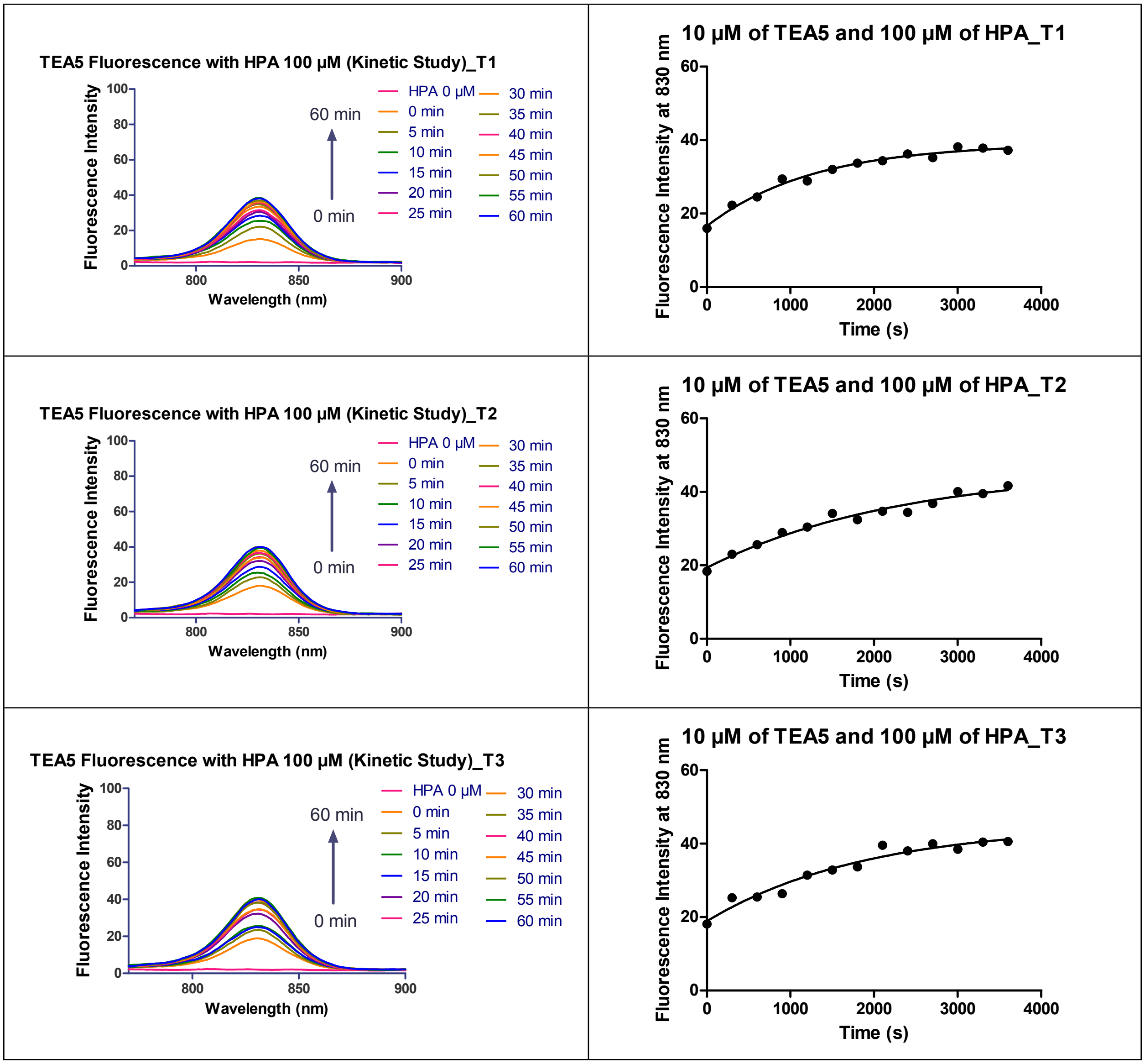

Figure S62. Fluorescence spectra of **TEA5** in PBS in the presence of 100 µM of **human parvalbumin (HPA)** during a time interval of 60 minutes and plotting of the change in fluorescence with time for the calculation of the pseudo first order rate constant (K) and the half-life of the interaction ($t_{1/2}$). Each experiment was repeated 3 times (T1-T3)

7.2. Kinetic analysis at different pH values

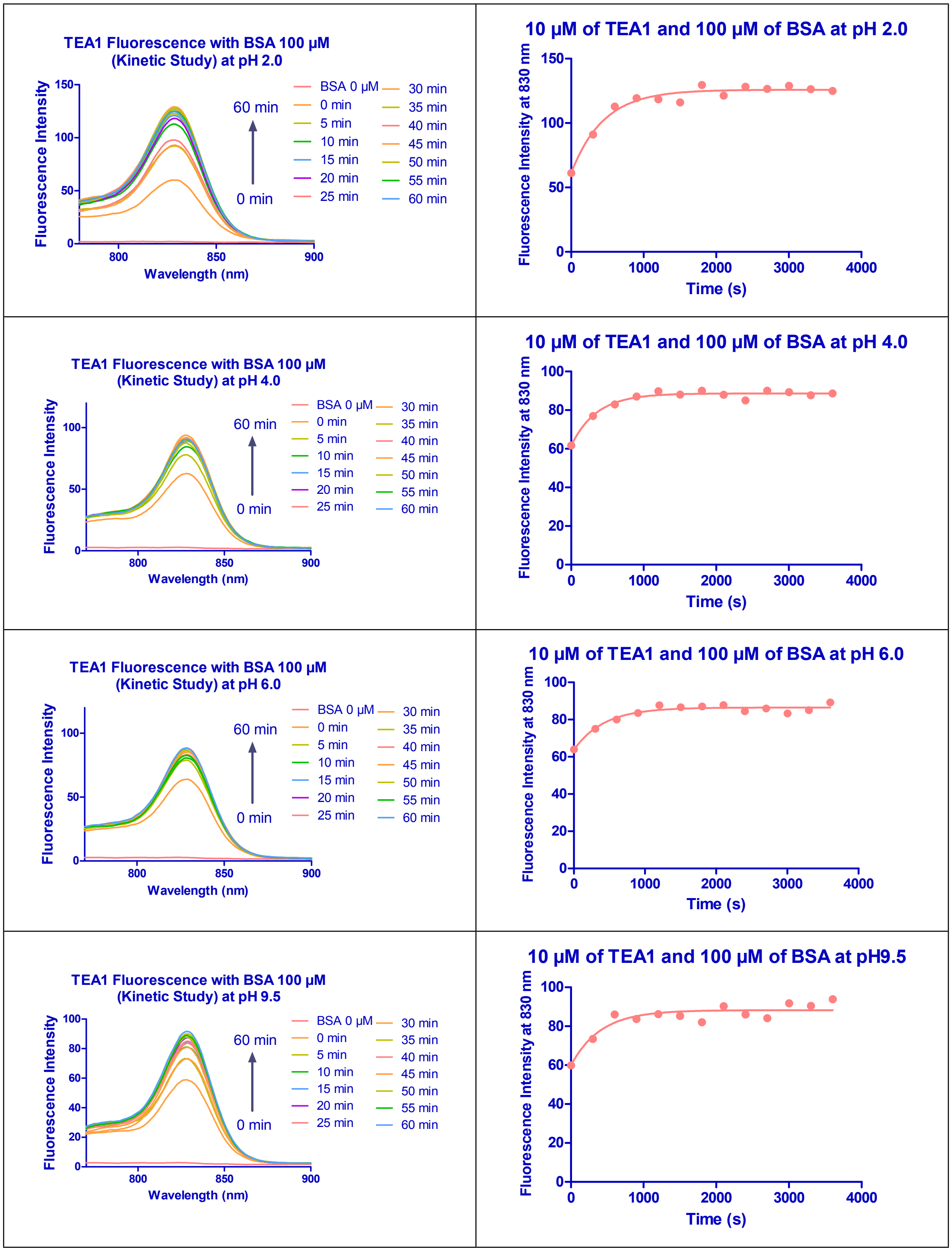

Figure S67. Fluorescence spectra of **TEA1** in PBS in the presence of 100 μM of **bovine serum albumin (BSA)** during a time interval of 60 min at different pH values, namely: pH 2.0, 4.0, 6.0, and 9.5

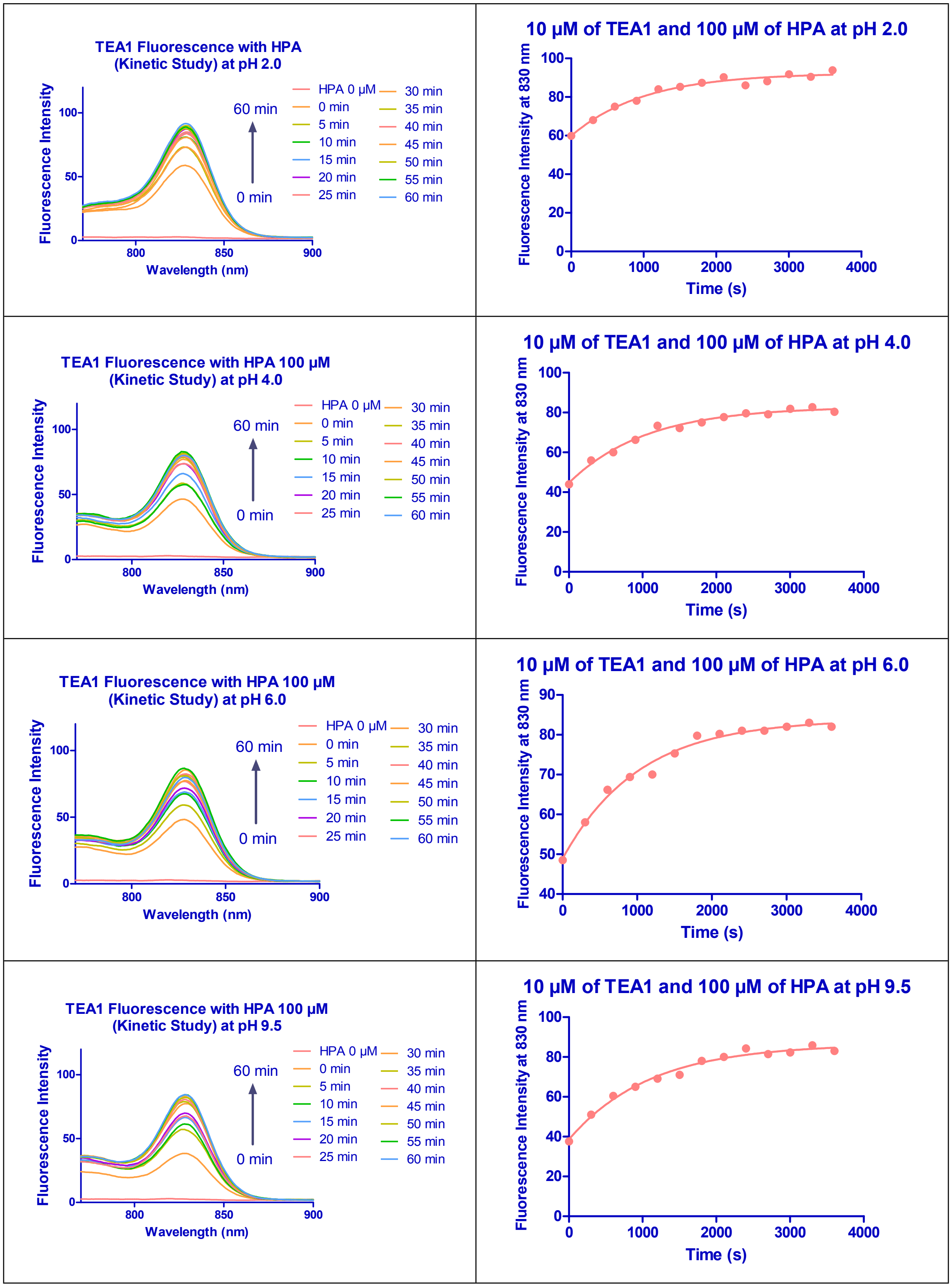

Figure S68. Fluorescence spectra of **TEA1** in PBS in the presence of 100 μM of **human parvalbumin (HPA)** during a time interval of 60 min at different pH values, namely: pH 2.0, 4.0, 6.0, and 9.5

7.3. Calculation of the binding constant of the dyes with BSA and HPA using the Benesi-Hildebrand plot

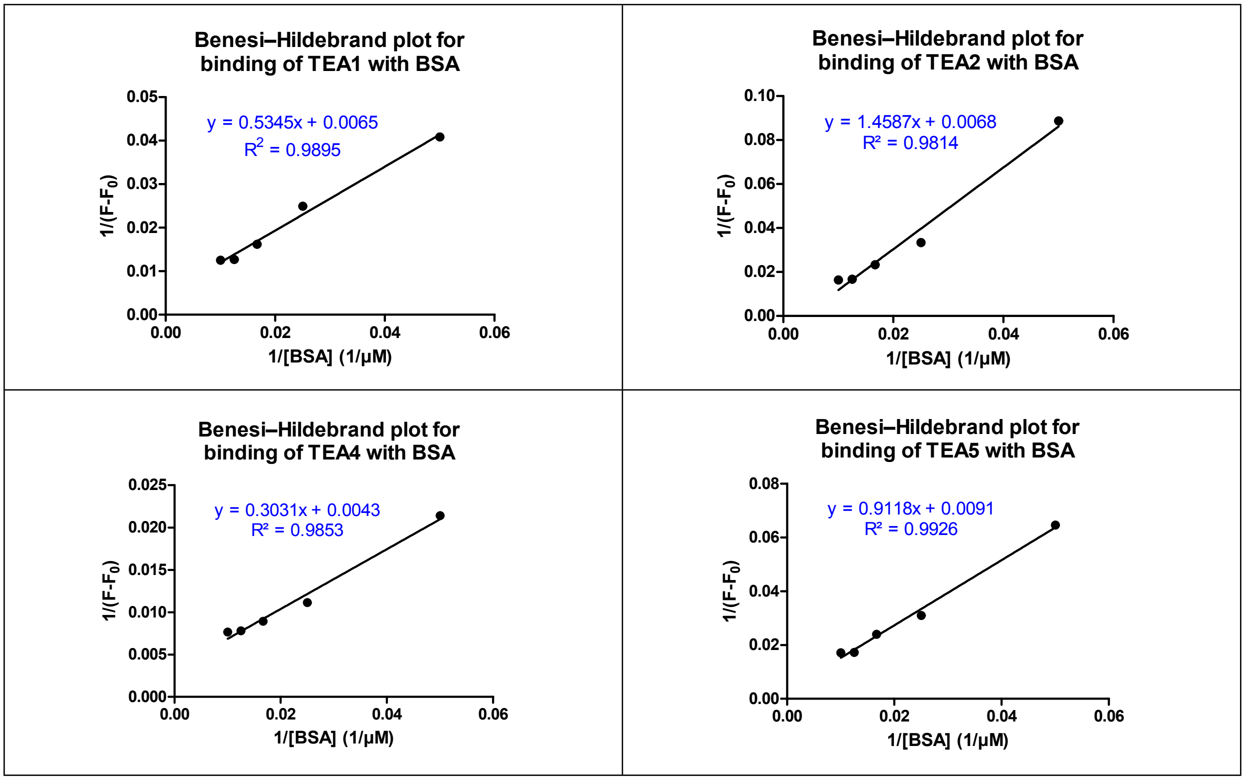

Figure S69. Benesi-Hildebrand plot of the effect of binding of **TEA1**, **TEA2**, **TEA4**, and **TEA5** with bovine serum albumin (BSA)

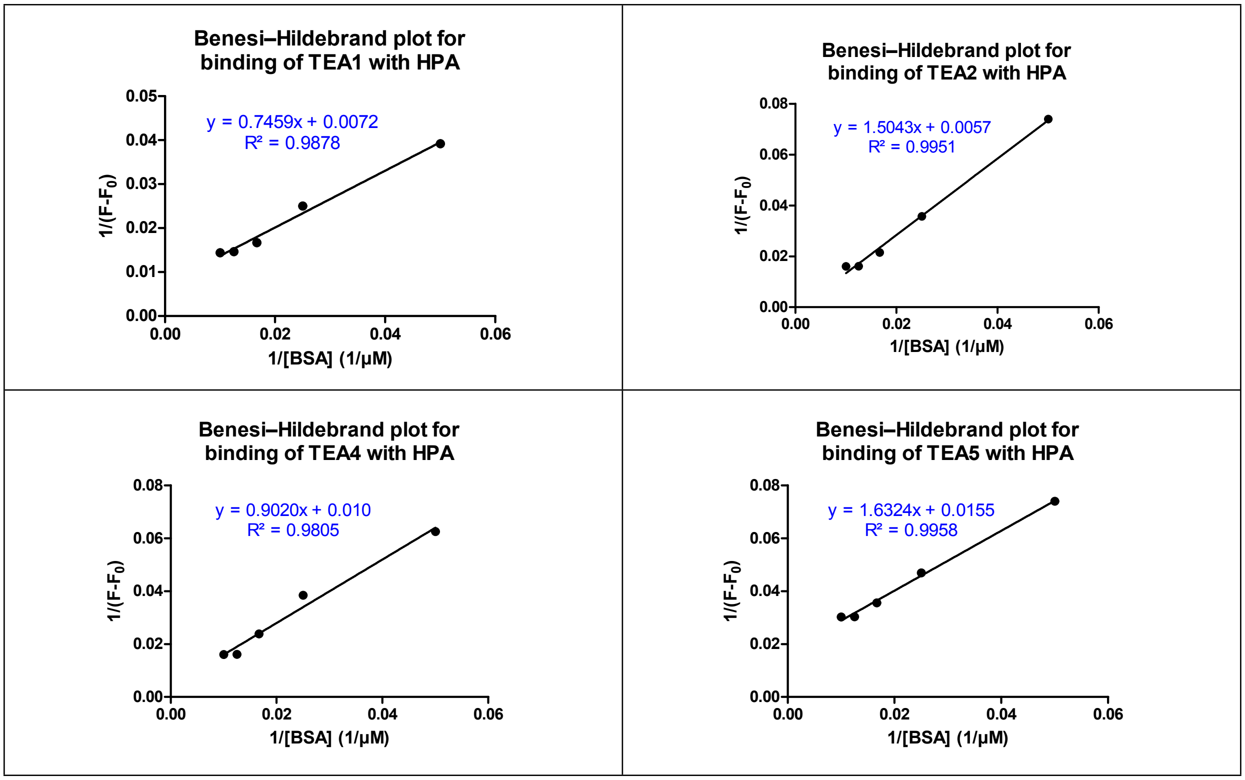

Figure S70. Benesi-Hildebrand plot of the effect of binding of **TEA1**, **TEA2**, **TEA4**, and **TEA5** with human parvalbumin (HPA)

7.4. Fluorescence intensity vs concentration of BSA or HPA for the calculation of limit of detection (LOD) and limit of quantitation (LOQ)

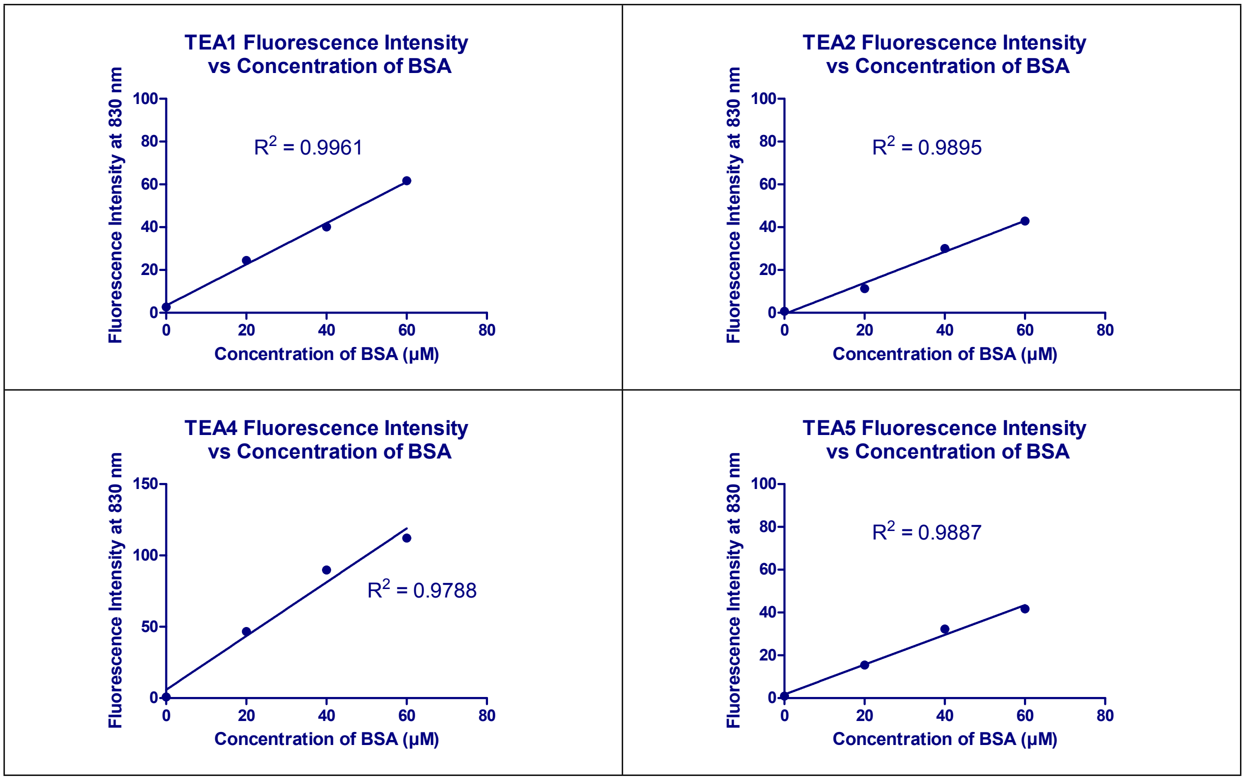

Figure S71. Fluorescence intensity of **TEA1**, **TEA2**, **TEA4**, and **TEA5** vs concentration of bovine serum albumin (BSA)

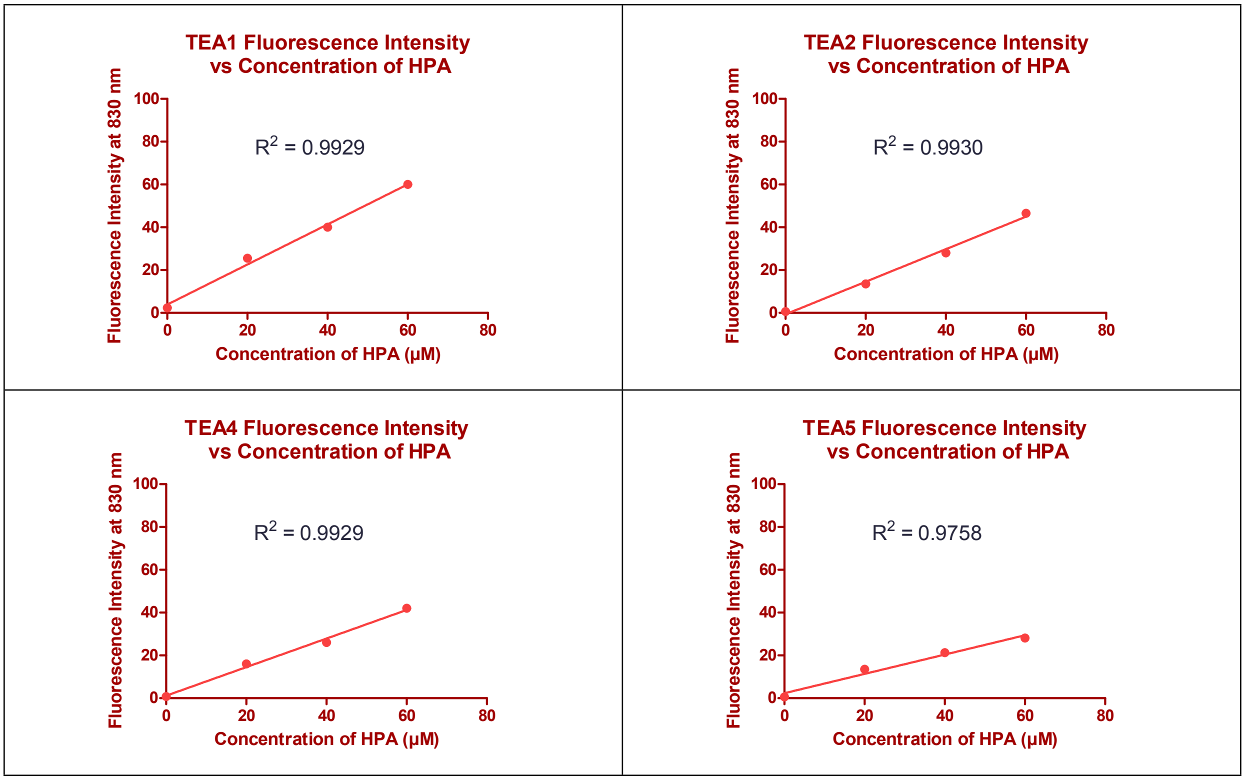

Figure S72. Fluorescence intensity of **TEA1**, **TEA2**, **TEA4**, and **TEA5** vs concentration of human parvalbumin (HPA)

7.5. Selectivity studies

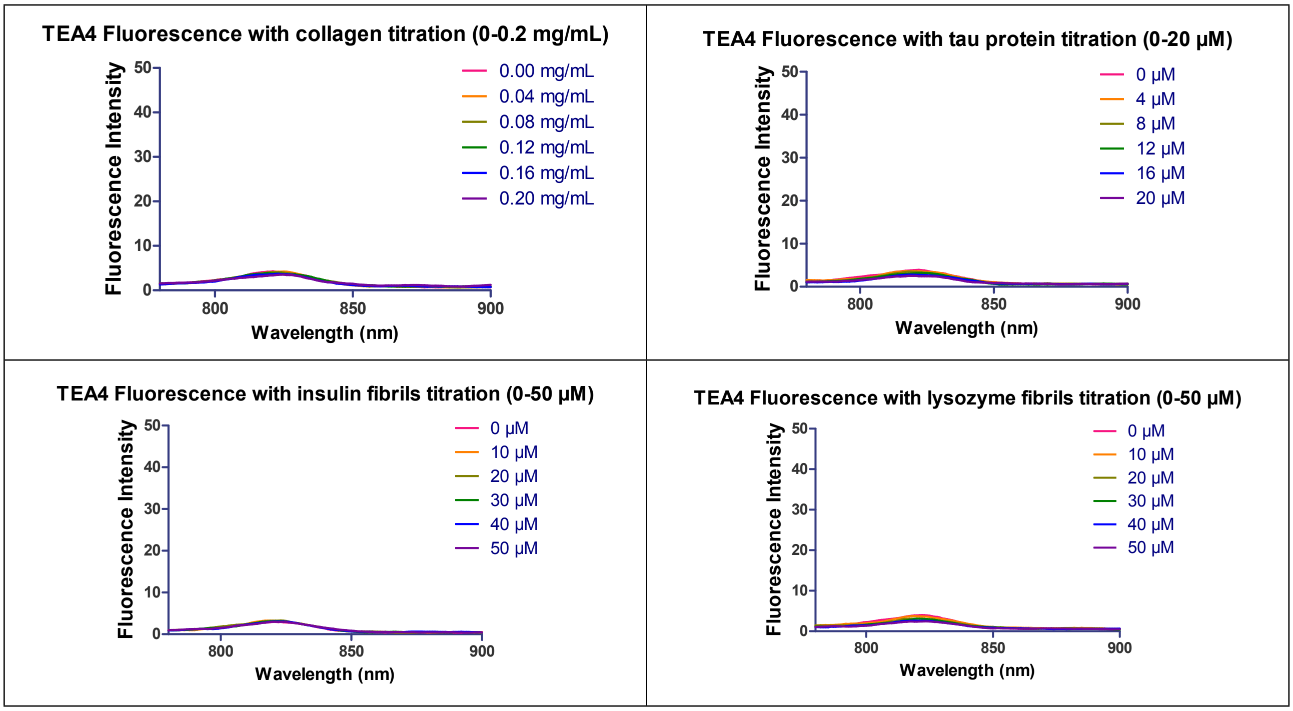

Figure S73. Fluorescence spectra of **TEA4** in PBS in the presence of increasing concentrations of different biomolecules

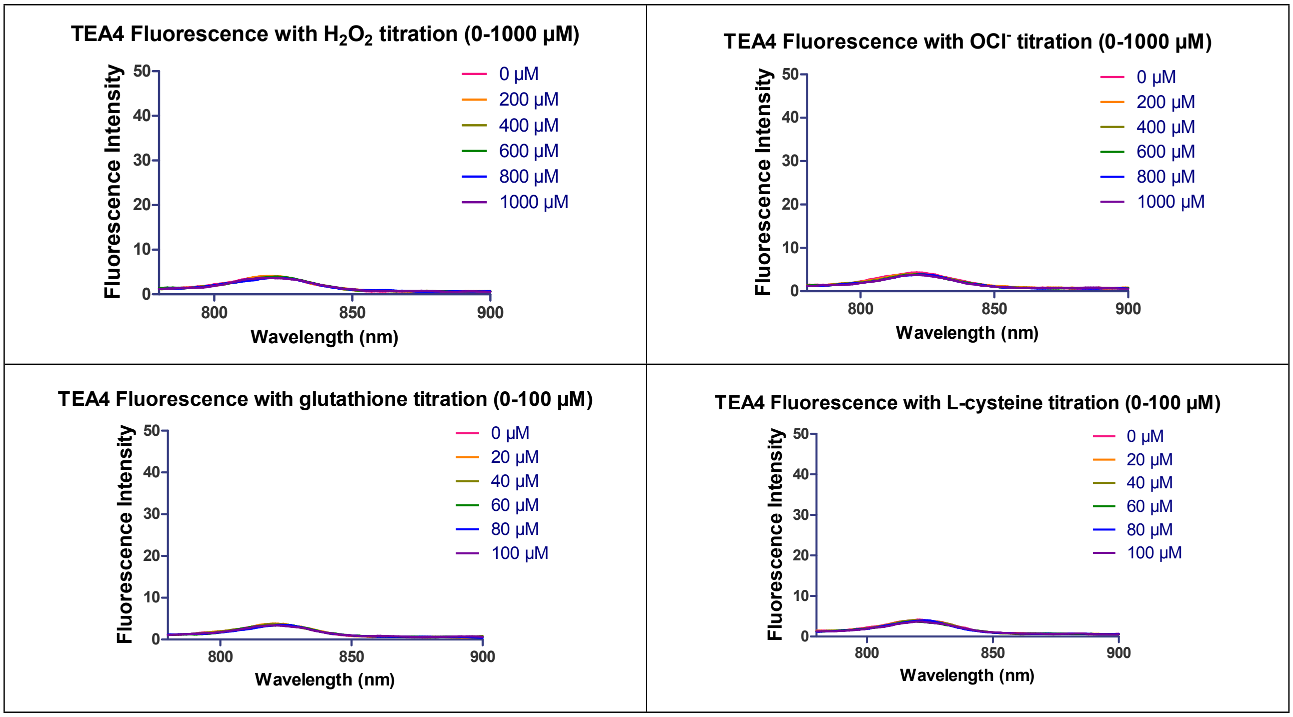

Figure S74. Fluorescence spectra of **TEA4** in PBS in the presence of increasing concentrations of different reactive oxygen species (ROS) or analytes

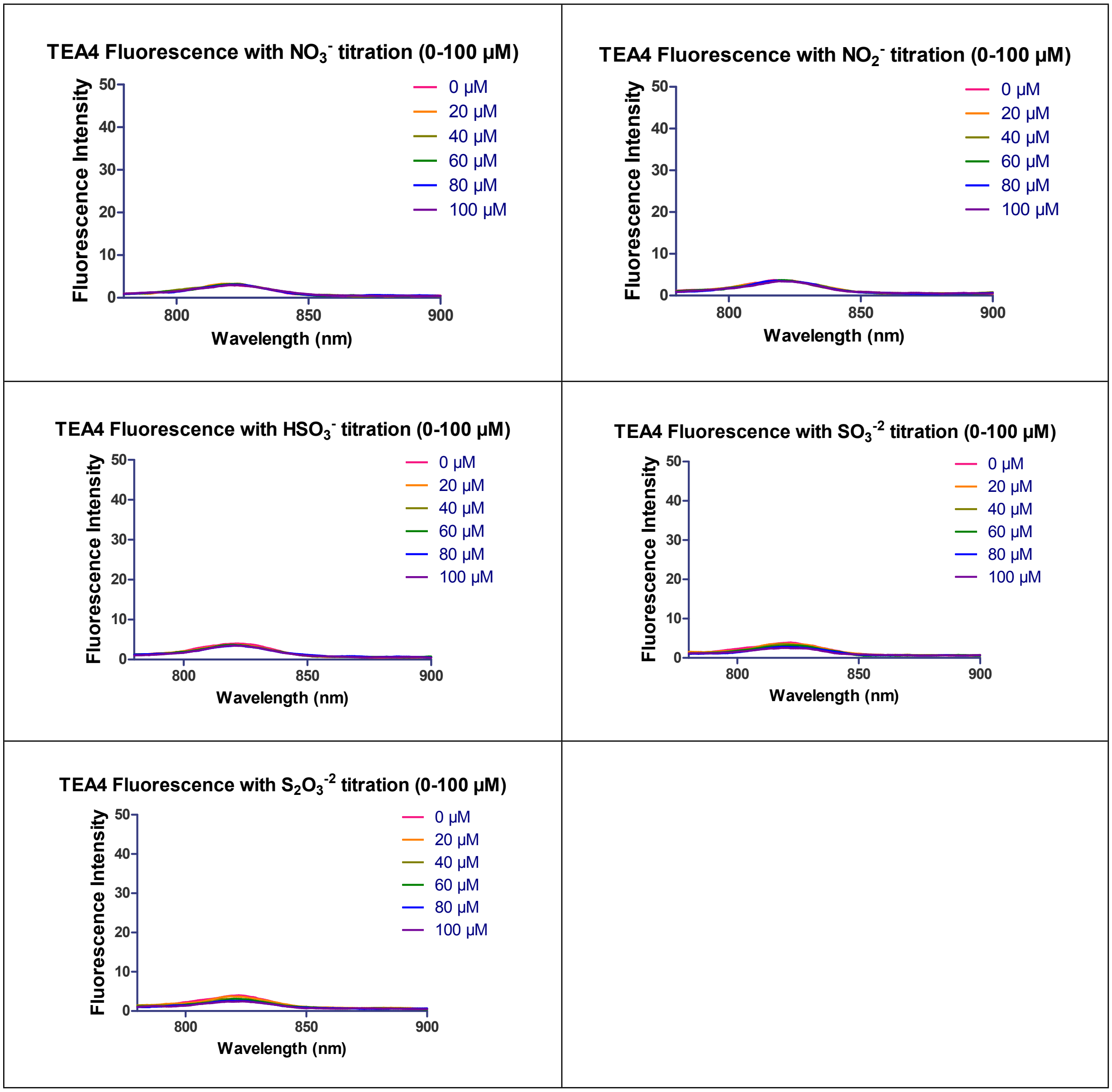

Figure S75. Fluorescence spectra of **TEA4** in PBS in the presence of increasing concentrations of different physiological ions

7.6. Viscosity sensing studies

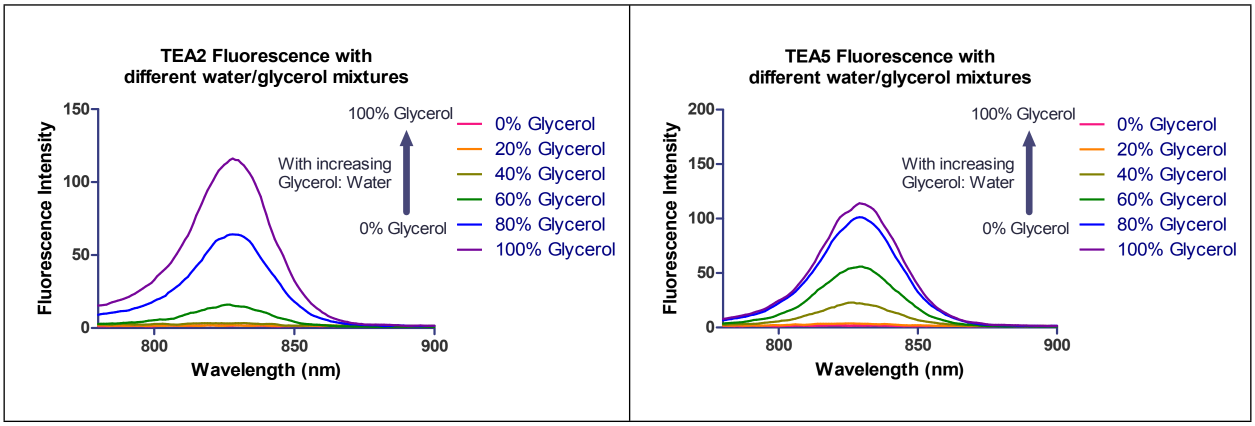

Figure S76. Fluorescence spectra of **TEA2,** and **TEA5** in solutions with increasing glycerol: water ratios.

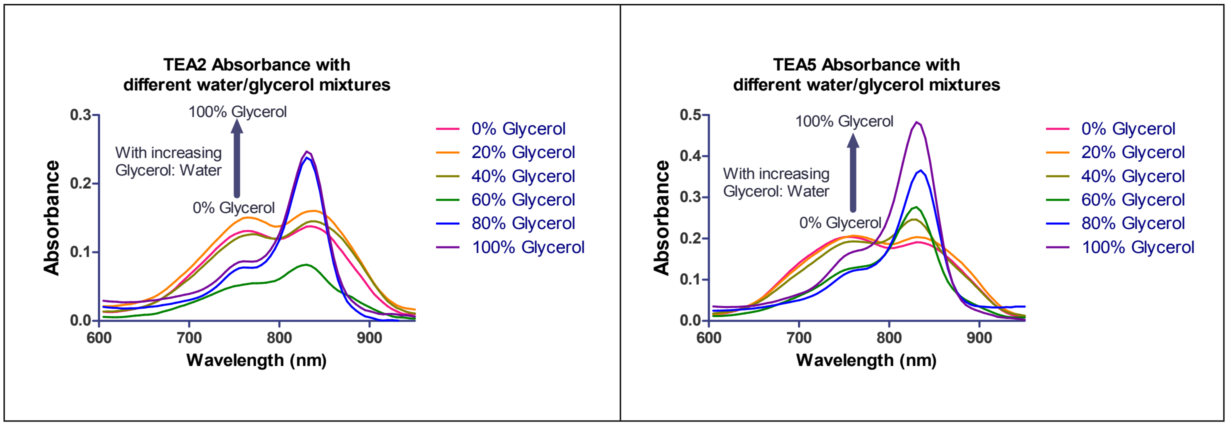

Figure S77. Absorbance spectra of **TEA2,** and **TEA5** in solutions with increasing glycerol: water ratios.
